# Exploring Scent Distinction with Polymer Brush Arrays

**DOI:** 10.1021/acsapm.5c00066

**Published:** 2025-03-07

**Authors:** Andriy R. Kuzmyn, Ivar Stokvisch, Gerrit-Jan Linker, Jos M. J. Paulusse, Sissi de Beer

**Affiliations:** † Department of Molecules & Materials, MESA+ Institute, University of Twente, Enschede 7500AE, The Netherlands; ‡ MESA+ Institute for Nanotechnology, University of Twente, Enschede 7522 NB, The Netherlands

**Keywords:** polymer brushes, SI-PET-RAFT, VOCs sensing, surface coatings, polymer coatings

## Abstract

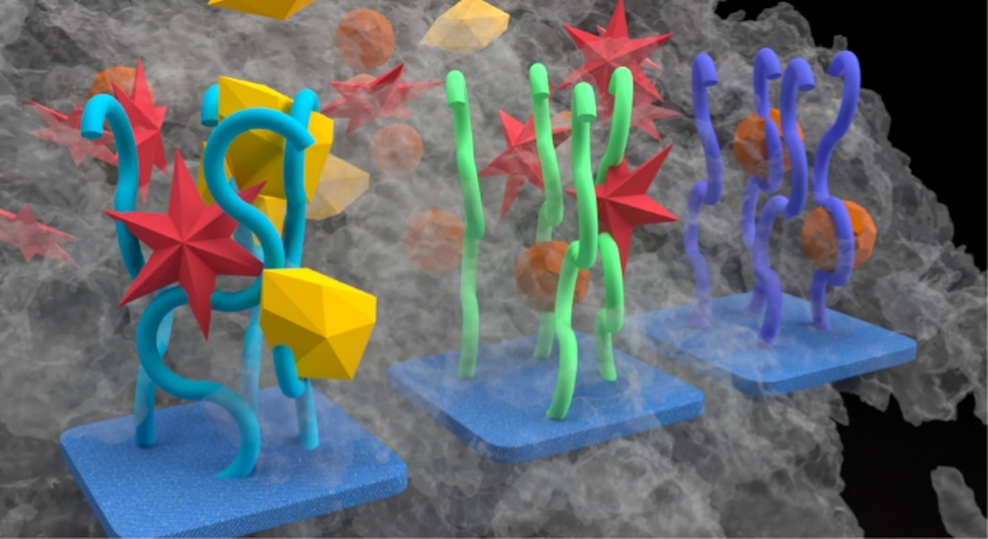

The ability to distinguish scents, volatile organic compounds (VOCs), and their mixtures is critical in agriculture, food safety, and public health. This study introduces a proof-of-concept approach for VOC and scent distinction, leveraging polymer brush arrays with diverse chemical compositions designed to interact with various VOCs and scents. When VOCs or scents are exposed to the brush array, they produce distinct mass absorption patterns for different polymer brushes, effectively creating “fingerprints”. Scents can be recognized without having to know the absorption of their individual components. This allows for a scent distinction technique, mimicking scent recognition within a mammalian olfactory system. To demonstrate the scent distinction, we synthesized different polymer brushes, zwitterionic, hydrophobic, and hydrophilic, using surface-initiated photoinduced electron transfer-reversible addition–fragmentation chain-transfer polymerization with eosin Y and triethanolamine as catalysts. The polymer brushes were then exposed to vapors of different single-compound VOCs and complex scents consisting of many VOCs, such as the water–ethanol mixture, rosemary oil, lavender oil, and whiskey scents. Quartz crystal microbalance measurements with dissipation monitoring (QCM-D) show a clear difference in brush absorption for these diverse VOC vapors such that distinct fingerprints can be identified. Our proof-of-concept study aims to pave the way for universal electronic nose sensors that distinguish scents by combining mass absorption patterns from polymer brush-coated surfaces.

## Introduction

The sense of smell, one of the five primary senses, plays a crucial role in the perception of odors. In the human body, this process occurs when odor molecules bind to receptors in the nasal cavity, transmitting a unique smell fingerprint to the brain.^1^ This ability to detect and interpret odors is instrumental in determining food quality, influencing taste, and alerting the reader to potential hazards. Most odors are composed of specific mixtures of volatile organic compounds (VOCs), which typically have a high vapor pressure at room temperature.^2,3^ Human activities can generate these, or they occur naturally. VOCs and their mixtures can represent various phenomena, including explosive compounds,^4^ insect infestation, fruit ripening,^5^ and various health conditions.^6,7^ Therefore, the potential ability to distinguish VOCs and scents holds significant implications for public health, environmental monitoring, public safety, agriculture, and food production.^2^

The biological nose is a sophisticated olfactory system. So far, only 50 out of the roughly 300–400 functional olfactory receptors have been identified, indicating we are still far from fully unraveling the human olfactory system.^8−10^ The nose has not only high sensitivity but also ways to amplify the signal of the scent moieties. There have been numerous attempts to mimic the human nose, ranging from organic and inorganic gas sensors^11−15^ to biosensors incorporating proteins^16^ or parts or whole of insects.^17,18^ The sensors based on inorganic or organic coatings generally provide good sensitivity to one or several target analytes but often display poor specificity.^11^ Biobased sensors provide specificity but are expensive and more challenging to produce.^10^

There are three main types of transducers for VOC sensing: electrical, optical, and gravimetric. Often, the transducer is coated with sensing materials such as metal oxides,^19^ polymers,^20^ self-assembled monolayers,^21^ and nanostructured materials.^22^ Those coating materials increase the surface area and allow more specific detection of analytes.^23^ Polymer brush coatings introduce high swelling behavior, and their high surface area could aid as selective absorbers to enhance sensitivity and specificity.^24−33^ Polymer brush coatings consist of macromolecules attached to a surface with a high surface density by a chain end or ends.^34−39^ Previously, polymer brush coatings were applied not only for vapor sensing^28^ but also as antifouling,^40−47^ antibacterial,^42,48,49^ antiviral,^50^ bioactive,^51,52^ biointeractive,^44,53^ photosensitive,^54^ cell-interactive,^55,56^ biomimetic, and/or lubricating surfaces.^57−59^

Previously, the development of polymer brush coatings was hampered by complex synthesis procedures.^45,46^ Typically, reversible deactivation radical polymerization (RDRP) techniques such as surface-initiated atom transfer radical polymerization (SI-ATRP),^46,47,60,61^ single-electron transfer-living radical polymerizations (SET-LRP), and light-triggered living radical polymerization (LT-LRP)^51^ are applied for the synthesis of polymer brushes. Often, those approaches require oxygen-free conditions and heavy metal catalysts. However, the development of surface-initiated activators regenerated by the electron transfer atom transfer radical polymerization (SI-ARGET-ATRP)^43,62,63^ allowed for a lower metal catalyst concentration. The introduction of surface-initiated photoinduced electron transfer-reversible addition–fragmentation chain transfer (SI-PET-RAFT)^40,64−66^ allowed the conduct of polymer brush synthesis in simple and mild conditions. This approach can create polymer brushes in an aqueous environment using an edible photocatalyst and visible light.

**Scheme 1:**
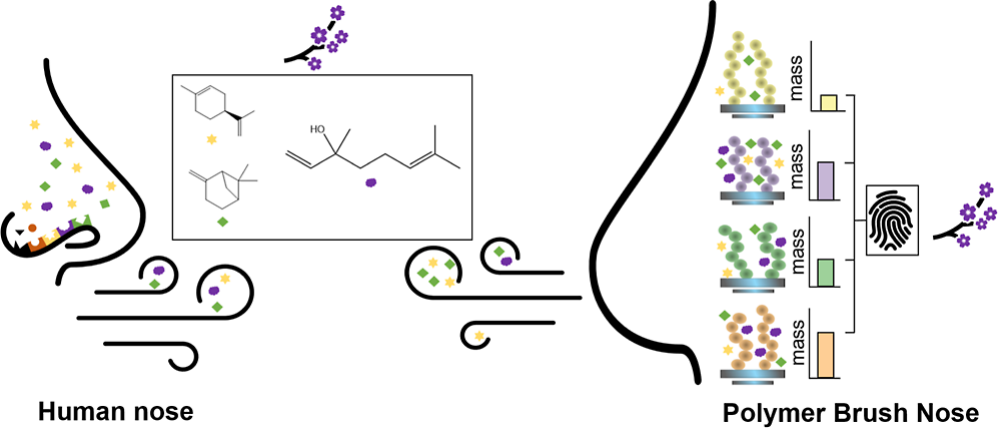
General Scheme for Distinguishing Scents and Vapors Using a Polymer Brush Array The diagram begins with the human nose on the left, which contains various receptors that detect specific scents. In the center, the scent of lavender oil consisting of VOCs and their molecular components are shown, with each molecule represented by distinct shapes and colors. On the right, our polymer brush electronic nose is depicted. The polymer brush coatings work by adsorbing different molecules, such as those found in lavender oil. The polymer brush coatings capture these molecules based on their mass, producing a unique “fingerprint” pattern when identifying the lavender oil smell

Herein, we apply SI-PET-RAFT techniques to create an array of four polymer brushes to distinguish different scents and vapors (Scheme 1, right side). The polymer brush array is based on carboxybetaine methacrylamide (CBMA), *N*-(2-hydroxypropyl) methacrylamide (HPMA), oligo(ethylene glycol) methacrylate (MeOEGMA), and butyl methacrylate (BMA). The physicochemical properties of obtained polymer brushes were characterized using X-ray photoelectron spectroscopy (XPS), ellipsometry, and atomic force spectroscopy (AFM). The poly(BMA) brushes were created for the first time using SI-PET-RAFT in this work. The modified surfaces were further exposed to vapors composed of different VOCs. The absorption patterns of different VOC vapors were investigated using quartz crystal microbalance measurements with dissipation monitoring (QCM-D). We demonstrate here the applications of arrays of polymer brushes with varying chemical compositions to distinguish different VOCs and their mixtures. Inspired by the human nose, we look for a smell pattern in the absorption of different VOCs on a polymer brush array. The advantage is that using arrays of polymer brushes allows us to distinguish scents without needing to know which molecules absorb in the brushes. This enables the differentiation of unique scent “fingerprints”. We exposed our polymer brush array to different common single compound VOCs such as acetone, limonene, ethanol, 2-propanol, 3-methyl-1-butanol, carvone, α-pinene, as well as scents that consist of numerous VOCs such as the relevant scent of the water–ethanol mixture, rosemary oil, lavender oil, and whiskeys. Combining mild and scalable SI-PET-RAFT techniques with polymer brush arrays marks the first time this approach has been used for distinguishing scents. This proof-of-concept paves the way for future sensor applications in scent and VOC detection.

## Results and Discussion

### Polymer Brush Synthesis and Characterization

**Scheme 2:**
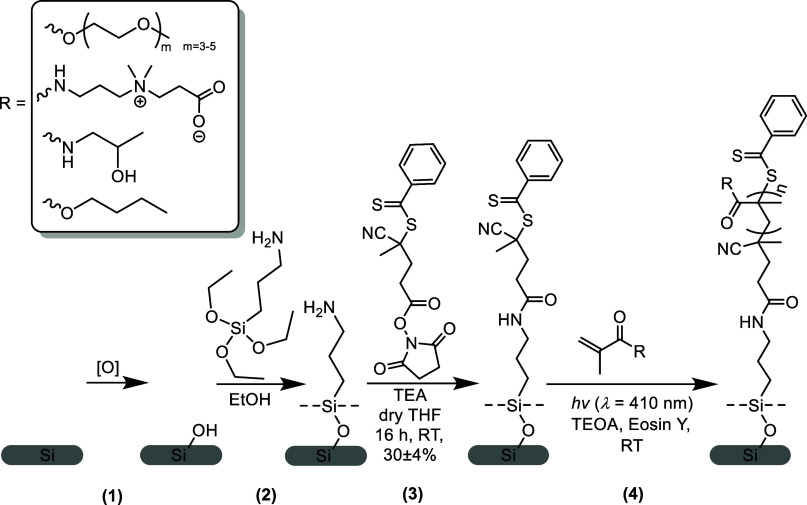
Overview of the SI-PET-RAFT Polymer Brush Coating Synthesis Process Polymer brushes composed of MeOEGMA, CBMA, HPMA, and BMA were synthesized through a four-step procedure: (1) plasma cleaning of silicon surfaces, (2) immobilization of APTES, (3) attachment of the NHS-RAFT agent, and (4) polymer brush synthesis facilitated by EY and TEOA

Our research has involved the application of the SI-PET-RAFT technique to prepare polymer brush-based coatings for volatile organic compounds (VOC) and scent sensing. Four different monomers were selected, i.e., carboxybetaine methacrylamide (CBMA) *N*-(2-hydroxypropylhydroxypropyl) methacrylamide (HPMA), methoxy-oligo(ethylene glycol) methacrylate (MeOEGMA), and butyl methacrylate (BMA), to prepare arrays of polymer brush coatings, which were designed to cover a range of affinities from highly hydrophilic (static water contact angle (SWCA): 20°) to hydrophobic (SWCA: 80°). The polymerization technique by SI-PET-RAFT of the first three monomers was previously introduced.^40,44,50,55,65^ The synthesis of polymer brushes followed a three-step procedure. First, we oxidized the silicon surfaces and immobilized (3-aminopropyl)triethoxysilane (APTES) moieties on the surface. Next, we modified the APTES layer with the 4-cyano-4-(phenylcarbonothioylthio)pentanoic acid *N*-succinimidyl ester (NHS-RAFT agent). The conversion of this surface reaction was previously reported to be at 30 ± 4%.^40,44^ The relative bulkiness of the RAFT-NHS molecule compared to APTES may sterically hinder the immobilization of the RAFT agent on the surface.^40,44^ Active ester surface reaction chemistries may yield low efficiency due to reaction limitations and the bulkiness of the reactants. Optimizing reaction conditions or using smaller RAFT agents could improve yields; however, effective RAFT agents often require bulky groups, such as phenyl or pyridine. In the final step, the polymer brushes were grown from RAFT-functionalized surfaces in the presence of Eosin Y and TEOA as catalysts and under visible light irradiation (Scheme 2).

**Figure 1:**
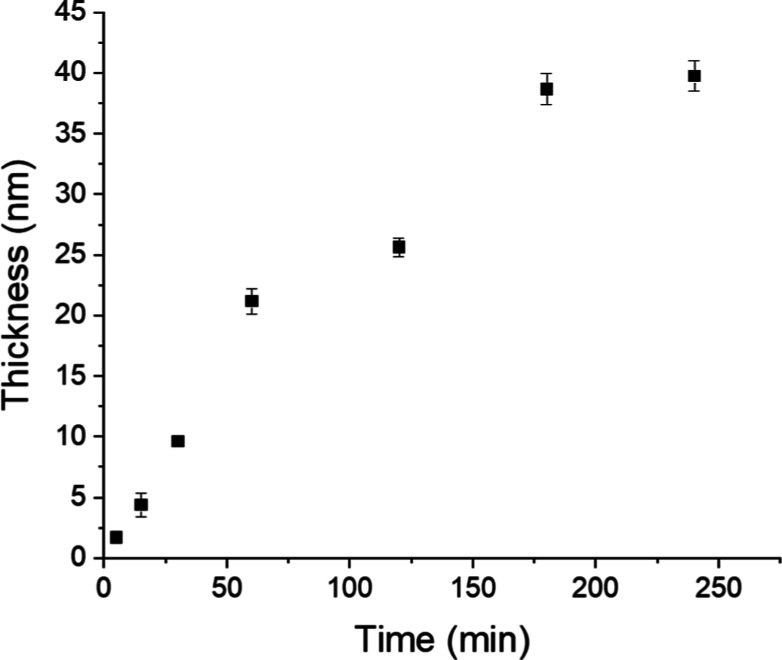
Kinetics of poly(BMA) brush growth. To the best of our knowledge, this study is the first to demonstrate the SI-PET-RAFT procedure for BMA monomer, utilizing EY and TEOA as photocatalysts. The kinetics and evolution of polymer brush growth were analyzed to confirm the control over the polymerization process further.

The procedure of preparing poly(BMA) brushes by SI-PET-RAFT is demonstrated here for the first time, to the best of our knowledge. We chose DMSO as the solvent for polymerization due to the monomer’s poor solubility in water. Polymerization in EtOH and EtOH/water mixtures resulted in thicknesses no greater than 5 nm and exhibited poor control. However, polymerizing BMA in DMSO achieved reasonable control within the first 2 h, and then it seems to be plateauing (Figure 1). This was previously observed for poly(CBMA), poly(MeOEGMA), and poly(HPMA).^40,65^ This is probably related to a relative monomer deficiency due to the increased viscosity of the polymerization solution. Those aspects can be improved using SI-PET-RAFT polymerization in flow or refreshing the polymerization solution.^65^ The loss of polymerization control can also be related to the termination and partial oxidation of the RAFT agent at the surface and photocatalyst. The grafting densities previously for the SI-PET-RAFT technique were reported at a range of 0.14 chains per nm.^25,5,65^ Theoretically, absorption is expected to depend on grafting density;^36,67^ however, experimental results suggest that this effect is relatively minor.^39^

The polymer brushes of poly(CBMA), poly(HPMA), poly(MeOEGMA), and poly(BMA) were characterized by using AFM, XPS, ellipsometry, and contact angle. We used polymer brushes after 2 h of polymerization, yielding thicknesses from 18 to 47 nm, depending on the monomer unit (Table 1). The resulting thicknesses of polymer brushes align with previously reported kinetics for poly(CBMA), poly(HPMA), and poly(MeOEGMA) on silicon surfaces. The AFM topography images (Supporting Information Table S7) of brush-coated surfaces through the range of thicknesses from 18 to 47 nm revealed homogeneous layers with root-mean-square roughness (*R*q) from 2.8 to 5.6 nm.

**Table 1:** Physico-Chemical Properties of the Polymer Brush-Coatings

polymer brush	thickness (nm)	SWCA (deg)	surface energy (mN·m^–1^)	RMS roughness (nm)
poly(CBMA)	18 ± 1	20 ± 1	78 ± 1	2.9 ± 0.1
poly(HPMA)	47 ± 3	44 ± 1	66 ± 1	2.8 ± 0.1
poly(MeEOGMA)	36 ± 3	47 ± 1	64 ± 1	5.6 ± 1.1
poly(BMA)	25 ± 1	88 ± 1	36 ± 1	4.7 ± 0.9

**Figure 2:**
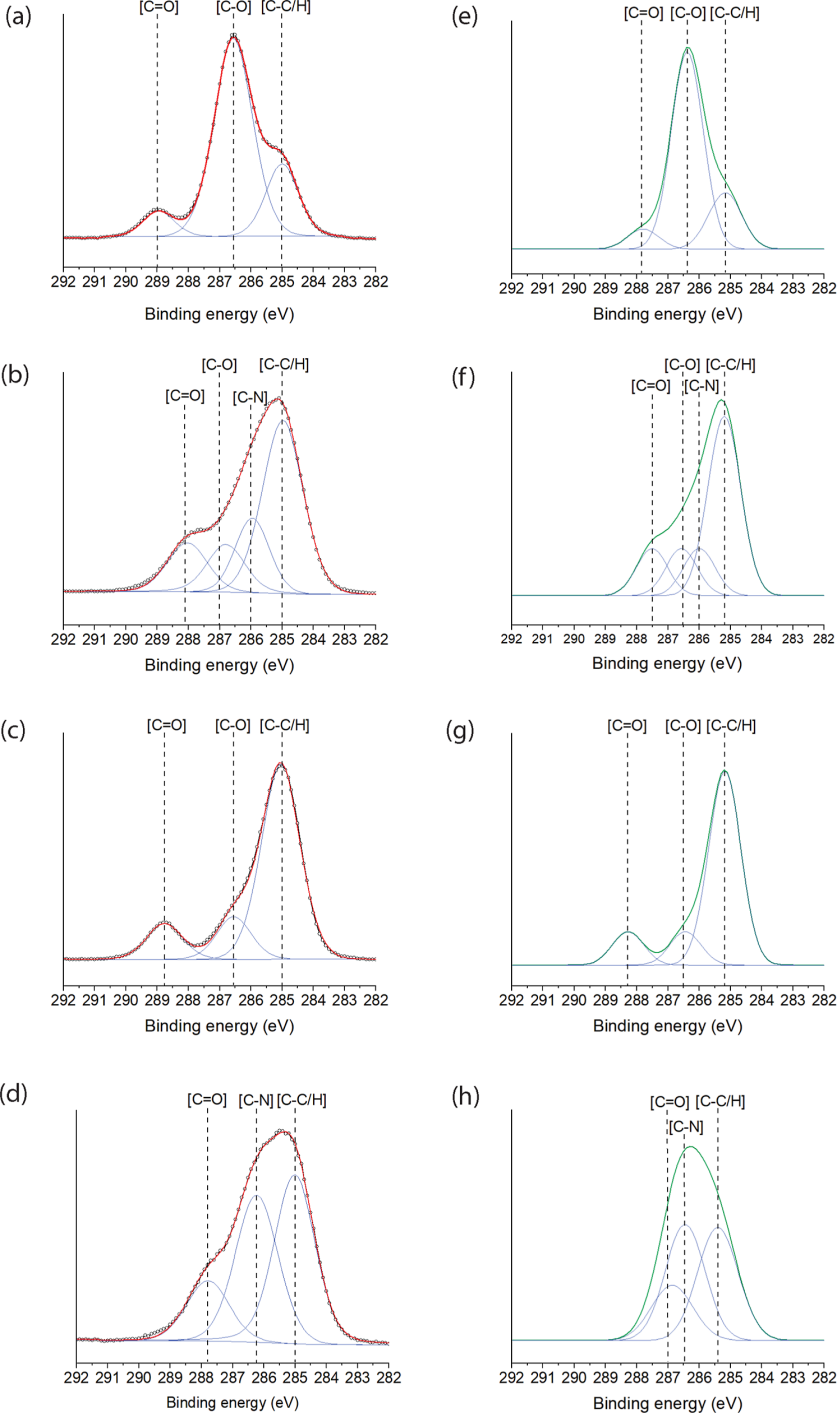
Narrow C 1s XPS spectra of poly(BMA) (a), poly(HPMA) (b), poly(MeOEGMA) (c), and poly(CBMA) (d), along with the corresponding computed spectra for these monomers (e–h). The narrow C 1s spectra were analyzed to validate the chemical composition of the polymer brushes, with experimental results corroborated by simulated XPS spectra for each monomer. This combination of experimental and simulated data provides robust confirmation of the polymer brush compositions.

We characterized the chemical composition of the polymer brush layers created using X-ray photoelectron spectroscopy (XPS)(Figure 2) (see Supporting Information XPS characterization section and Table S1). We measured the wide or survey spectrum and narrow C 1s spectra of all the polymer brush layers created. Furthermore, spectrum simulations were performed by using density functional theory (DFT) calculations to assist in the peak assignment. The DFT simulation of XPS spectra improves the quality of the fit. (See Supporting Information for computational details of DFT calculations and Tables S2–S6). The ab initio DFT simulation has enabled an improvement in the quality of the fit XPS spectra.

### Volatile Organic Compound (VOCs) Sensing

**Scheme 3:**
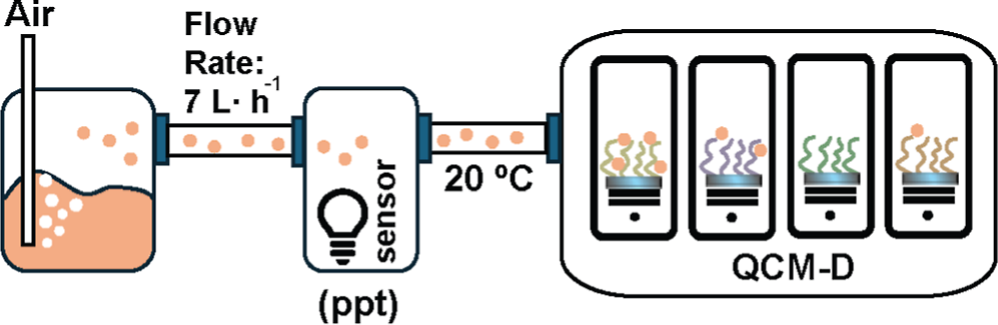
Schematic Representation of the QCM-D Vapor Absorption Setup The schematic depiction illustrates a bubbler system containing the liquid that generates vapor (e.g., lavender oil, ethanol). Air, flowing at a controlled rate of 7 l·h^–1^, was passed through the target liquid to produce vapor. The vapor concentration was measured using an Ion Science MiniPID2 sensor for single VOC compounds. Subsequently, the vapor was directed over the QCM-D chip coated with the corresponding polymer brushes to assess vapor absorption properties. All the components of the setup were at 20 °C to prevent condensation

**Figure 3:**
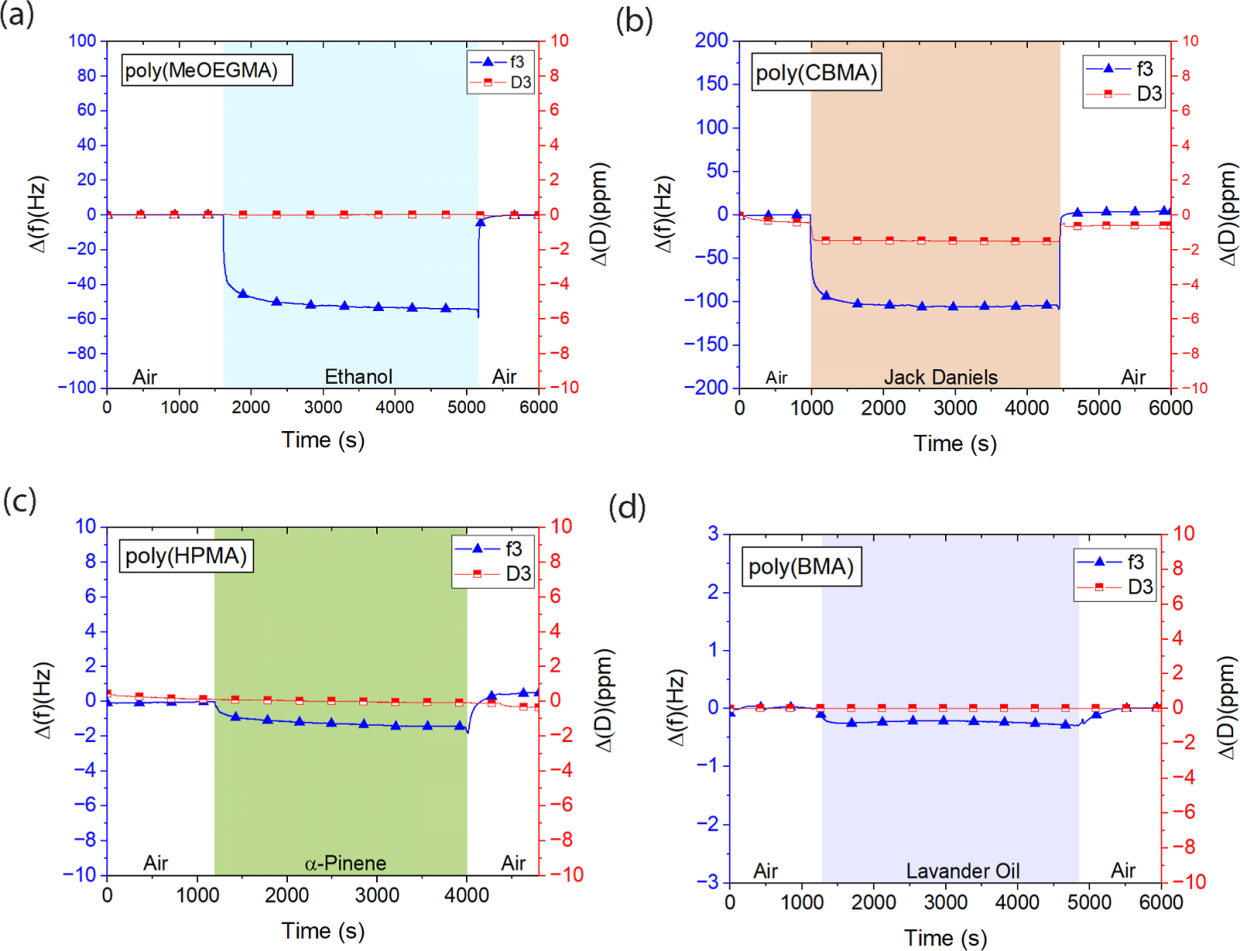
Representative sensograms and QCM-D sensorgrams of poly(MeOEGMA) exposure to ethanol vapor (EtOH) (12 ppt). (a) Poly(CBMA) exposed to vapor of Jack Daniels whiskey, (b) poly(HPMA) exposed to α-pinene vapor (1.2 ppt), (c) and poly(BMA) to lavender oil vapor. (d) The Δ(*f*) is the change frequency in third harmonics (*f*3), and Δ(*D*) is the change in dissipation in third harmonics (*D*3) in the QCM-D sensogram. Please also see for a complete overview of sensorgrams Supporting Information Table S8–S17 and S19–S24 for each of the vapors and brushes.

**Figure 4:**
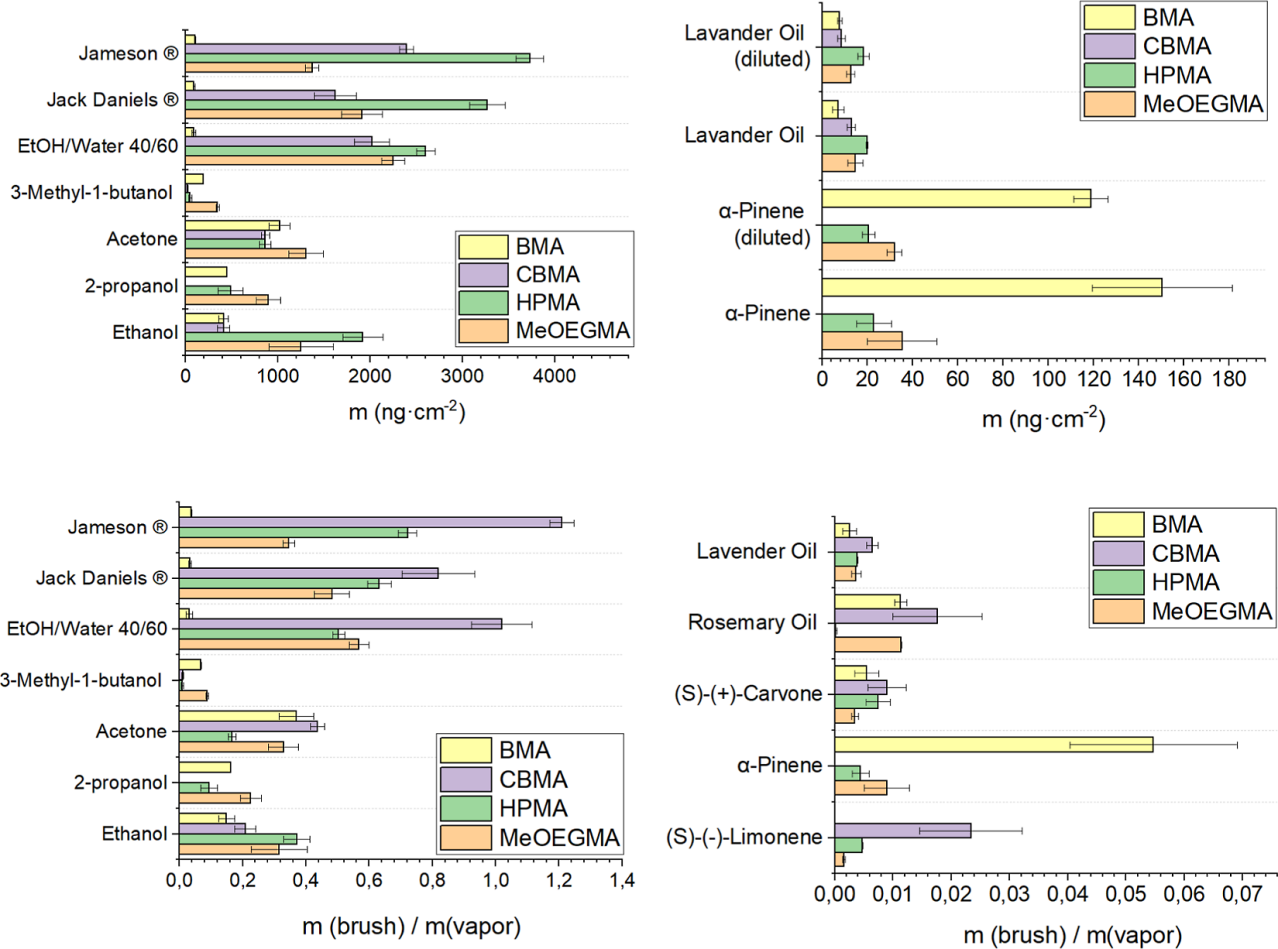
Measured added mass for poly(BMA), poly(CBMA), poly(HPMA), and poly(MeOEGMA) brushes after exposure to the different VOCs and scents given in the graph and ratio between the mass of absorbed vapor and mass of the polymer brush coatings. The overview of absorbed mass on all four polymer brushes showed each scent’s possible “fingerprint”.

The arrays of QCM crystals coated with poly(BMA), poly(CBMA), poly(MeOEGMA), and poly(HPMA) polymer brushes were exposed to vapors of different VOCs and scents. VOCs were selected with different polarity and, consequently, potential affinity to chemically different polymer brushes. In a typical experiment, the QCM-D chips coated with polymer brushes were stabilized by flowing dry air at room temperature (20 °C) for 5–10 min, following injection of the vapor of different compounds for approximately 1 h (Scheme 3), which we checked to be enough to reach equilibrium. The concentration of vapor of single VOCs was determined by an Ion Science MiniPID2 sensor using a UV-lamp and electrode stack. We monitored the mass absorption and changes in surface physical properties by monitoring Δ(*f*), which is the change frequency in third harmonics (*f*3), and Δ(*D*), which is the change in dissipation in third harmonics (D3) in QCM-D sensogram. All components of the setup were at 20 °C. The typical QCM-D sensograms of exposure to different vapors are demonstrated in Figure 3 (also see for a complete overview of sensorgrams Supporting Information Table S8–S17 and S19–S24). The average number of at least three experiments with independently synthesized polymer brushes was used to create a map to distinguish the different scents. The mass of absorbed vapor at equilibrium was determined by fitting QCM-D sensograms to the Sauerbrey equation.^68^ We also observed that dissipation Δ(*D*) fluctuations, ranging from 0.1 to 2 ppm, may result from slight flow rate variations during the switch to air with vapor or minor rearrangements of the brush. However, these changes are typically too small to indicate significant structural alterations. This resulted in a fingerprint map of the absorption of different components on the polymer brush-based coatings (Figure 4) (also see Supporting Information Tables S8–S17 and S19–S24).

**Scheme 4:**
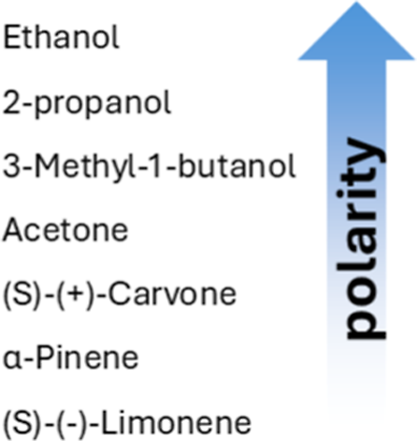
Single-Component VOCs Are Arranged Based on Their Polarity, from Most to Least Polar^69^

The absorbed vapor mass in the different brushes mostly links with the solubility of polymers and monomer units in the corresponding liquids, which can be correlated to their polarity. For example, relatively higher absorption of ethanol vapor on poly(HPMA) and poly(MeOEGMA) is related to the similar polarity character of those polymers to ethanol and their good solubility. The overall affinity of poly(HPMA) and poly(MeOEGMA) brushes for ethanol vapor may be relatively low, as ethanol leaves the brush quickly after exposure to dry air. However, treating the brush surface with ethanol vapor, followed by air drying, likely shifted the equilibrium toward the release of brush-bound components. Despite this, we observed a slow release of α-pinene from the poly(BMA) brush after switching to dry air, as shown in Table S9 of the Supporting Information. This gradual release suggests that α-pinene may have a strong affinity for the brush even after the equilibrium shift. At the same time, higher polarity of poly(CBMA) layers (SWCA 20°) and much lower polarity of poly(BMA) (SWCA 88°) allow a much lower amount of ethanol vapor to absorb (Figure 4). 3-Methyl-butanol absorbs well in poly(MEOEGMA) and poly(BMA) and there is almost no absorption in poly(CBMA) and poly(HPMA). It also should be noted that the polarity and interaction of polymer vapor cannot fully explain the adsorption of different vapors (Scheme 4). Vapor absorption of acetone and nonpolar components such as limonene, carvone, and pinene is evident. Other physical effects can result in changes in interactions between the chains and vapors and thereby contribute to the vapor’s adsorbed mass. Thus, the polarity or solubility aspects cannot fully explain the interaction of the polymer brush with vapor. The overall structure and arrangement of the groups in the polymer brush structure may affect vapor absorption.

The small measured mass of nonpolar vapor on the polar polymer brushes is related to a low interaction potential. For example, adsorption of the α-pinene nonpolar compound on polar poly(HPMA) brush coating (Figure 3c, also see the Supporting Information). Nevertheless, the presence of some extra mass for these polar polymer brushes and components such as limonene, carvone, and pinene can in part be related to their potential adsorption on the surfaces of polymer brushes. The presence of relatively more hydrophobic RAFT-chain end groups and overall more hydrophobic conformation at the air–polymer brush boundary may facilitate this adsorption. In fact, the adsorption closer to surfaces of low interacting vapor on polymer brush was reported in grand canonical molecular dynamics simulations.^36^ The RAFT-chain group’s interactions with terpene vapor are further confirmed by measuring the adsorption of α-pinene (1.2 ppt) on the surfaces of the RAFT-monolayer (see Supporting Information Figure S1). The α-pinene (1.2 ppt) vapor adsorption on the RAFT-monolayer was at 28 ± 5 ng·cm^–2^, while the adsorption of bare silicon oxide surface was at 3 ± 1 ng·cm^–2^.

Next, we exposed the brushes to scents or vapors that contained numerous VOCs. We used vapors with strong interactions with polar polymer brushes, such as an ethanol–water mixture, Jameson whiskey, and Jack Daniels whiskey vapor, and vapor with more nonpolar components, such as lavender and rosemary oil. This experiment allowed us to distinguish different brands of whiskey and water–ethanol mixtures and scents of lavender and rosemary oil by the difference in the absorption patterns of our polymer brush array.

The taste and scent of whiskey are highly dependent on the type of malt used, the distillation process, and the type of wood used in barrels. Thus, those processes change the chemical composition and the amount of the different compounds in whiskey and, thereby, its scent. Despite the whiskey and water–ethanol mixture having the same ethanol volume percentage of 40%, the adsorbed masses on the polymer brushes differ. This is likely caused by the different contents of phenolic, ester, and aldehyde compounds, since the adsorption differences are more prominent on more polar brushes such as poly(HPMA).

We also observed different patterns of adsorption of rosemary and lavender oil. Both scents have numerous VOCs in their composition. Lavender oil contains more than 100 chemicals, with linalool and linalyl acetate being the two most prominent. Rosemary oil contains as well numerous components such α-pinene, 1,8 cineole, and camphene as well as ketones, including camphor, alcohol borneol, carnosic acid, carnosol, and rosmarinic acid. Even though we see some correlation with the absorption of single components, such as α-pinene, it is difficult to estimate what components in the vapor mixture contribute to the total absorbed mass on the polymer brush. It is a combined effect of all of the components in a complex vapor mixture that determines the total adsorbed mass on the polymer brushes. However, for scent distinction, it is not necessary to know the contribution of each component. One only needs to be able to distinguish the fingerprints, similar to that of the mammalian olfactory system.

**Figure 5:**
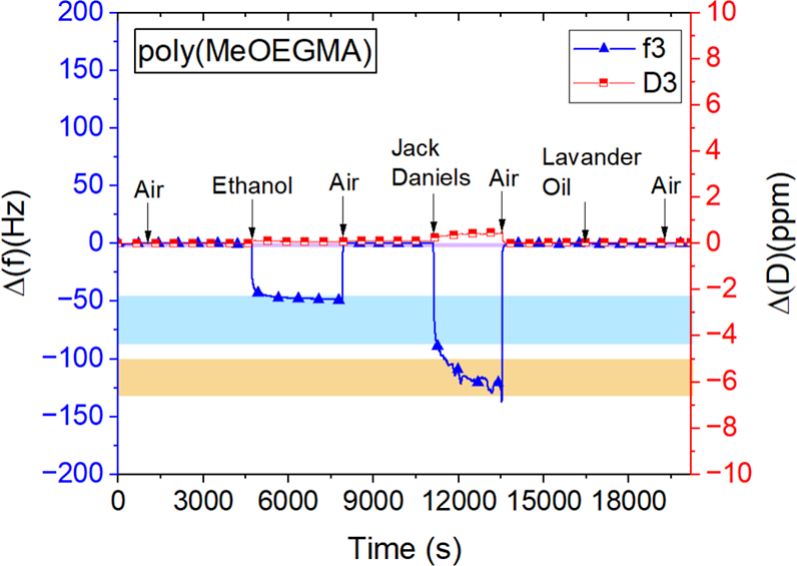
Representative sensorgram of reusability of polymer brush poly(MeOEGMA) coatings exposed to the vapor of ethanol, Jack Daniels whiskey, and lavender oil. The error margins for absorption of corresponding vapors separately on poly(MeOEGMA) brush are shown with blue (ethanol), brown (Jack Daniels whiskey), and purple (lavender oil). Please also see Supporting Information Table S18 for a full overview of all sensorgrams on reusability of different polymer brushes.

Next, the reusability of the prepared brushes was explored. Brushes were exposed to different scents and alternated by periods of dry air. The levels of absorption in and adsorption onto the polymer brushes remained within the error margins observed for exposure of a single component or scent Figure 5 (also see Supporting Information Table S18). Furthermore, we observed reproducible results when exposing the polymer brushes to different vapors and dry air for up to six cycles (Supporting Information Table S18). Thus, the brush coatings have high recycling potential in future sensors.

**Figure 6:**
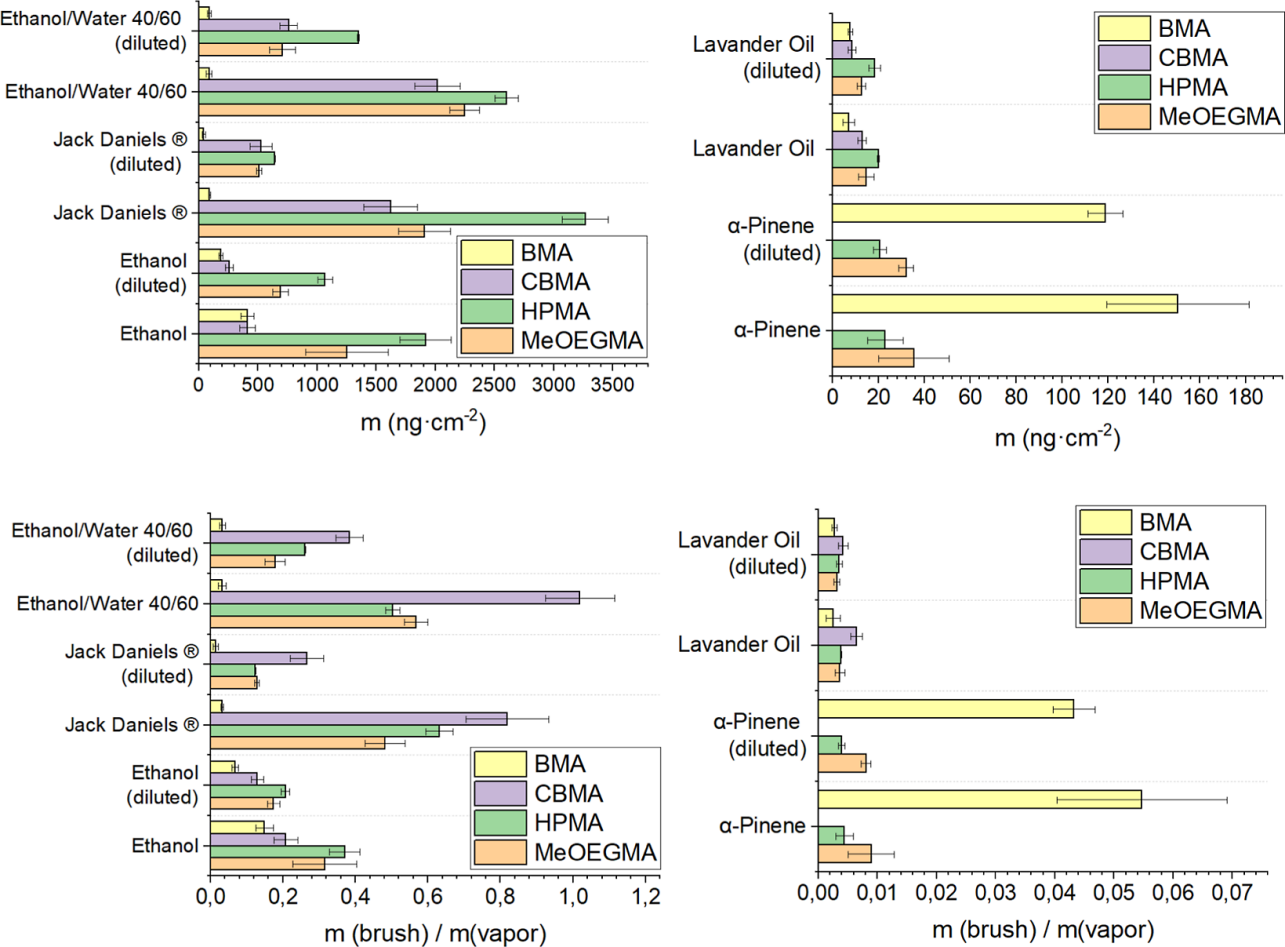
Adsorbed mass and ratio between absorbed mass and mass of the polymer brush coating of different vapor and vapor diluted with dry air (∼80% of initial concentration), ethanol(12 ppt) α-pinene(1.2 ppt) ethanol (diluted) (10 ppt) α-pinene (diluted) (0.9 ppt) on the surfaces of poly(BMA), poly(CBMA), poly(HPMA), and poly(MeOEGMA) brushes.

We further look at patterns or finger prints of absorption of different VOCs and scents in polymer brushes at different vapor concentrations. We achieved it with a dilution of vapor with dry air. We have measured the absorption of ethanol at 12 and 10 ppt (ethanol diluted) and α-pinene at 1.2 and 0.9 ppt (α-pinene diluted). In a similar manner we diluted the vapor of complex scents of ethanol–water mixture vapor, Jack Daniels whiskey, and lavender oil approximately to 80% and also measured absorption in our four polymer brush coatings (Figure 6). The ratio of the absorbed mass of ethanol (12 ppt) between poly(MeEOGMA): poly(HPMA): poly(CBMA): poly(BMA) coatings was 3.0 ± 0.9:4.7 ± 0.5:1.0 ± 0.2:1.0 ± 0.1 and for ethanol (10 ppt) (diluted) 3.7 ± 0.3:5.7 ± 0.3:1.4 ± 0.2:1.0 ± 0.4. Similarly good correlation in ratios of adsorbed mass was observed for α-pinene (1.2 ppt): 2.0 ± 0.8:1.3 ± 0.4:0.0 ± 0.0:8.5 ± 1.8 and α-pinene diluted (0.9 ppt) 1.8 ± 0.2:1.2 ± 0.2:0.0 ± 0.0:6.7 ± 0.4 (Figure 6, and please also see Supporting Information Tables S26 and S27). The overall drop in the signal of QCM-D was observed to be α-pinene 86 ± 14%, lavender oil 90 ± 14%, and ethanol 55 ± 14%, correlating with expected dilutions of 80%. This further confirms that for a single VOC vapor, we can effectively fingerprint different vapors even with only four chemically different vapors.

We have not observed a good correlation between absorbed mass on different brushes in the diluted and undiluted vapor of complex mixtures of a water–ethanol mixture and Jack Daniels whiskey. This may be related to additional effects such as collaborative and competitive absorption.^37^ Nevertheless, the absorption patterns were different from each other at both concentrations. Thus, those vapors can still be distinguished. The nonperseverance of ratios of adsorbed mass could be related to the strong affinity of brushes to one of the components in the vapor medium, such as water, or changes in the properties of the brush at certain vapor concentrations in the process of swelling. These aspects and water vapor’s effect on other components’ absorption will be further investigated in future studies. The ratios of adsorbed mass in the case of lavender oil and diluted lavender oil vapor are well correlated. This is probably related to the fact that most vapor is adsorbed close to the surface of the brush.

The SI-PET-RAFT technique has previously enabled the coating of various surfaces with polymer brushes, ranging from gold^44^ and silicon^65,66^ to PCL nanofibers.^70^ Additionally, the range of monomers polymerized using this method is expanding exponentially and is not limited by potential interactions with metal catalysts, as seen in SI-ATRP techniques.^40^ Therefore, SI-PET-RAFT’s high versatility and relative simplicity facilitate the straightforward adaptation of this technology to diverse sensor platforms and the effortless expansion of the library of chemically distinct polymer brushes.

## Conclusions

In this work, we developed an array of polymer brushes composed of carboxybetaine methacrylamide (CBMA), *N*-(2-hydroxypropyl) methacrylamide (HPMA), oligo(ethylene glycol) methacrylate (MeOEGMA), and butyl methacrylate (BMA). The polymerization was carried out using the oxygen-tolerant, metal-free surface-initiated photoiniferter reversible addition-fragmentation chain transfer (SI-PET-RAFT) technique. Those brushes cover properties from hydrophilic to hydrophobic, thus providing a wide interaction window with vapor containing polar and nonpolar components and their mixtures. The polymer brush array was exposed to different single-compound VOCs such as acetone, limonene, ethanol, 2-propanol, 3 methyl 1-butanol, carvone, and α-pinene as well as scents that consist of numerous VOCs such as the scent of the water–ethanol mixtures, rosemary oil, lavender oil, and two brands of whiskey, Jack Daniels and Jameson. The pattern of mass absorption of different polymer brushes allowed us to distinguish different single-component scents and multiple-component scents. Moreover, we demonstrated the reusability of created coatings by exposing the polymer brush layer to different vapors with a dry air break. We believe that the SI-PET-RAFT technique, with its high versatility and surface independence for creating polymer brush coatings, represents a significant first step toward developing affordable scent sensors. This proof-of-concept further lays the groundwork for advanced sensor applications in detecting scents and volatile organic compounds (VOCs).

## Supporting Information Available

Materials and methods, XPS characterization, DFT calculations, AFM topography, and QCM-D measurement (PDF)
