# Supplementary material for: Exploring Scent Distinction with Polymer Brush Arrays
Source: ACS Appl Polym Mater. 2025 Mar 7;7(6):3842–52. doi: 10.1021/acsapm.5c00066 (PMC11959526; doi:10.1021/acsapm.5c00066)
Supplement: Supplementary file 1 — ap5c00066_si_001.pdf [file ap5c00066_si_001.pdf]

Supporting information for

Exploring Scent Distinction with Polymer Brushes Arrays

Andriy R. Kuzmyn[1]*, Ivar Stokvisch[1], Gerrit-Jan Linker[2], Jos Paulusse[1] and Sissi de Beer[1]*

1) Department of Molecules & Materials, MESA+ Institute, University of Twente, 7500AE Enschede, The Netherlands; 2) MESA+ Institute for Nanotechnology, University of Twente, 7522 NB Enschede, The Netherlands.

Table of content

Materials and methods	S2
XPS characterization	S5
DFT calculations	S7
AFM topography	S9
QCM-D measurements	S11
References	S89

Corresponding authors

Email: a.r.kuzmyn@utwente.nl , andriy.kuzmyn@gmail.com, s.j.a.debeer@utwente.nl

Materials and methods

Materials. All chemical reagents were used without further purification unless otherwise specified. 4-Cyano-4-(phenylcarbonothioylthio)pentanoic acid *N*-succinimidyl ester (RAFT-NHS), oligo(ethylene glycol) methyl ether methacrylate (average M_n 300) (MeOEGMA), butyl methacrylate (BMA), triethanolamine (TEOA) ethanol (EtOH) (99.9%), dimethyl sulfoxide (DMSO), acetone (99.5%), rosemary oil, (S)-(-)-Limonene, α-Pinene, (S)-(+)-Carvone, 2-propanol, 3-Methyl-1-butanol, lavender oil and dry tetrahydrofuran (THF, 99.9%), were purchased from Sigma-Aldrich. N-(2-Hydroxypropyl) methacrylamide (HPMA), carboxybetaine methacrylamide (CBMA) was obtained from Polysciences. Inc. Eosin Y was acquired from TCI Europe. Deionized water was produced with a Milli-Q integral 3 system Millipore, Molsheim, France (Milli-Q water). Jameson whiskey and Jack Denials whiskey were purchased at Jumbo Supermarketen. Quartz crystal microbalance chips were acquired from Quantum Design GmbH. 100 ppm Isobutylene in air calibration gas was acquired at Hartwig instruments BV.

Light Source. LEDs with a maximum intensity at 410 nm (Intelligent LED Solutions, product number: ILH-XO01-S410-SC211-WIR200) were used, and the current was set at 700 mA, corresponding to a total radiometric power of 2.9 W, according to manufacturer specifications. The light intensity of the halogen lamp was measured to be 3.5 $\mu W \cdot cm^{-2}$.

Formation of RAFT agent-functionalized monolayers. The RAFT-agent immobilization was conducted by previously published procedures.[1-4]

SI-PET-RAFT synthesis of polymer brushes. The polymerization was conducted according to a modification of a previously reported procedure.[1-5] A dye stock solution with photocatalyst was prepared to contain photocatalyst (25mg, 39 µmol) and TEOA (160 mg, 1.60 mmol) in 10 mL of Milli-Q water in case of BMA polymerization in catalyst solution was dissolved in dimethyl sulfoxide (DMSO).

In a typical procedure, the monomer MeOEGMA (94 mg, 0.30 mmol) or CBMA (76 mg, 0.30 mmol), or HPMA (190 mg, 1.30 mmol), or BMA (43 mg, 0.30 mmol) was dissolved in Milli-Q water (1 mL) or DMSO (for BMA), and subsequently, 10 µL of the stock solution was added. The mixture was vortexed and added to vials containing surfaces with an immobilized RAFT agent so that the liquid formed a thin layer (ca. 2 mm) on top of the surfaces. Immediately after this, the polymerization was conducted by irradiating the vials with visible light from an LED light source for different periods of time. The light source was placed 3–4 cm from the substrates in these experiments. The polymerization was stopped by switching off the light source. The samples were removed from the solution, rinsed with Milli-Q water-ethanol, and blown dry under a stream of argon. In the case of BMA polymerization, the surfaces were washed first with DMSO, followed with acetone ethanol, and finally with Milli-Q water.

X-ray photoelectron spectroscopy (XPS). XPS measurements were performed using a PHI Quantes Dual Scanning X-ray Photoelectron Microprobe (Japan). All samples were analyzed using a focused monochromated Al Kα X-ray source (spot size of 100 µm) at a constant dwelling time 50 ms and pass energy: wide-scan 280 eV narrow-scan: 112 eV. The power of the X-ray source was 25 W (15 kV). Charge compensation was applied during the XPS scans. XPS wide-scan and narrow-scan spectra were obtained under ultra-high vacuum conditions (base pressure $1.0 \cdot 10^{-6}$ Pa). All narrow-range spectra were corrected with a linear background before fitting. The spectra were fitted with symmetrical Gaussian/Lorentzian (GL(30)) line shapes using CasaXPS.[6] All spectra were referenced to the C1s peak attributed to C–C and C–H atoms at 285.0 eV.

Computational details of DFT calculations. The ΔSCF approach[7] was used to calculate carbon 1s electron binding energies using density functional theory (DFT) with the PB86[8, 9] GGA functional, the TZ2P[10] basis set using a small frozen core. Scalar relativistic effects were accounted for. The Amsterdam Modeling Suite (AMS)[11] was used. After geometry optimization for the neutral molecule, the frozen core approximation is removed only for the targeted carbon atom for which the 1s-orbital population is reduced to one. A geometrical global minimum for the neutral molecule was ensured by performing an IR frequency calculation.

To assist XPS peak assignment, spectra were simulated based on calculated binding energies and using the count of equivalent carbon atoms as intensities. A Gaussian broadening is applied.

Ellipsometry. The polymerization kinetics of poly(BMA) brushes and the thickness of other brushes were determined using a Woollam M-2000X (USA) variable-angle spectroscopic ellipsometer (VASE). The ellipsometric data were acquired in the air at room temperature using light in the wavelength range of λ = 300 – 1000 nm at 65°, 70°, and 75 angles of incidence. The ellipsometric data were fitted using the CompleteEASE software to a model composed of a Si substrate, a 2 nm native oxide layer, and a Cauchy layer for the polymer brush. This topmost layer's thickness was fitted using the Cauchy model, and optical constants A = 1.450 and B = 0.01 parameters.

Atomic force microscopy (AFM). AFM surface topography images were acquired by a Bruker Dimension iCon AFM (Oxford Instruments, United Kingdom). The instrument was operated in tapping mode and equipped with a silicon cantilever (AC240TS-R3, k = 2 N/m) with a nominal tip radius of ~7 nm. Gwyddion[12] software was used to process and analyze the AFM topography images.

SWCA Measurements. The wettability of the modified surfaces was determined by automated static water contact angle measurements using a Krüss DSA 30S goniometer. The volume of a drop of demineralized water was 3 µL. Contact angles from sessile drops measured by the tangent method were estimated using a standard error propagation technique involving partial derivatives. The surface energy was determined by spontaneously depositing water and iodomethane droplets 2µL and fitting according to the Owens, Wendt, Rabel, and Kaelble model (OWRK).

Quartz Crystal Microbalance with Dissipation Monitoring. QCM-D was utilized to assess mass variations along with the viscoelastic and structural characteristics of the different films. The measurements were performed by using silicon-coated quartz resonators (AT-cut, Biolin Scientific, Sweden) with a fundamental frequency (f_0) of 5 MHz in a QCM-D set-up (Q-Sense E4, Biolin Scientific, Sweden) at 20 °C. Before each experiment, the dry air was pumped at a flow rate of 7 L·min^{-1} for at least 10 min for a stable baseline. Following the injection of the vapor of different solutions. The vapors were produced by bubbling the corresponding solution with dry air with the same flow rate as the baseline (7 L·min^{-1}). The concentration of single vapor components was determined by, and this sensor Ion Science MiniPID2 PPM WR & SDK sensor (ION Science Ltd) was calibrated by isobutylene. Typically, the vapor concentration reaches stable numbers within the first 2-3 min of bubbling. In a typical experiment, dry air flows over the QCM-D sensor for 10 minutes following vapor injection. The vapor is then pumped over the surface of the QCM-D chip for 60 minutes to achieve equilibrium. After that, dry air is injected over the QCM-D chip and returns to the baseline.

QSoft, DFind, and Origin software were used to record and process the data. Frequency (Δf) and dissipation shifts (ΔD) were acquired in real-time at the 3rd (15 MHz), 5th (25 MHz), 7th (35 MHz), 9th (45 MHz), and 11th (55 MHz) harmonic overtones. The third (15 MHz) overtones were reported to compare different polymer coatings. A 5 MHz crystal will have a mass sensitivity of 17.7 ng/(cm^2·Hz), and a 10 MHz crystal will have a theoretical mass sensitivity of 4.4 ng/(cm^2·Hz). The change in frequency

of the quartz crystal can be associated with a change in mass after the implementation of a film, resulting in the Sauerbrey equation (Equation (1))[13], where Δm is the change in a real mass density of the film, Δf_n is the frequency shift, n is the harmonic number, and c (17.7 ng·cm^{-2}·Hz^{-1}) is the mass sensitivity constant:

$$\Delta m = \frac{c}{n}\Delta f_n \quad (1)$$

XPS characterization

The XPS wide-scan spectrum of a poly(CBMA) layer with a thickness of 20 nm, as determined by ellipsometry, showed three prominent peaks for O1s, N1s, and C1s in a ratio of 6.7:1.0:1.2 (See supporting information Table S1). The ratio between the peaks correlates roughly with what is theoretically expected for CBMA (6.0 : 1.0 : 1.5). The XPS narrow-scan spectrum of the C1s region shows three main peaks of carbon atoms: [C−C/H] (285.0 eV), [C−N] (286.2 eV), and [C=O] (287.8 eV) in a ratio of 2.8 : 2.4 : 1.0 (Figure 2d). The C1s binding energies we computed correspond to peak positions [C−C/H] (285.3 eV),[C−N] (286.5 eV), and [C=O] (287.0 eV) with an estimated peak ratio of 2:2:1 (Figure 2h) (also see Supporting information Table S6).

The XPS survey spectrum of a poly(BMA) layer with a thickness of 25 nm has two main peaks, C1s and O1s, in the ratio 4.8 : 1.0 (See supporting information Table S1). This ratio correlates with the elemental composition of BMA 4.0 : 1.0. We further confirmed the composition of poly(BMA) brushes using narrow C1s XPS spectra (Figure 2a). There are three peaks in C1s spectra attributed to [C−C/H] (285.0 eV), [C−O] (286.6 eV), and [C−O] (288.8 eV) in a ratio 5.8 : 1.1 : 1.0. This is supported by our estimated ratio 6 : 1 : 1 and calculated peak positions [C−C/H] (285.1 eV), [C−O] (286.5 eV), and [C−O] (288.3 eV) (Figure 2e) (also see Supporting information Table S5).

The chemical composition of poly(HPMA) brushes was also confirmed by XPS. The XPS wide-scan spectrum of poly(HPMA) brushes with an ellipsometry thickness of 44 nm (Figure 2b) shows three main peaks related to O1s, N1s, and C1s electrons in a 1.8 : 1.0 : 7.8 ratio (See supporting information Table S1), which is in agreement with the elemental composition of the poly(HPMA) structure (2:1:7). The narrow-scan XPS C1s spectrum (Figure 2f) displays four prominent peaks [C−C/H] (285.0 eV),[C−N] (286.1 eV), [C-O] (287.0eV) [C=O] (288.0 eV) in fitted ratio 4.7 : 1.3 : 1.0 : 1.0 (Figure2b). This fitting also aligns with simulated XPS spectra for HPMA and the expected peak ratio 4 : 1 : 1 : 1 and peak positions [C−C/H] (285.1 eV),[C−N] (286.0 eV), [C-O] (286.5 eV) [C=O] (287.5 eV) (Figure 2f) (also see Supporting information Table S2).

The wide-scan XPS spectra of poly(MeOEGMA) showed two main O1s and C1s peaks in ratio 2.7 : 1.0 (See supporting information Table S1). The ratio correlates with what was previously reported for these brushes. The narrow C1s poly(MeOEGMA) XPS spectra revealed three peaks [C−C/H] (285.0 eV), [C−O] (286.5 eV), and [C−O] (288.9 eV) in ratio 1.0 : 9.4 : 2.9. This fitting also aligns with simulated XPS spectra for a combination of monomers of MeOEGMA (Mw = 278) and MeOEGMA (Mw = 322) with predicted binding energies and ratios : [C−C/H] (285.1 eV), [C−O] (286.4 eV), and [C−O] (287.9 eV) in ratio 1 : 10 : 3 (Figure 2c) (also see Supporting information Table S3 and S4).

Table S1. XPS characterization of poly(HPMA), poly(BMA), poly(MeOEGMA), poly(CBMA) brushes obtained by SI-PET-RAFT wide XPS spectra

<table>
<tr><th>Surface</th><th>Wide XPS spectra</th></tr>
<tr><td>Poly(HPMA)</td><td>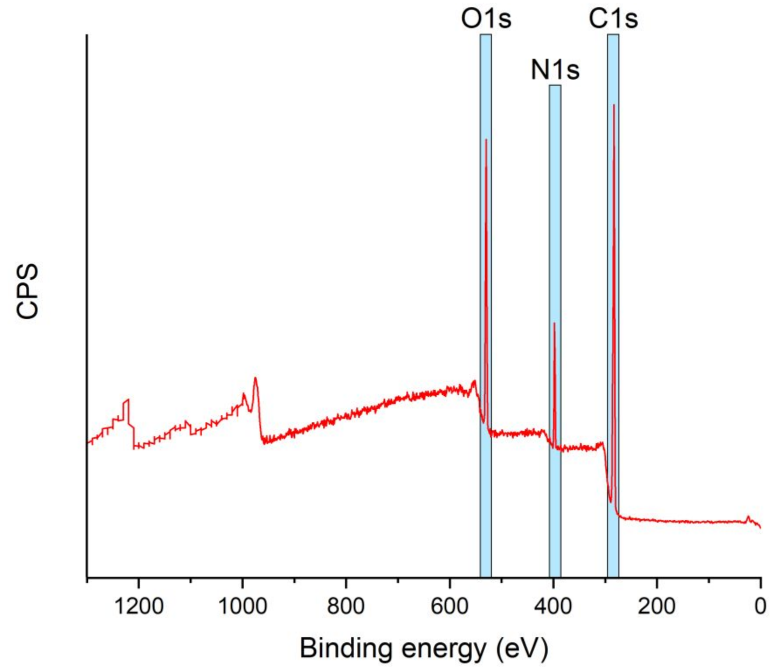
</td></tr>
<tr><td>Poly(CBMA)</td><td>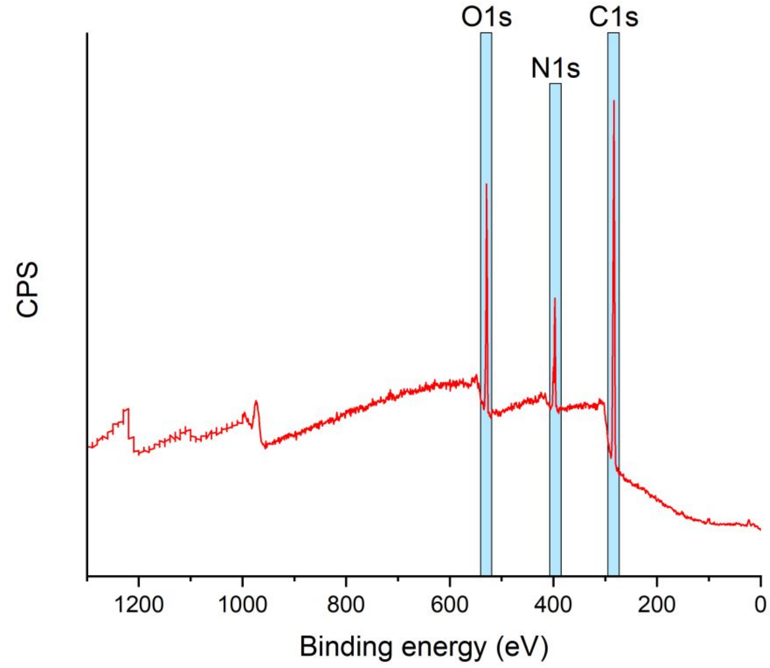
</td></tr>
</table>

Poly(MeOEGMA)	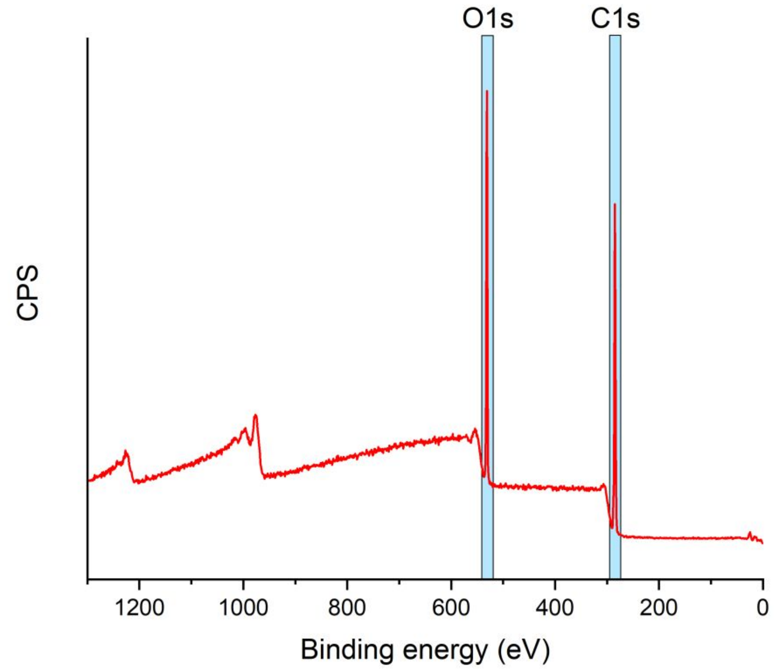
Poly(BMA)	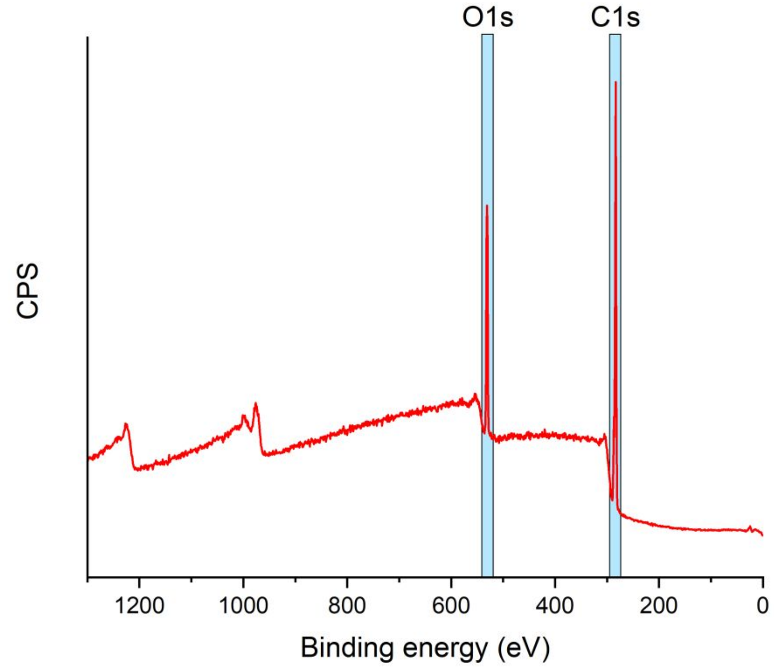

DFT calculations

Table S2. DFT calculations for HPMA

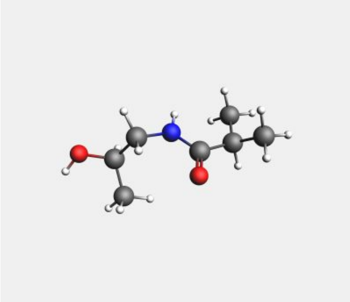

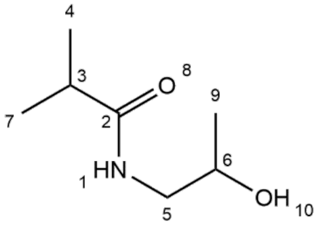

Carbon	Shift (eV)
C2	2.5
C3	0.4
C4	0.3
C5	1.0
C6	1.6
C7	0.0
C9	0.1

Table S3. DFT calculations for MeOEGMA (M_w 278)

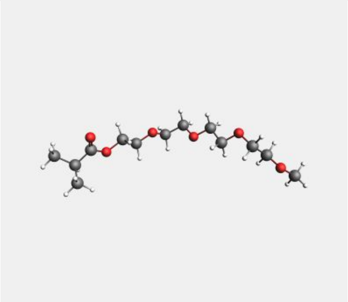

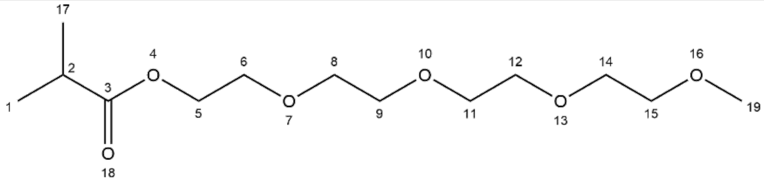

Carbon	Shift (eV)
C1	0.0
C2	0.4
C3	2.8
C5	1.4
C6	1.4
C8	1.4
C9	1.4
C11	1.3
C12	1.3
C14	1.3
C15	1.3
C17	0.1
C19	1.5

Table S4. DFT calculations for MeOEGMA (M_w 322)

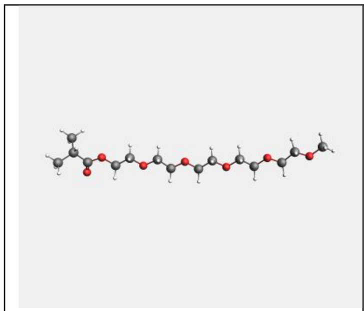

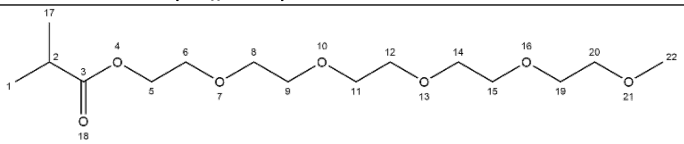

Carbon	Shift (eV)
C1	0.0
C2	0.5
C3	2.8
C5	1.5
C6	1.5
C8	1.4
C9	1.4
C11	1.3
C12	1.3
C14	1.3
C15	1.3
C17	0.1
C19	1.4
C20	1.4
C22	1.6

Table S5. DFT calculations for BMA

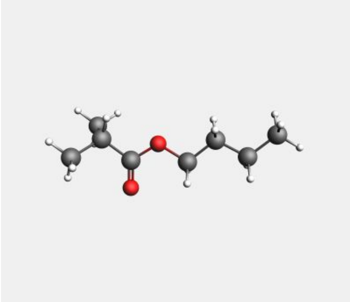

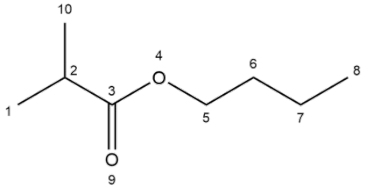

Carbon	Shift (eV)
C1	0.0
C2	0.2
C3	3.3
C5	1.4
C6	0.2
C7	0.2
C8	0.2
C10	0.1

Table S6. DFT calculations for CBMA

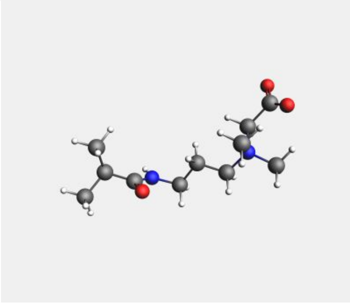

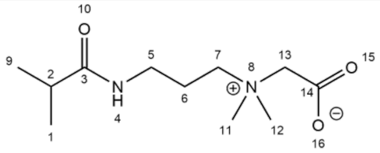

Carbon	Shift (eV)
C1	0.0
C2	0.4
C3	2.5
C5	1.5
C6	0.6
C7	1.7
C11	1.3
C12	1.3
C13	0.6
C14	1.6

AFM topography

Table S7. Representative AFM topography images of polymer brush-coated surfaces.

Surface	Representative AFM topography images
Poly(HPMA)	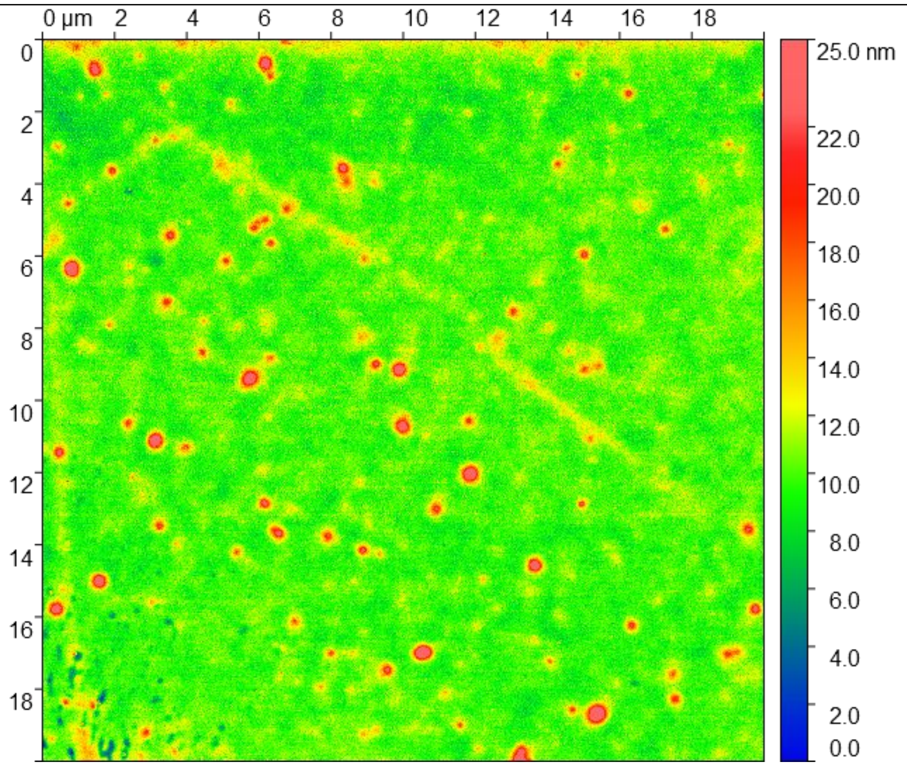
Poly(CBMA)	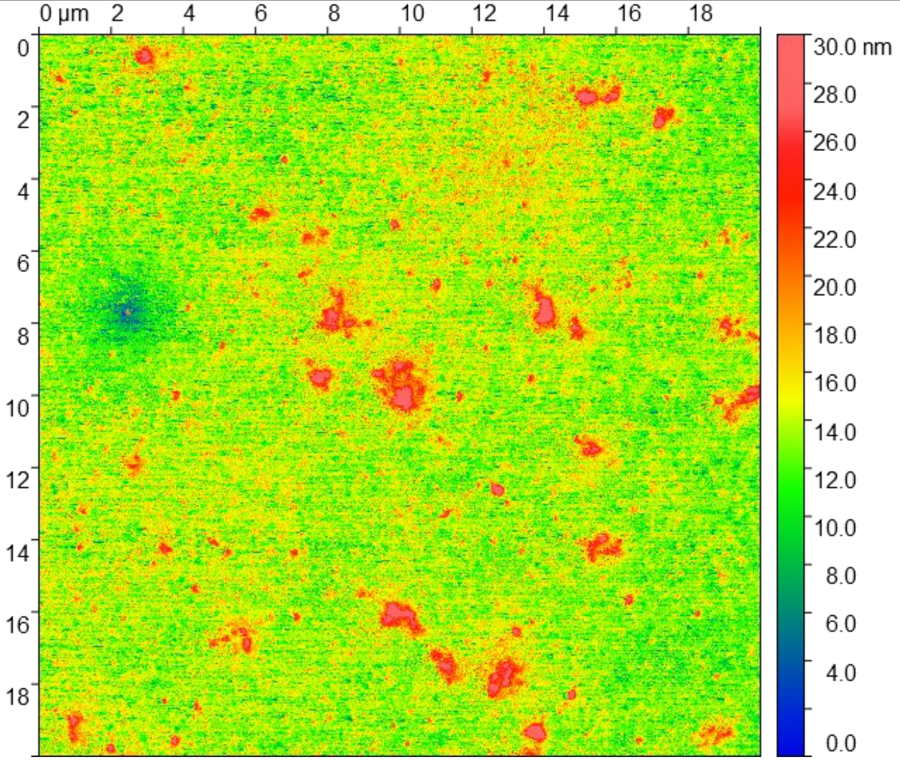

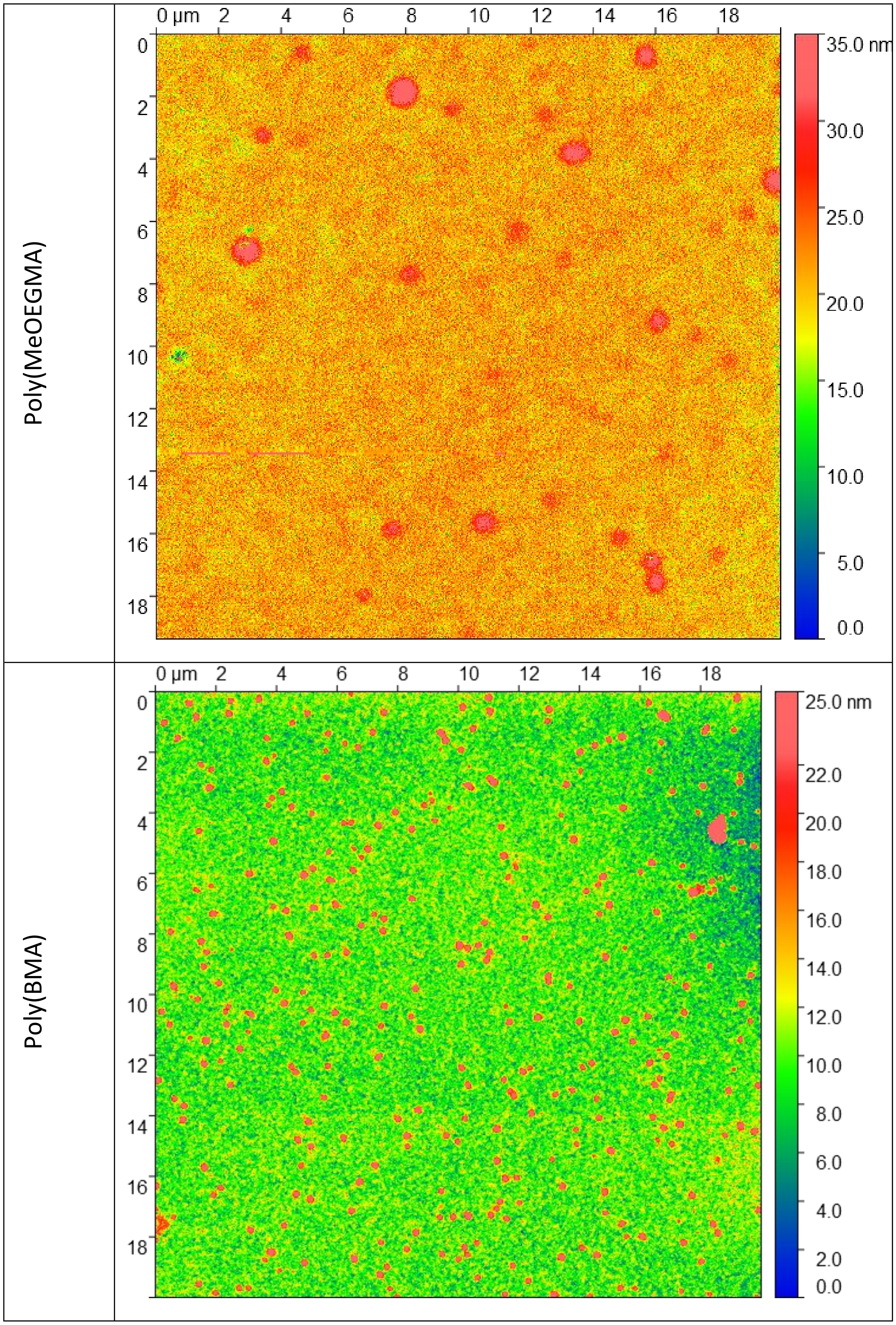
Poly(MeOEGMA)
0 μm 2 4 6 8 10 12 14 16 18
35.0 nm
30.0
25.0
20.0
15.0
10.0
5.0
0.0
Poly(BMA)
0 μm 2 4 6 8 10 12 14 16 18
25.0 nm
22.0
20.0
18.0
16.0
14.0
12.0
10.0
8.0
6.0
4.0
2.0
0.0

QCM-D measurements

Table S8. Representative QCM-D sensograms of exposure of different polymer brush and bare silicon oxide surface (SiO_2) coatings to ethanol (12 ppt) vapor.

**Surface**	**Representative QCM-D sensograms**
Poly(HPMA)	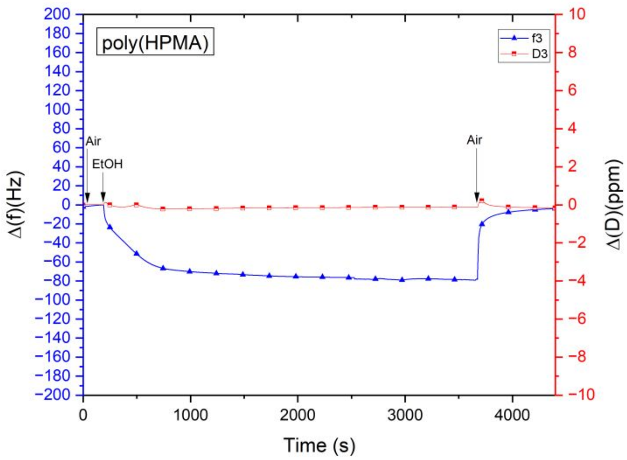<br>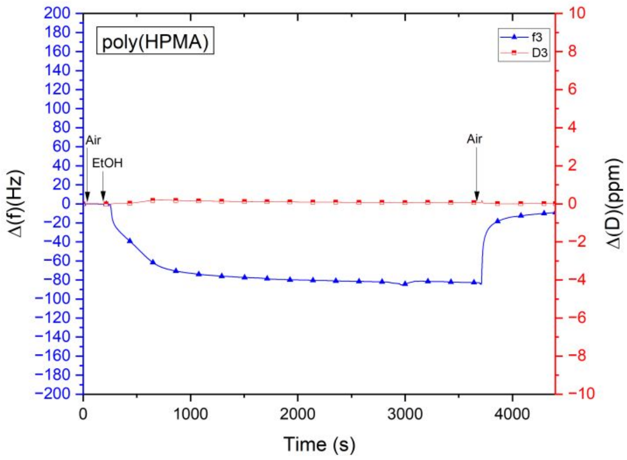

	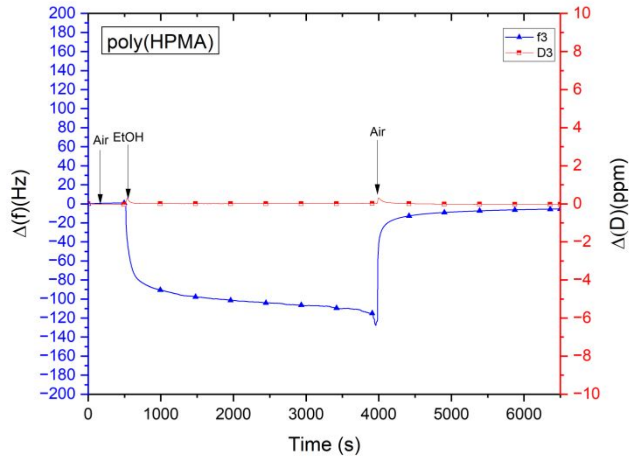poly(HPMA)<br>f3<br>D3<br>Air<br>EtOH<br>Air<br>Δ(f)(Hz)<br>Δ(D)(ppm)<br>Time (s)

Poly(CBMA)	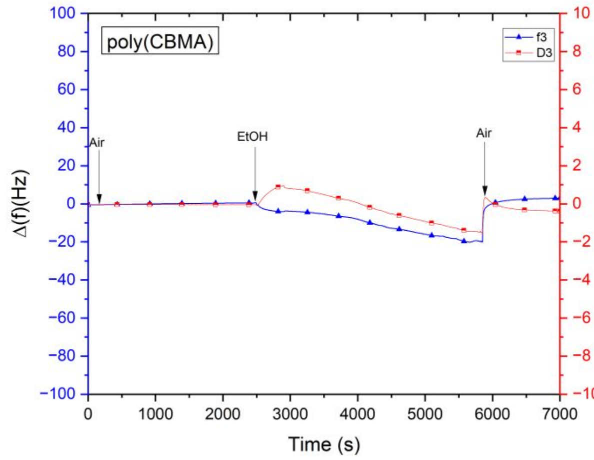poly(CBMA)<br>f3<br>D3<br>Air<br>EtOH<br>Air<br>Δ(f)(Hz)<br>Δ(D)(ppm)<br>Time (s)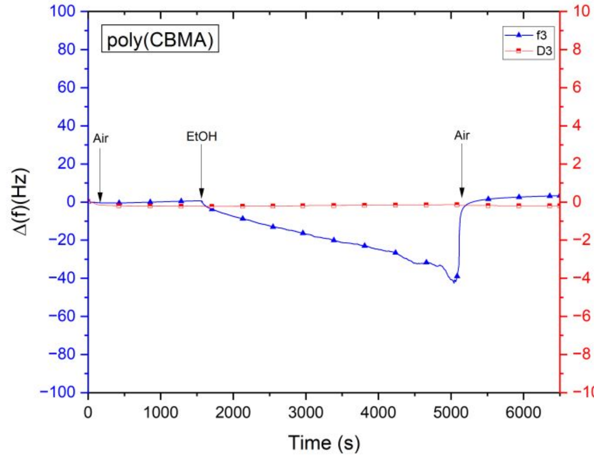poly(CBMA)<br>f3<br>D3<br>Air<br>EtOH<br>Air<br>Δ(f)(Hz)<br>Δ(D)(ppm)<br>Time (s)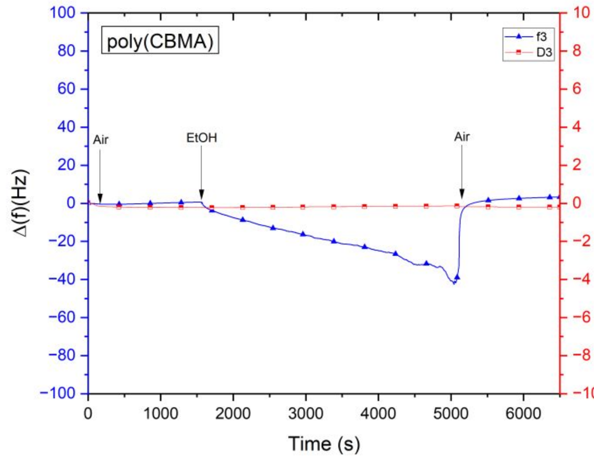poly(CBMA)<br>f3<br>D3<br>Air<br>EtOH<br>Air<br>Δ(f)(Hz)<br>Δ(D)(ppm)<br>Time (s)

Poly(MeOEGMA)

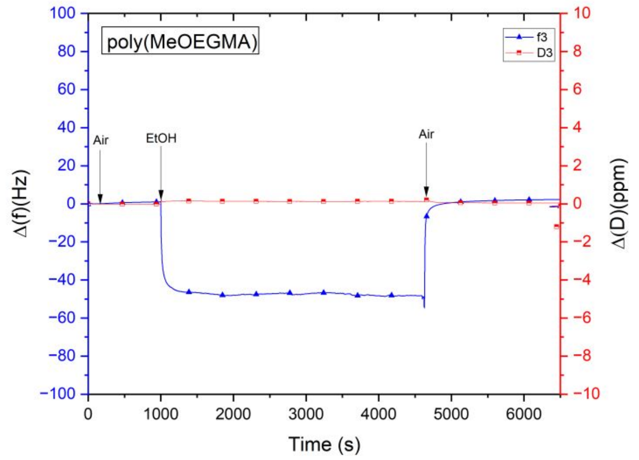

Poly(BMA)

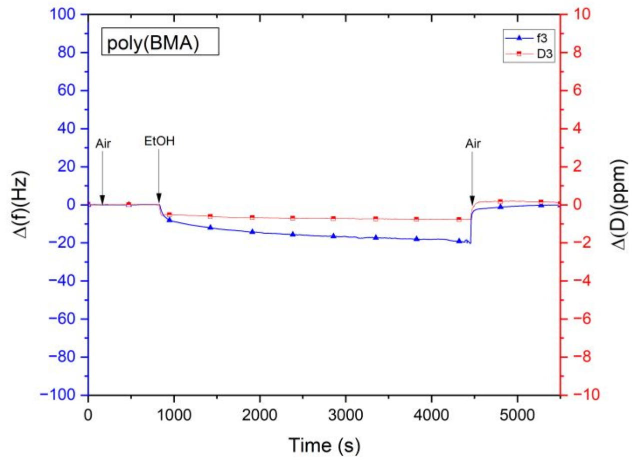

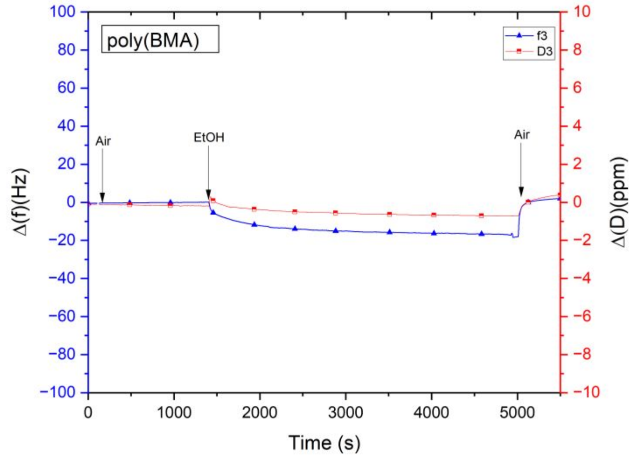

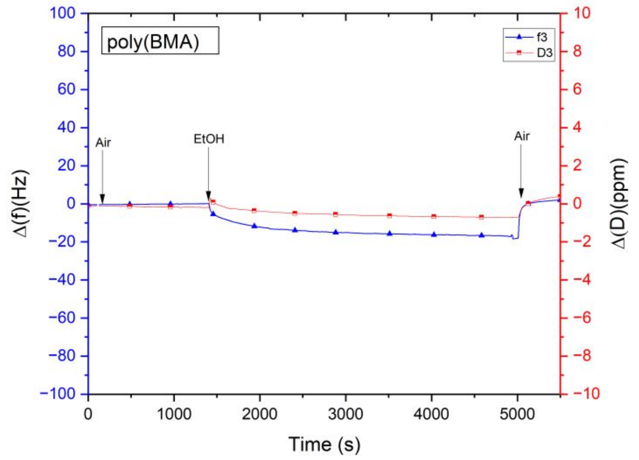
poly(BMA)
f3
D3
Air
EtOH
Air
Δ(f)(Hz)
Δ(D)(ppm)
Time (s)

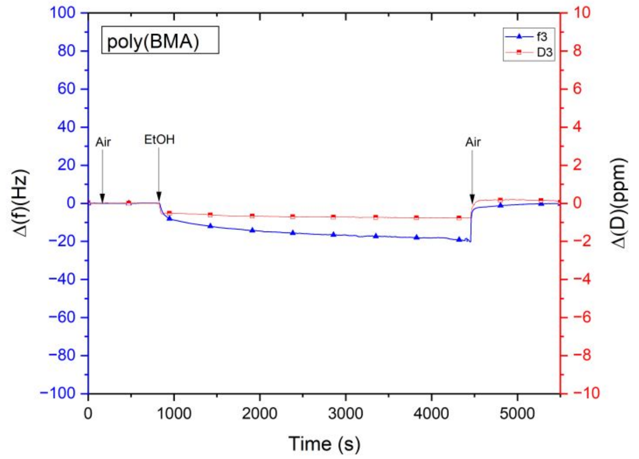
poly(BMA)
f3
D3
Air
EtOH
Air
Δ(f)(Hz)
Δ(D)(ppm)
Time (s)

SiO_2

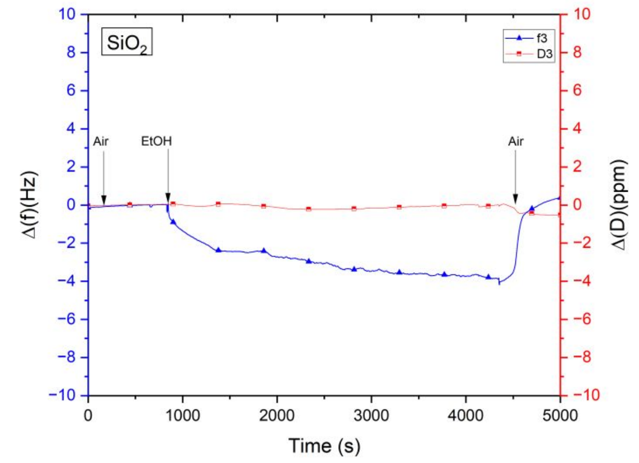
SiO2
f3
D3
Air
EtOH
Air
Δ(f)(Hz)
Δ(D)(ppm)
Time (s)

Table S9. Representative QCM-D sensograms of exposure of different polymer brush and bare silicon oxide surface (SiO_2) coatings to α-pinene (1 ppt) vapor.

Surface	Representative QCM-D sensograms
Poly(HPMA)	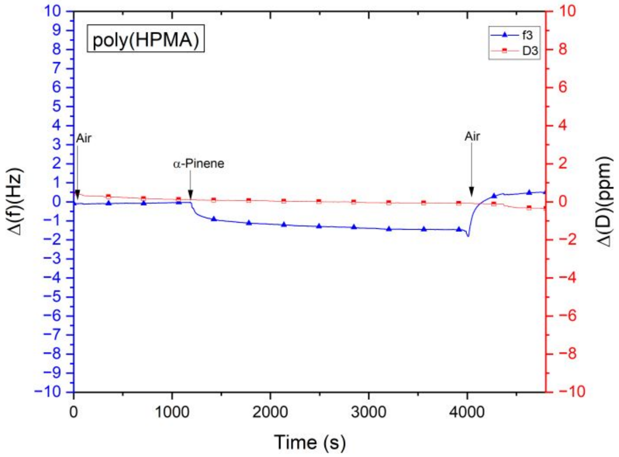<br>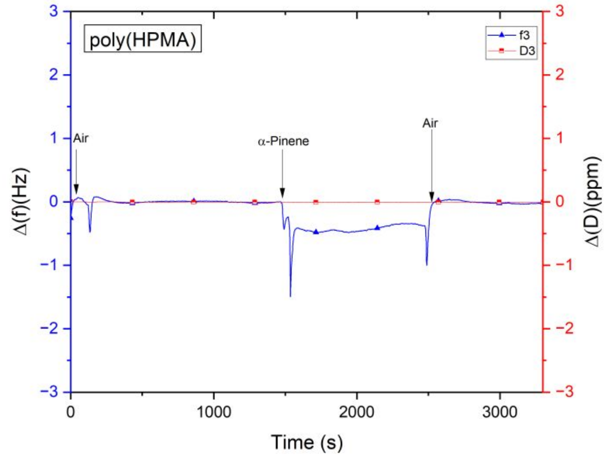<br>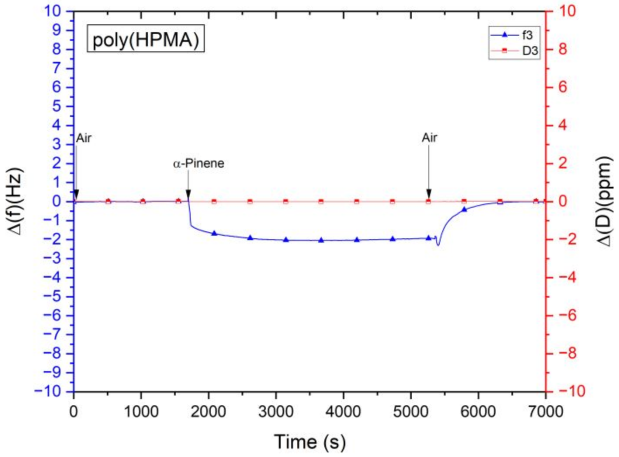

Poly(CBMA)	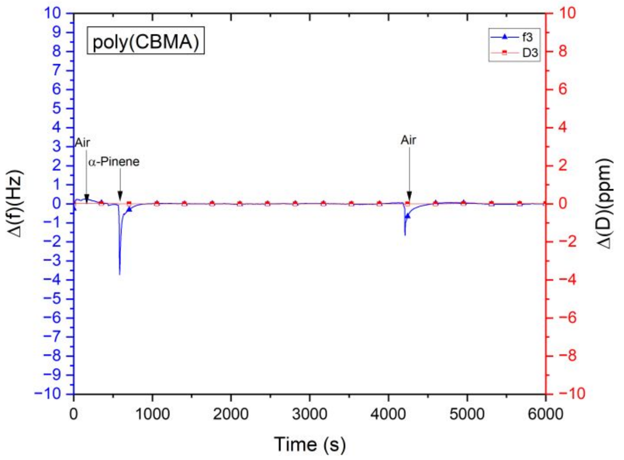
Poly(MeOEGMA)	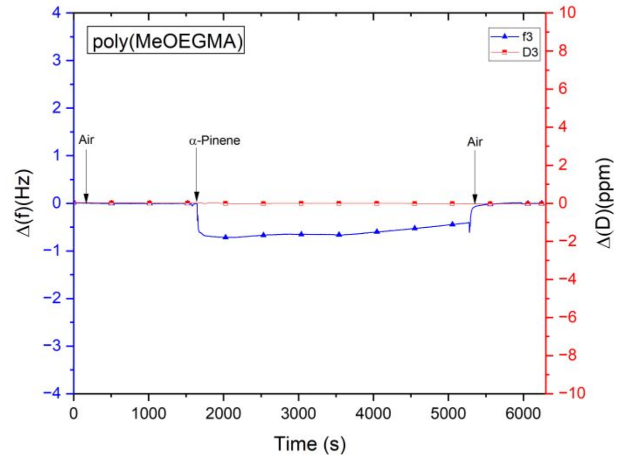<br>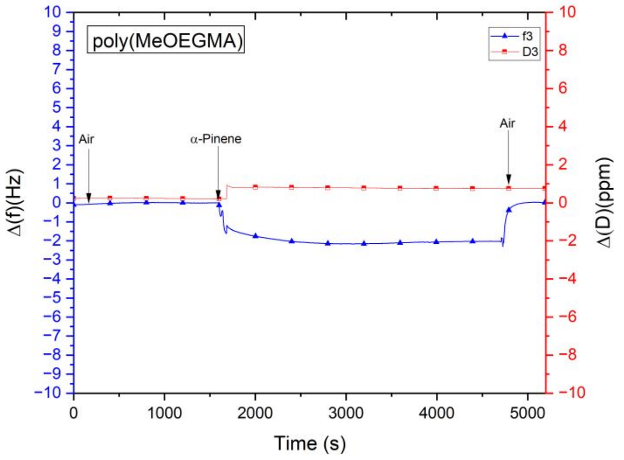

	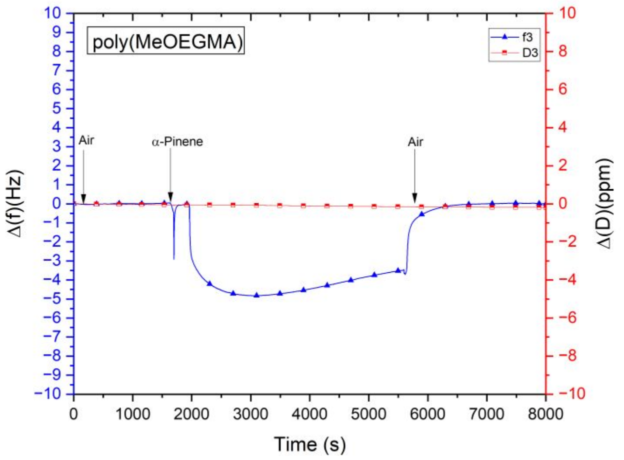
Poly(BMA)	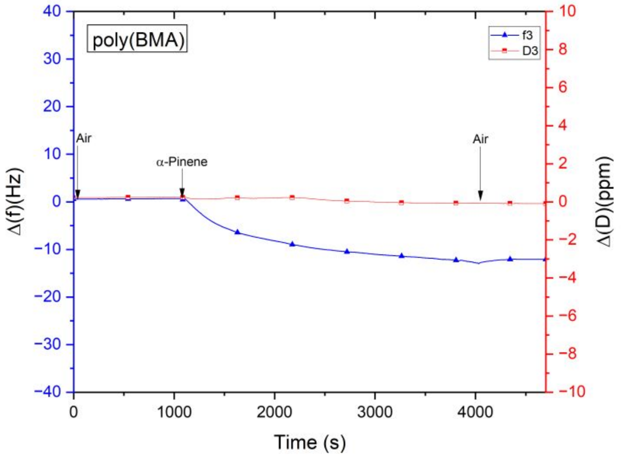

poly(MeOEGMA)
f3
D3
Air
α-Pinene
Air
Δ(f)(Hz)
Δ(D)(ppm)
Time (s)

poly(BMA)
f3
D3
Air
α-Pinene
Air
Δ(f)(Hz)
Δ(D)(ppm)
Time (s)

<table>
<tr>
<td></td>
<td>
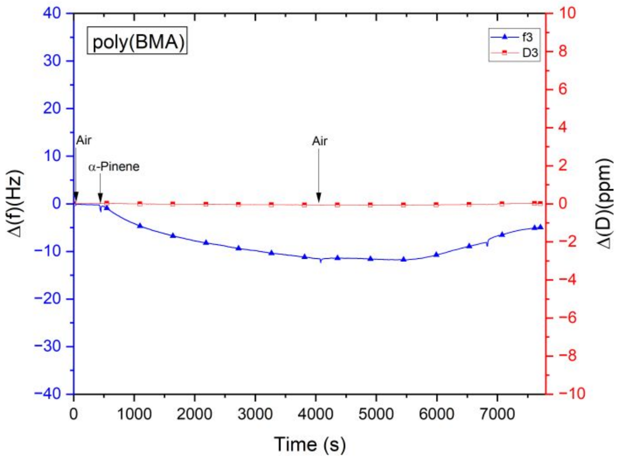

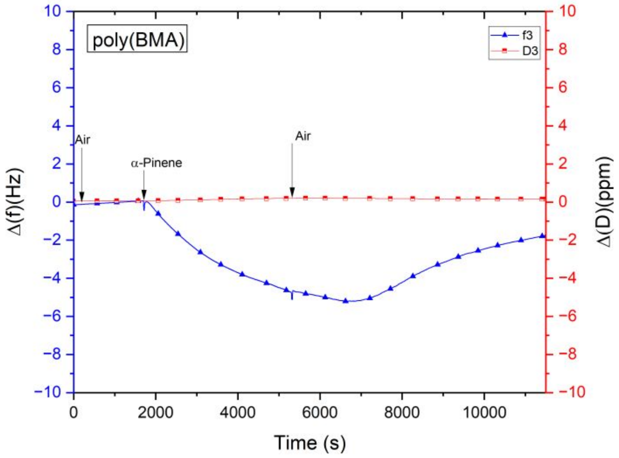

</td>
</tr>
<tr>
<td>SiO_2</td>
<td>
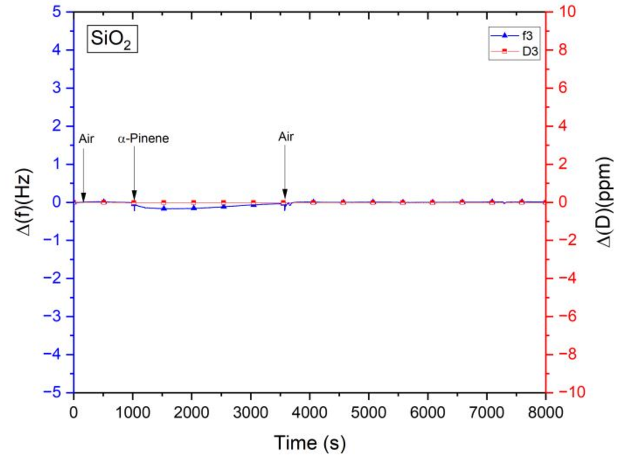

</td>
</tr>
</table>

Table S10. Representative QCM-D sensorgrams of exposure of different polymer brushes and bare silicon oxide surface (SiO_2) coatings to 2-propanol (5 ppt) vapor.

Surface	Representative QCM-D sensograms
Poly(HPMA)	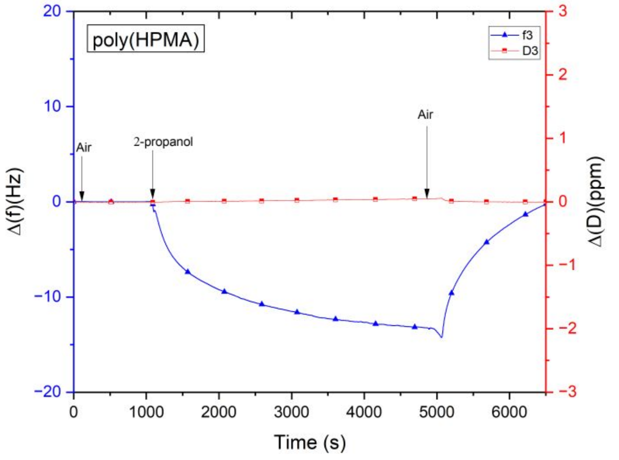<br>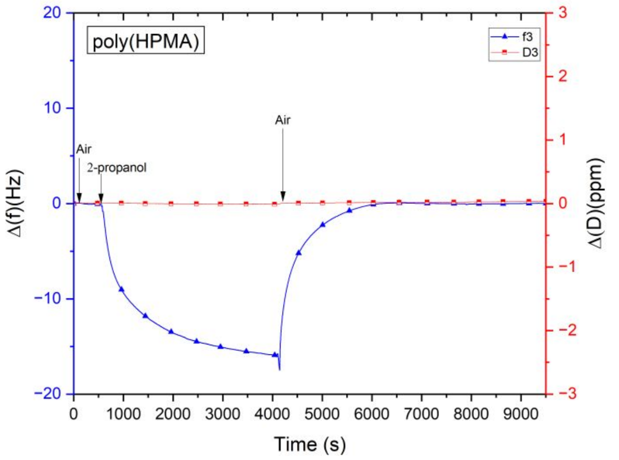<br>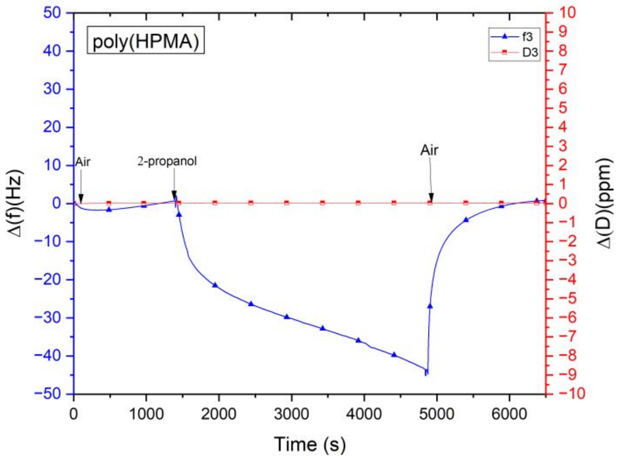

Poly(CBMA)	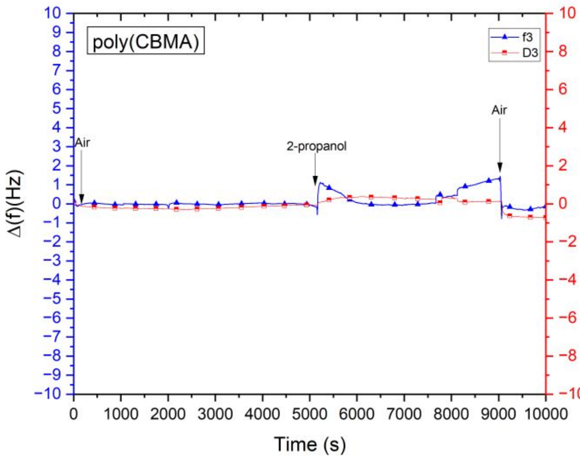 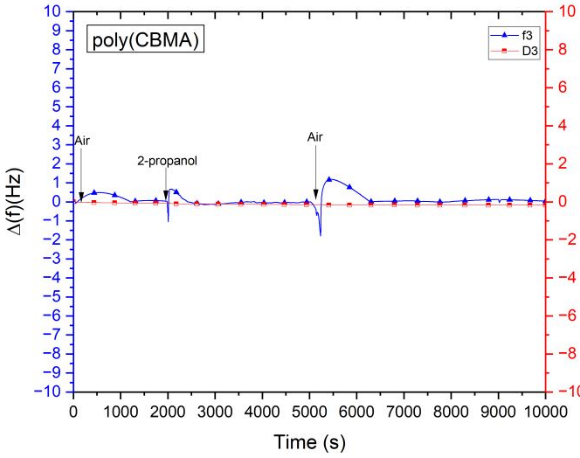
Poly(MeOEGMA)	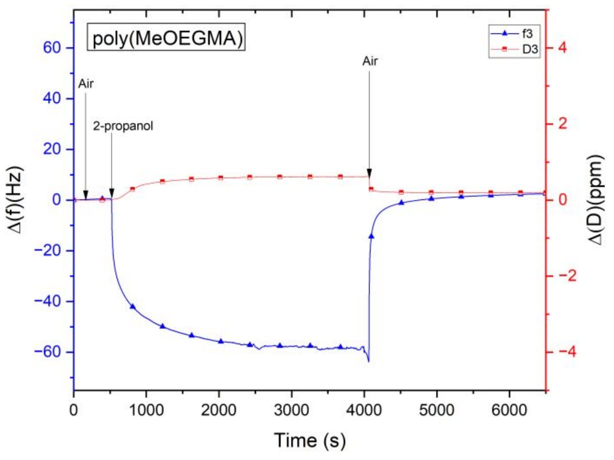

	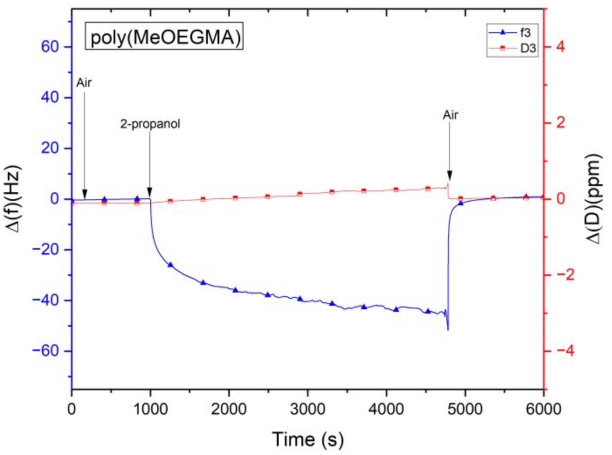
Poly(BMA)	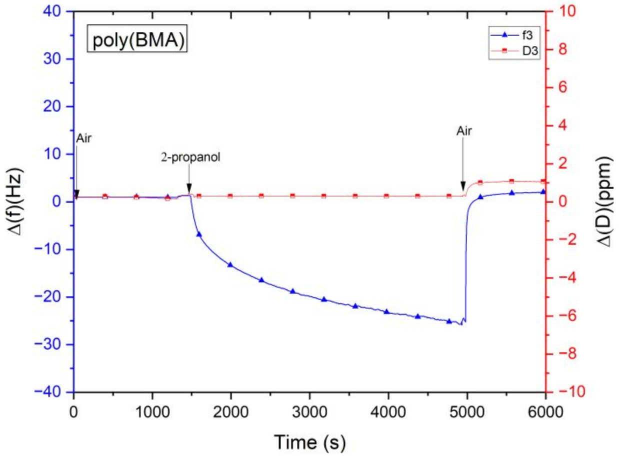
SiO_2	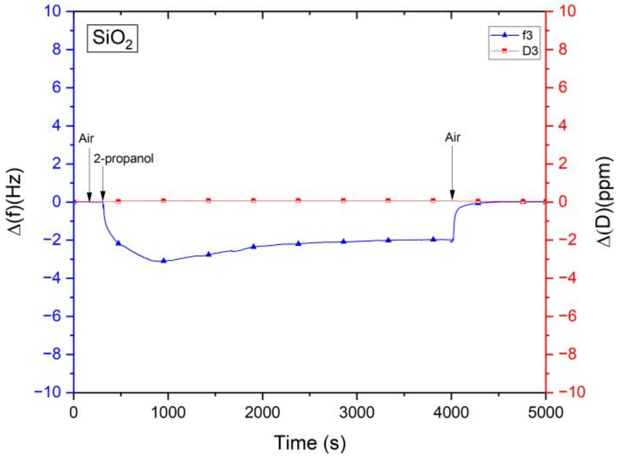

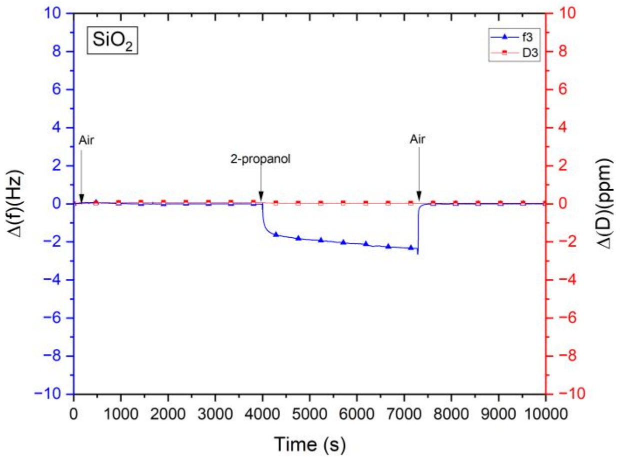

SiO2
f3
D3
Air
2-propanol
Air
Δ(f)(Hz)
Δ(D)(ppm)
Time (s)

Table S10. Representative QCM-D sensorgrams of exposure of different polymer brush and bare silicon oxide surface (SiO_2) coatings to limonene (1 ppt) vapor.

Surface	Representative QCM-D sensograms
Poly(HPMA)	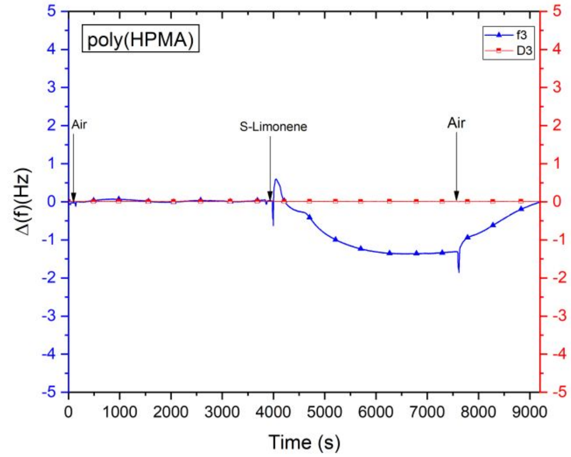 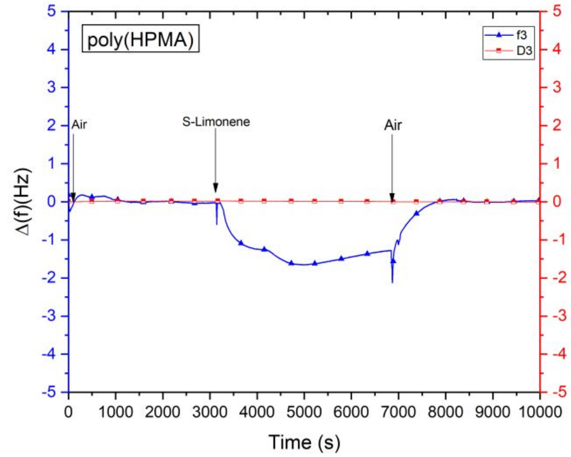
Poly(CBMA)	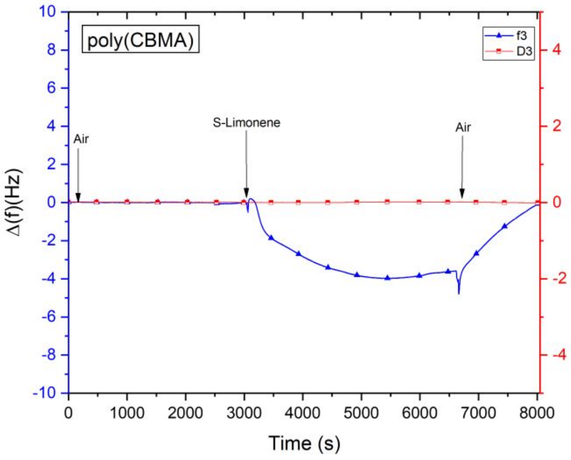

Poly(MeOEGMA)	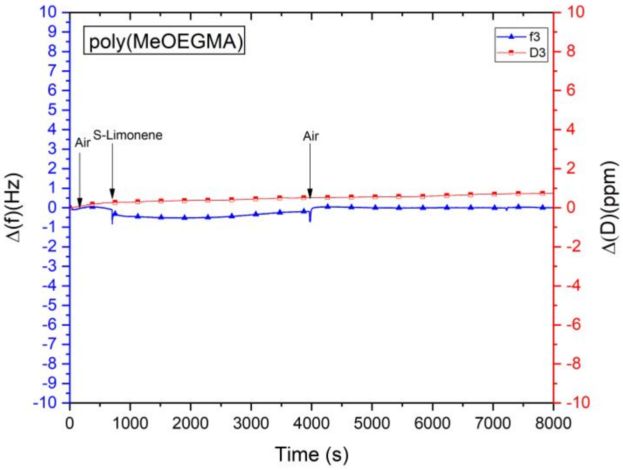<br>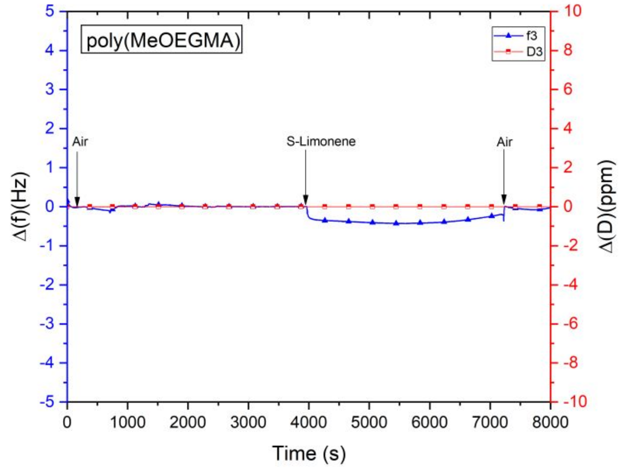
Poly(BMA)	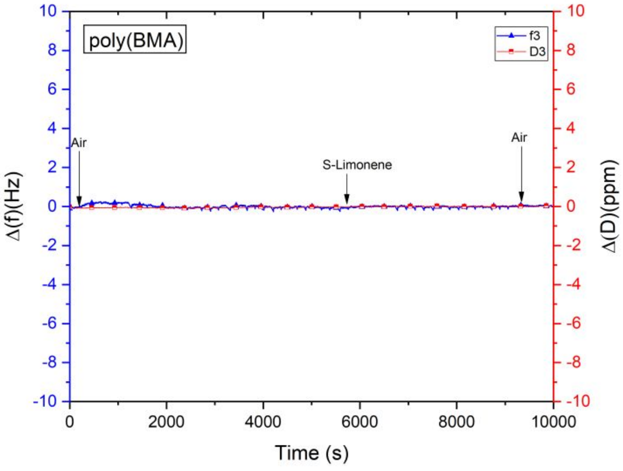

	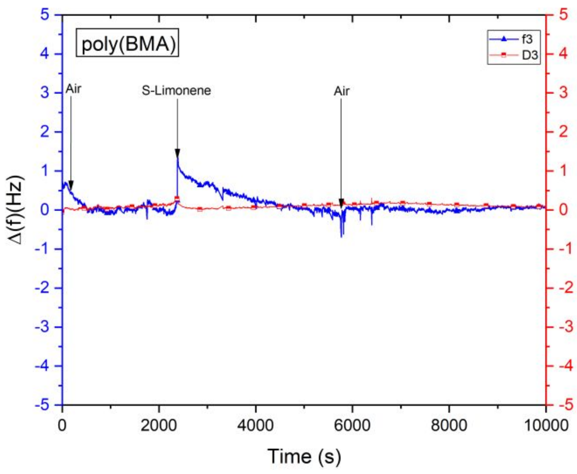poly(BMA)<br>f3<br>D3<br>Air<br>S-Limonene<br>Air<br>Δ(f)(Hz)<br>Δ(D)(ppm)<br>Time (s)
SiO_2	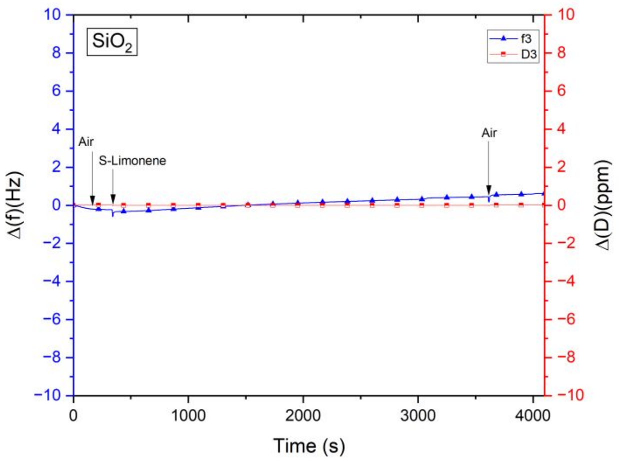SiO2<br>f3<br>D3<br>Air<br>S-Limonene<br>Air<br>Δ(f)(Hz)<br>Δ(D)(ppm)<br>Time (s)

Table S11. Representative QCM-D sensorgrams of exposure of different polymer brush and bare silicon oxide surface (SiO_2) coatings to S-carvone (2 ppt) vapor.

**Surface**	**Representative QCM-D sensograms**
Poly(HPMA)	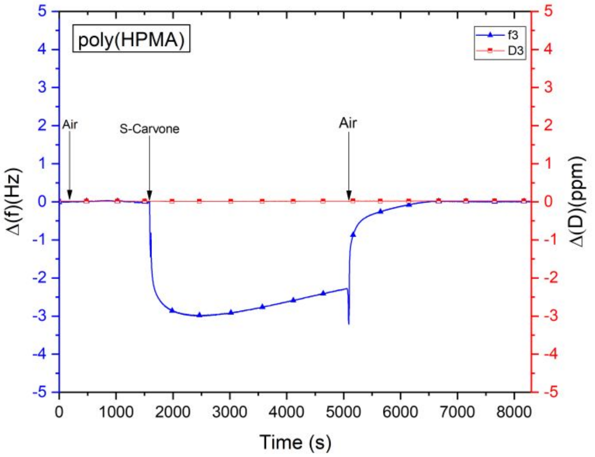<br>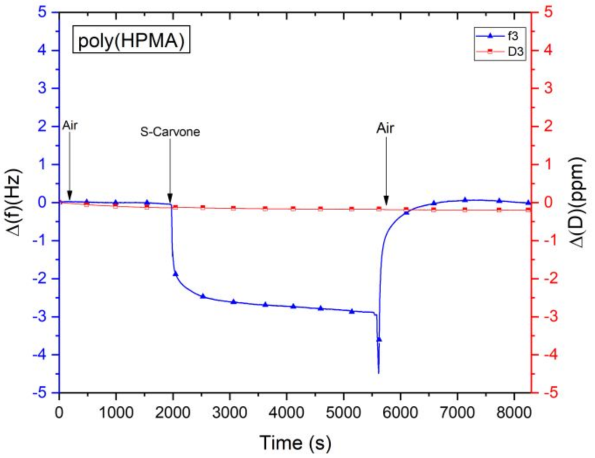

<table>
<tr><td></td><td>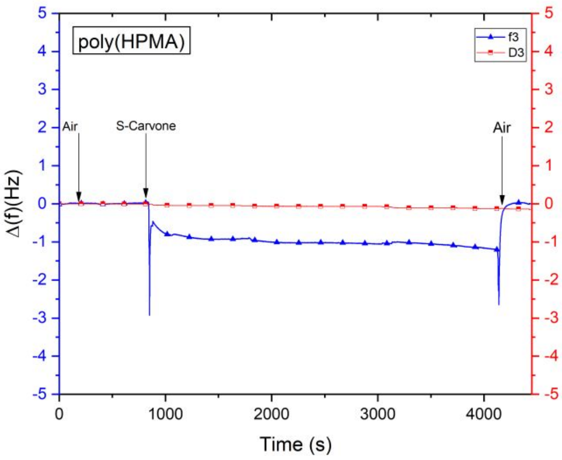
</td></tr>
<tr><td>Poly(CBMA)</td><td>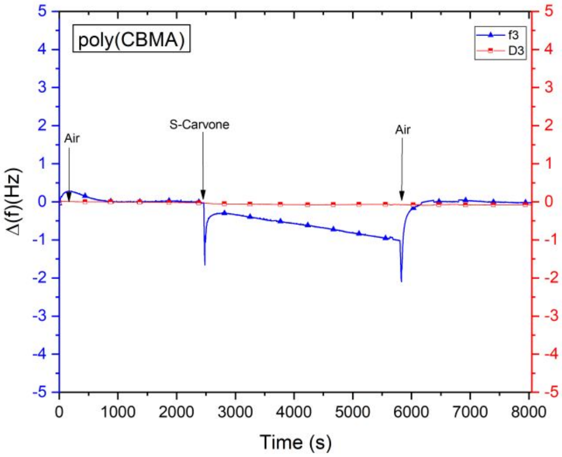

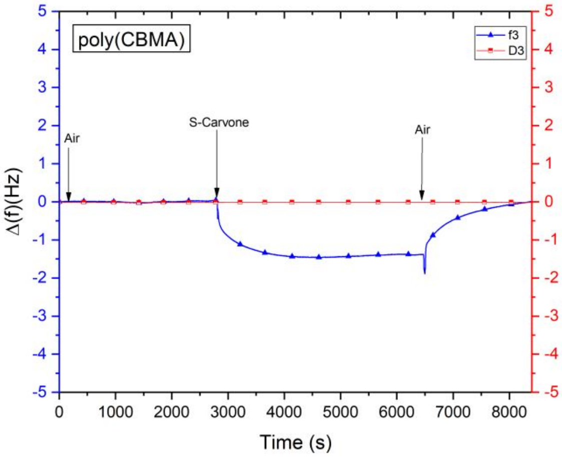
</td></tr>
</table>

Poly(MeOEGMA)	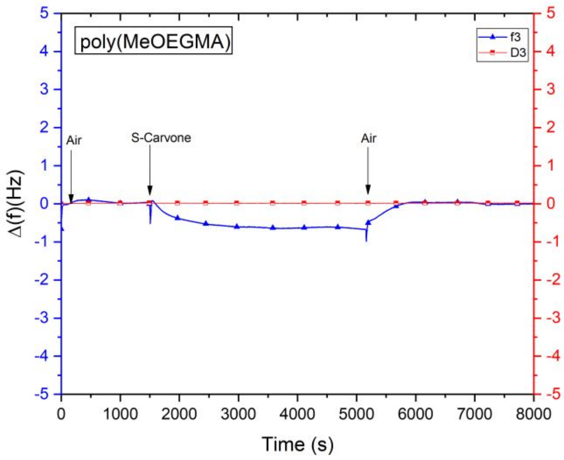<br>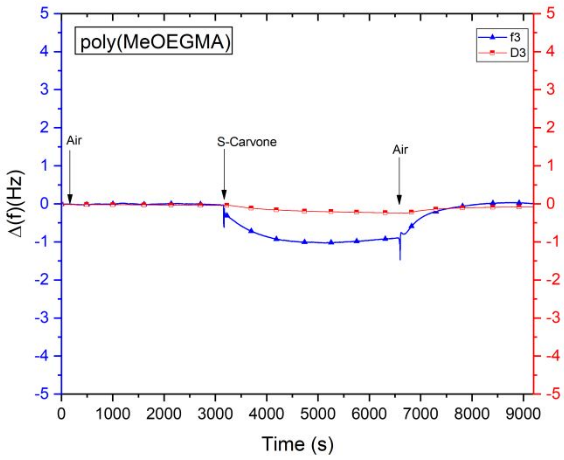
Poly(BMA)	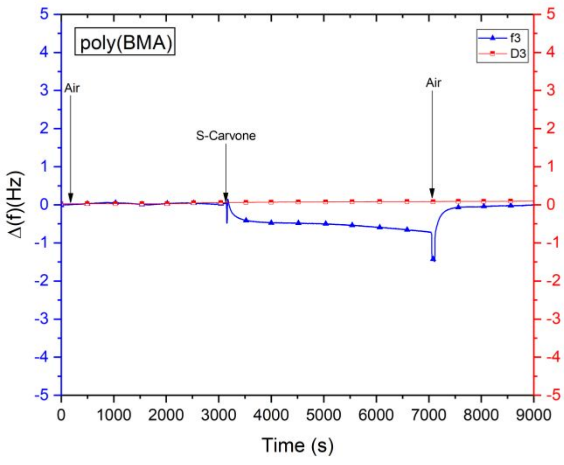

	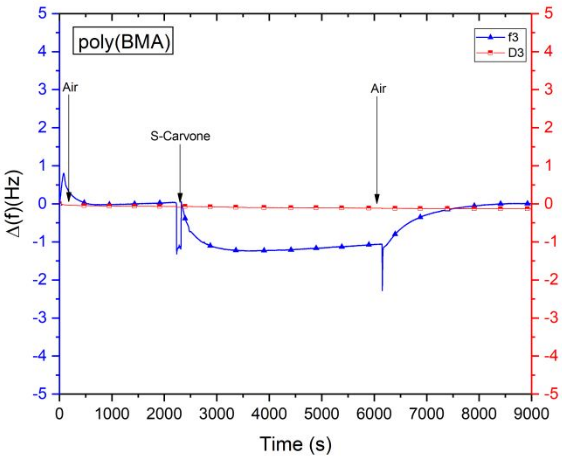poly(BMA)<br>f3<br>D3<br>Air<br>S-Carvone<br>Air<br>Δ(f)(Hz)<br>Δ(D)(ppm)<br>Time (s)
SiO_2	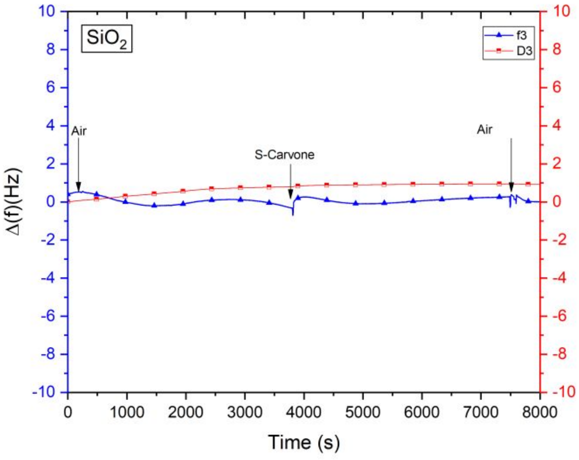SiO_2<br>f3<br>D3<br>Air<br>S-Carvone<br>Air<br>Δ(f)(Hz)<br>Δ(D)(ppm)<br>Time (s)<br>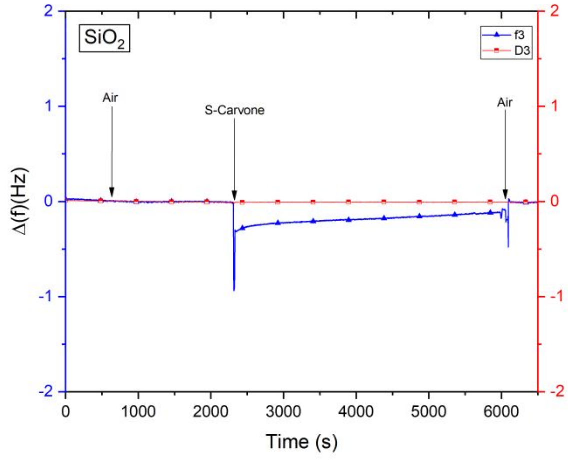SiO_2<br>f3<br>D3<br>Air<br>S-Carvone<br>Air<br>Δ(f)(Hz)<br>Δ(D)(ppm)<br>Time (s)

Table S12. Representative QCM-D sensorgrams of exposure of different polymer brush and bare silicon oxide surface (SiO_2) coatings to lavender oil vapor.

Surface	Representative QCM-D sensograms
Poly(HPMA)	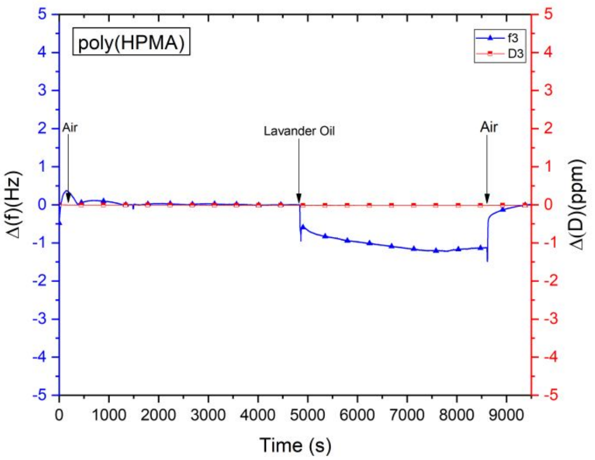<br>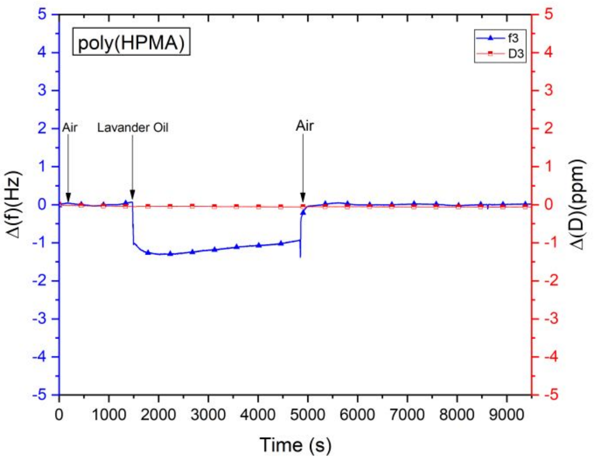

Poly(CBMA)	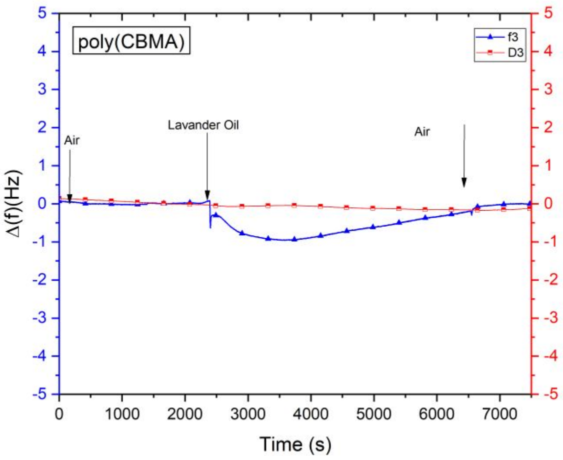<br>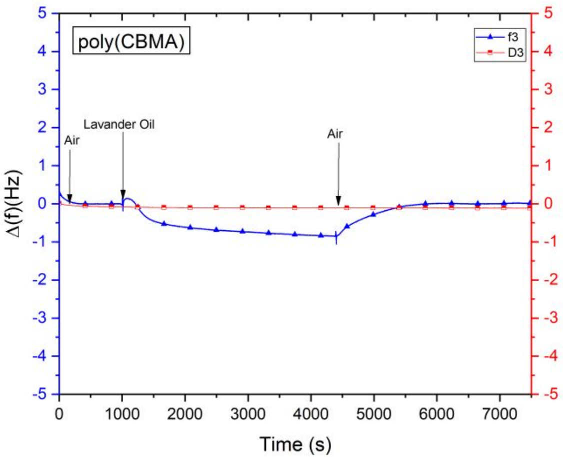
Poly(MeOEGMA)	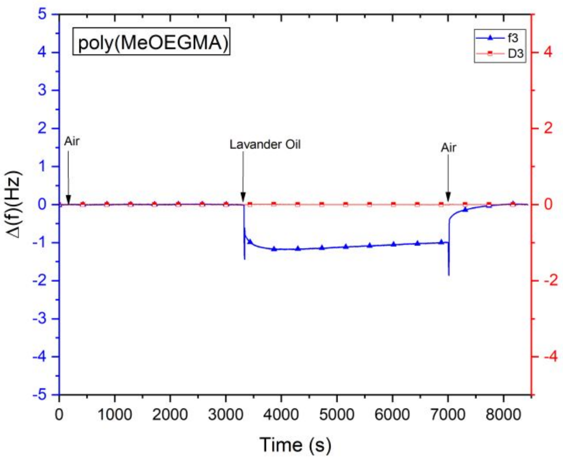

	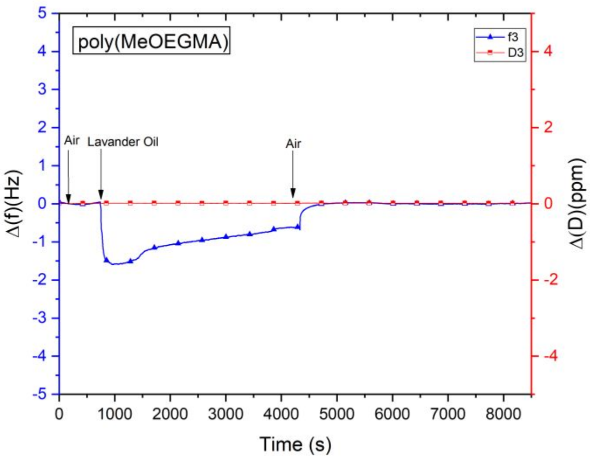poly(MeOEGMA)<br>f3<br>D3<br>Air<br>Lavander Oil<br>Air<br>Δ(f)(Hz)<br>Δ(D)(ppm)<br>Time (s)
Poly(BMA)	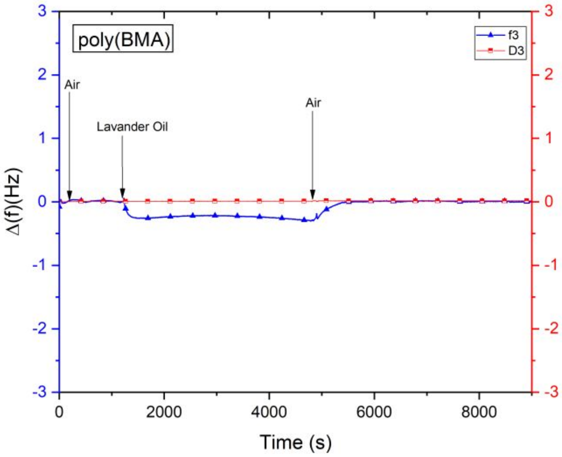poly(BMA)<br>f3<br>D3<br>Air<br>Lavander Oil<br>Air<br>Δ(f)(Hz)<br>Δ(D)(ppm)<br>Time (s)<br>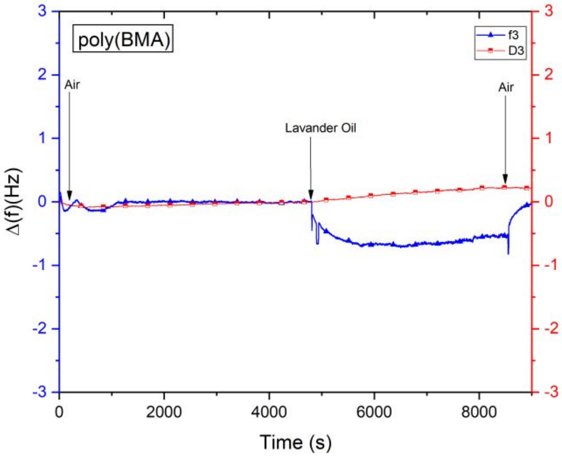poly(BMA)<br>f3<br>D3<br>Air<br>Lavander Oil<br>Air<br>Δ(f)(Hz)<br>Δ(D)(ppm)<br>Time (s)

SiO_2	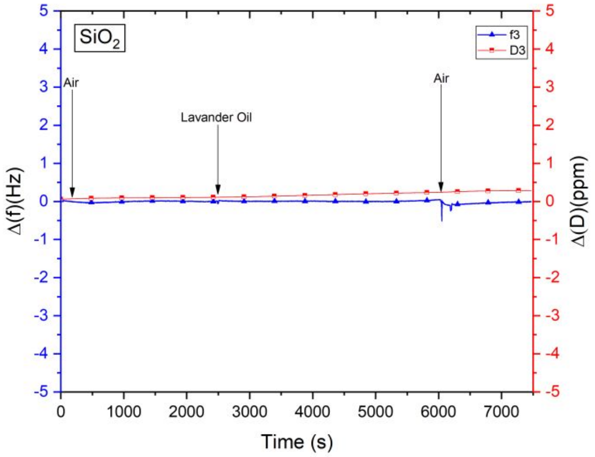SiO2<br>f3<br>D3<br>Air<br>Lavander Oil<br>Air<br>Δ(f)(Hz)<br>Δ(D)(ppm)<br>Time (s)<br>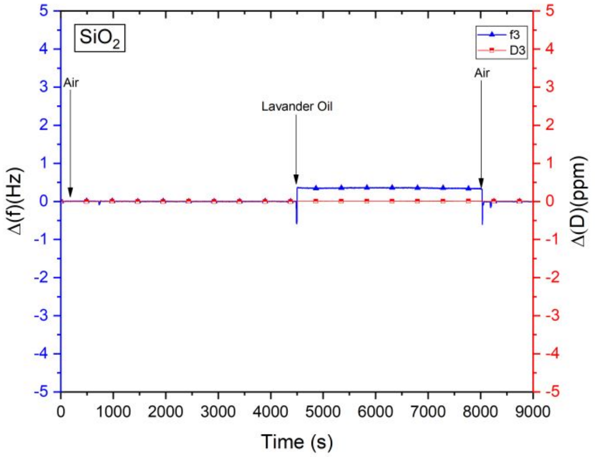SiO2<br>f3<br>D3<br>Air<br>Lavander Oil<br>Air<br>Δ(f)(Hz)<br>Δ(D)(ppm)<br>Time (s)

Table S13. Representative QCM-D sensorgrams of exposure of different polymer brush and bare silicon oxide surface (SiO_2) coatings to rosemary oil vapor.

Surface	Representative QCM-D sensograms
Poly(HPMA)	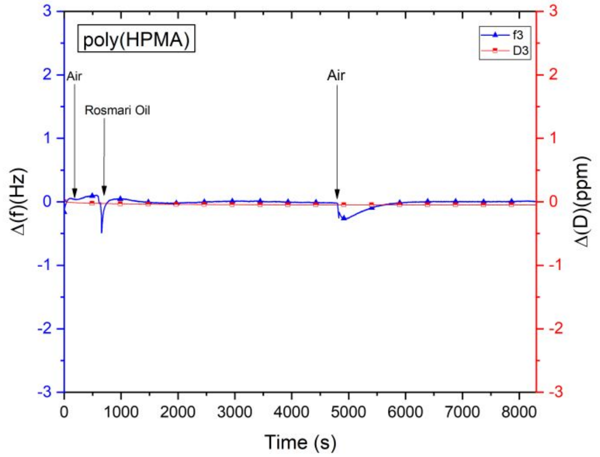 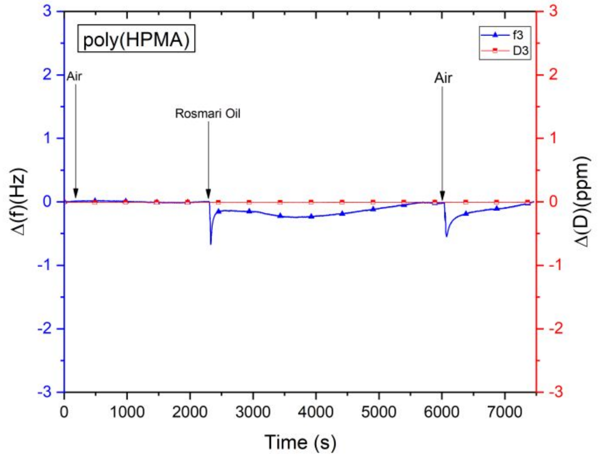
Poly(CBMA)	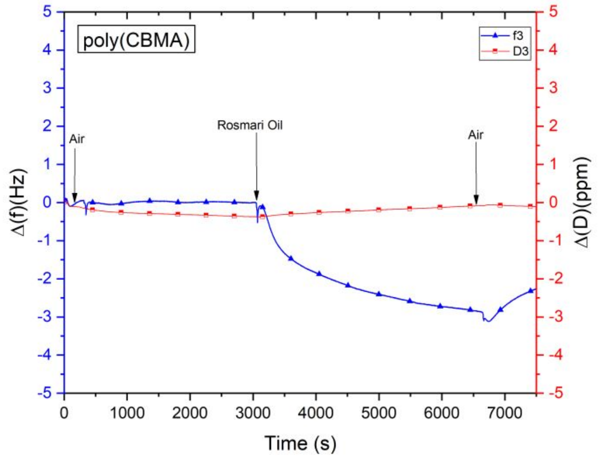

	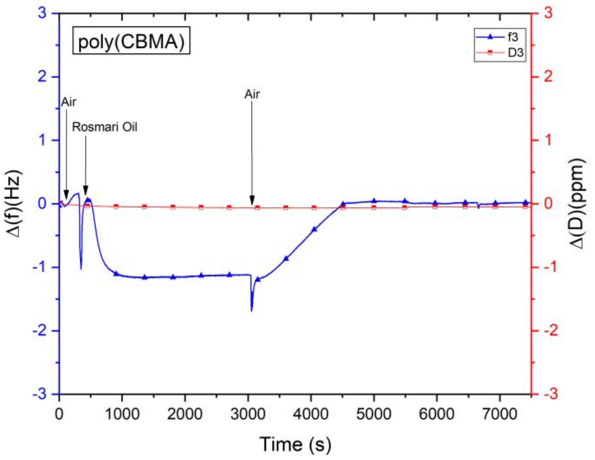
Poly(MeOEGMA)	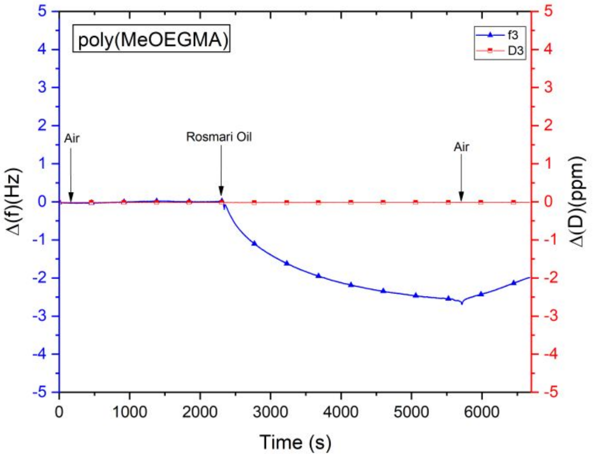 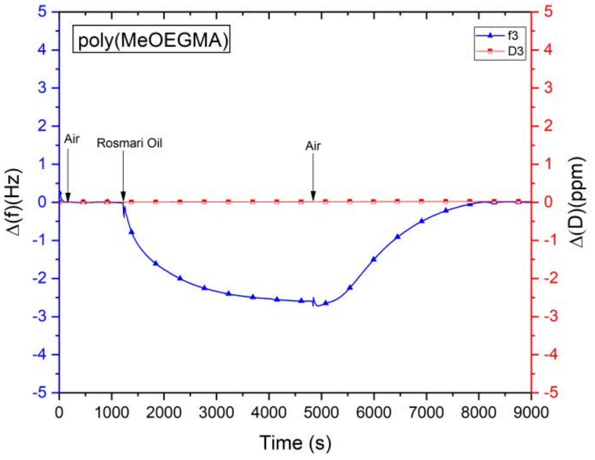

Poly(BMA)	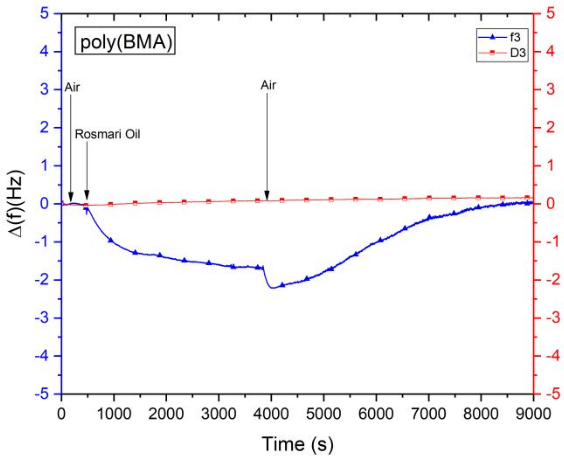 poly(BMA)<br>f3<br>D3<br>Air<br>Rosmari Oil<br>Air<br>Δ(f)(Hz)<br>Δ(D)(ppm)<br>Time (s) 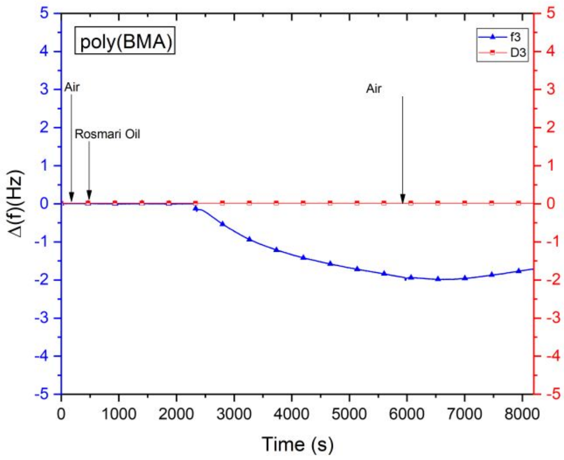 poly(BMA)<br>f3<br>D3<br>Air<br>Rosmari Oil<br>Air<br>Δ(f)(Hz)<br>Δ(D)(ppm)<br>Time (s)

SiO_2	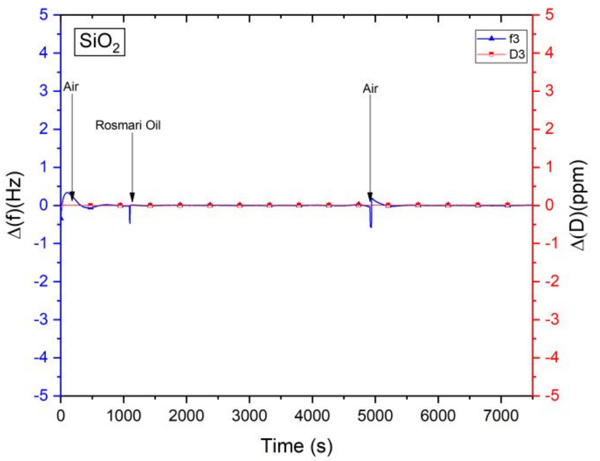 SiO2<br>f3<br>D3<br>Air<br>Rosmari Oil<br>Air<br>Δ(f)(Hz)<br>Δ(D)(ppm)<br>Time (s) 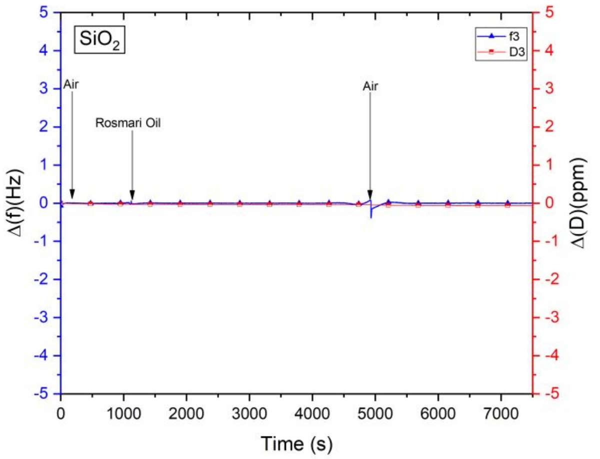 SiO2<br>f3<br>D3<br>Air<br>Rosmari Oil<br>Air<br>Δ(f)(Hz)<br>Δ(D)(ppm)<br>Time (s)

Table S14. Representative QCM-D sensorgrams of exposure of different polymer brush and bare silicon oxide surface (SiO_2) coatings to Jameson whiskey vapor.

<table>
<tr><th>Surface</th><th>Representative QCM-D sensograms</th></tr>
<tr><td>Poly(HPMA)</td><td>
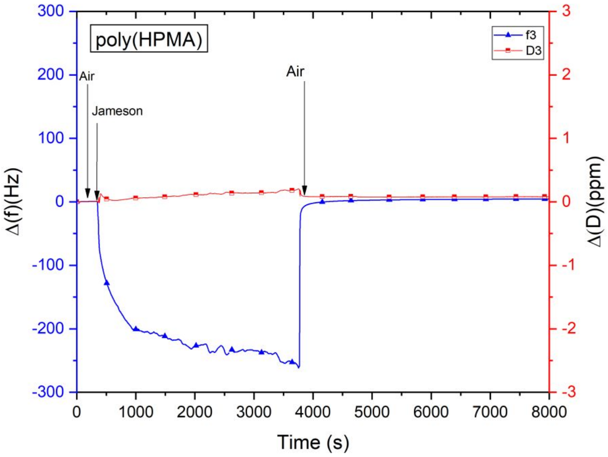

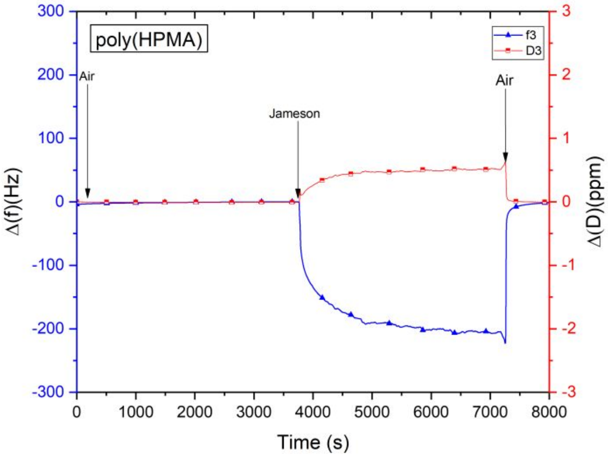

</td></tr>
<tr><td>Poly(CBMA)</td><td>
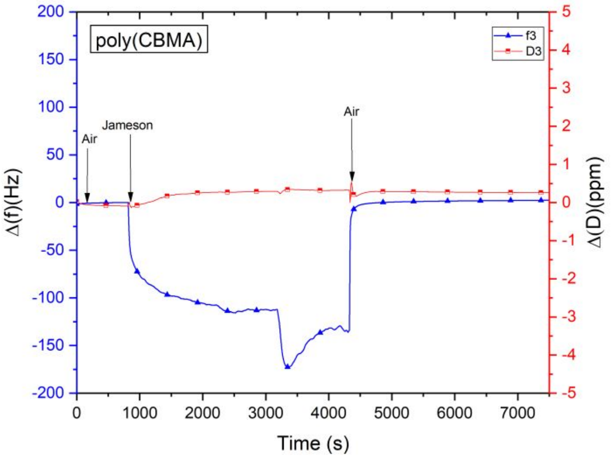

</td></tr>
</table>

	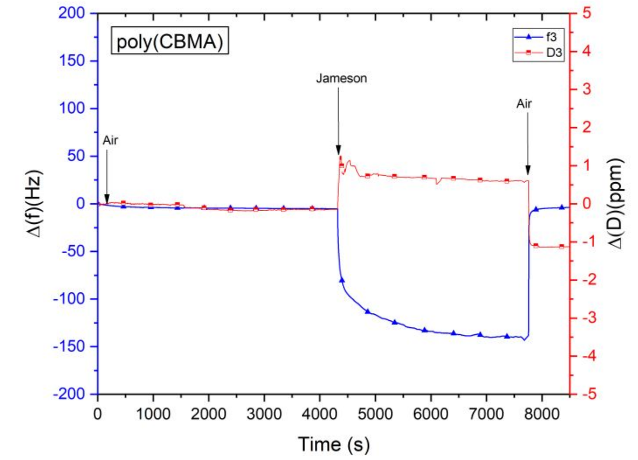
Poly(MeOEGMA)	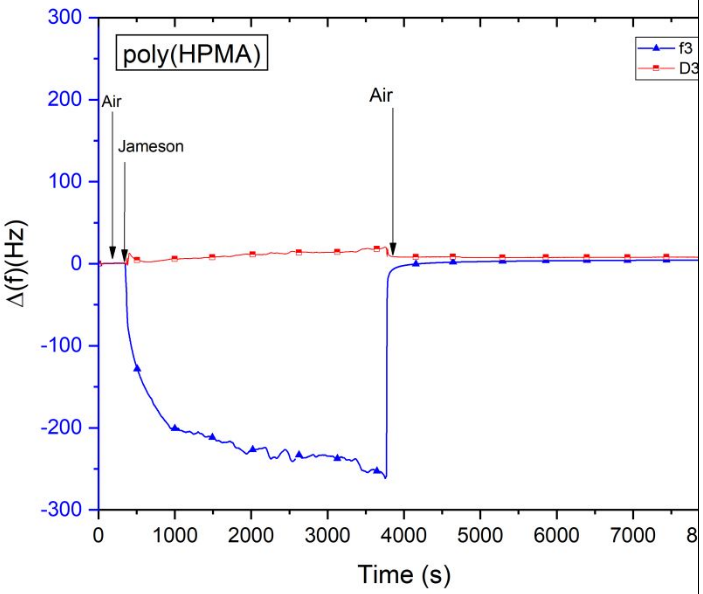 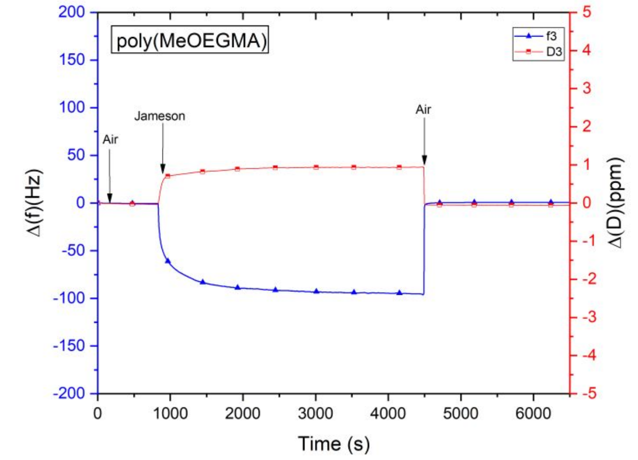

Poly(BMA)

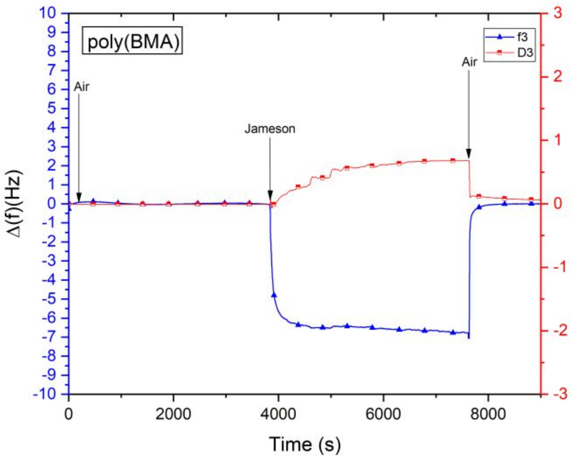

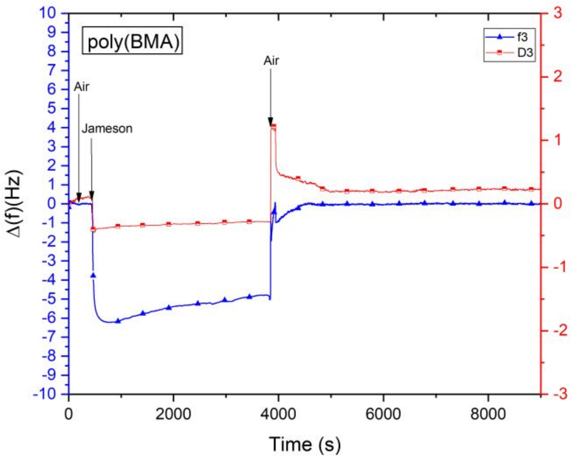

SiO_2

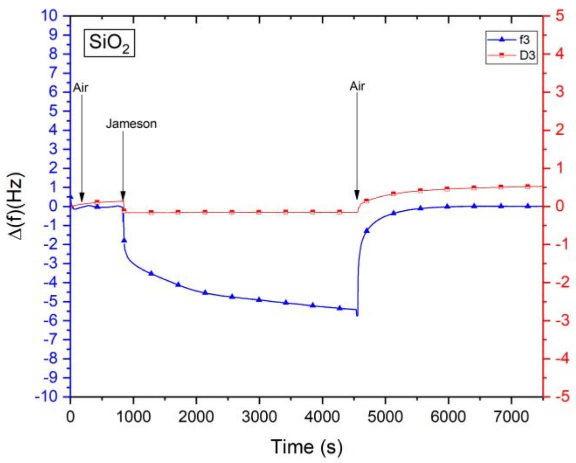

	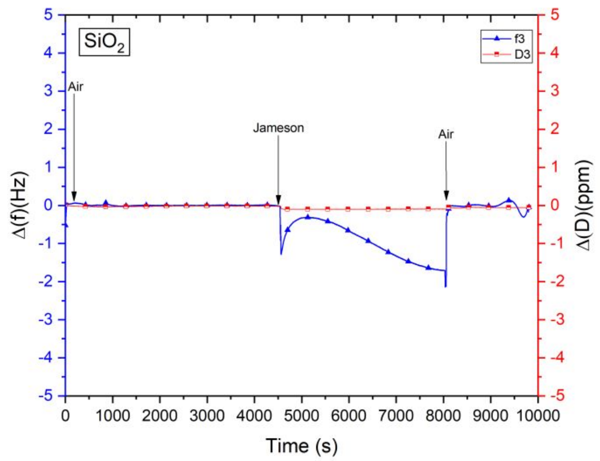SiO2<br>f3<br>D3<br>Air<br>Jameson<br>Air<br>Δ(f)(Hz)<br>Δ(D)(ppm)<br>Time (s)<br>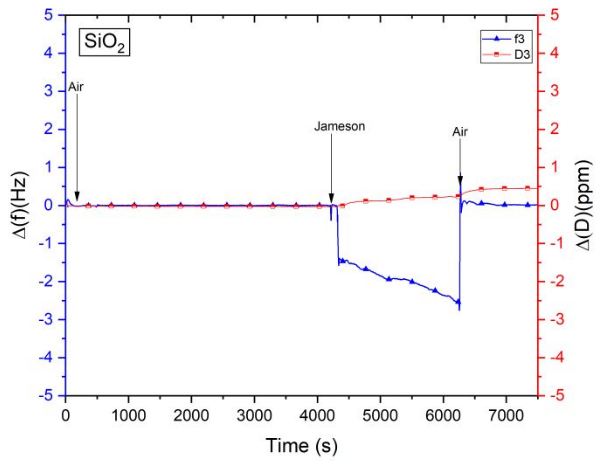SiO2<br>f3<br>D3<br>Air<br>Jameson<br>Air<br>Δ(f)(Hz)<br>Δ(D)(ppm)<br>Time (s)<br>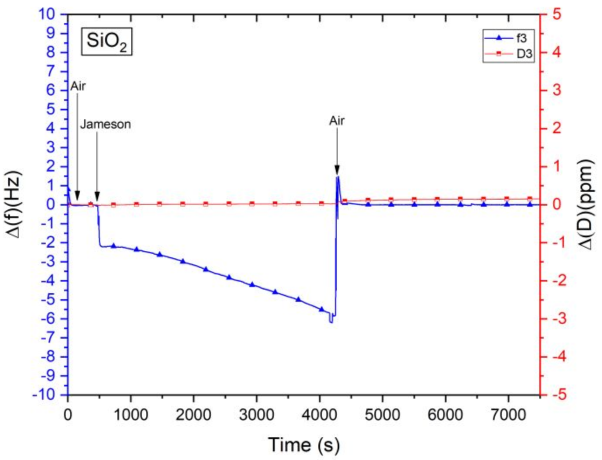SiO2<br>f3<br>D3<br>Air<br>Jameson<br>Air<br>Δ(f)(Hz)<br>Δ(D)(ppm)<br>Time (s)

Table S15. Representative QCM-D sensorgrams of exposure of different polymer brush and bare silicon oxide surface (SiO_2) coatings to 3-methyl-butanol (1 ppm) vapor.

<table>
<tr><th>Surface</th><th>Representative QCM-D sensograms</th></tr>
<tr><td>Poly(HPMA)</td><td>
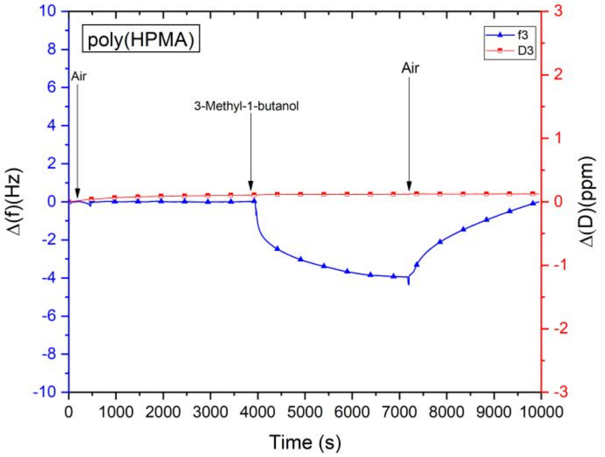

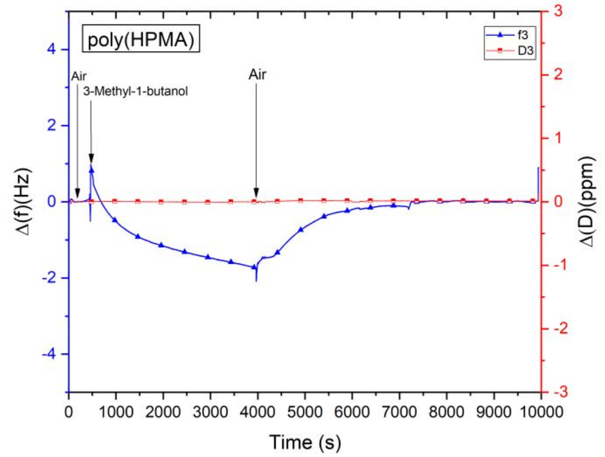

</td></tr>
<tr><td>Poly(CBMA)</td><td>
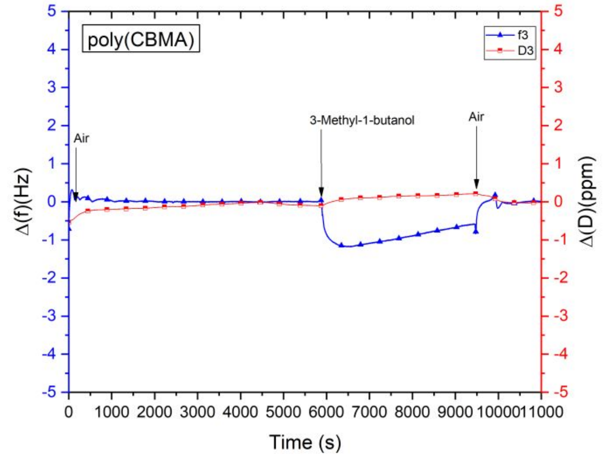

</td></tr>
</table>

	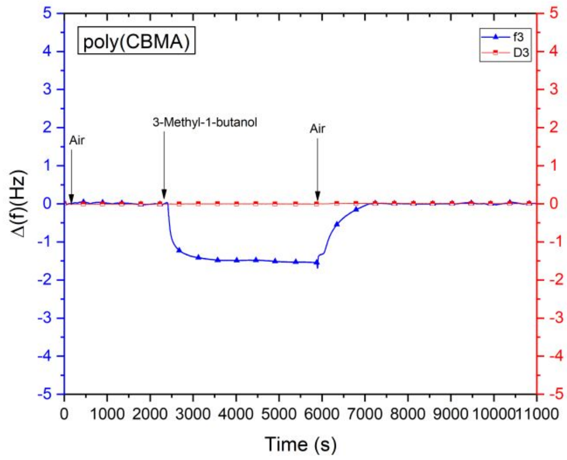
Poly(MeOEGMA)	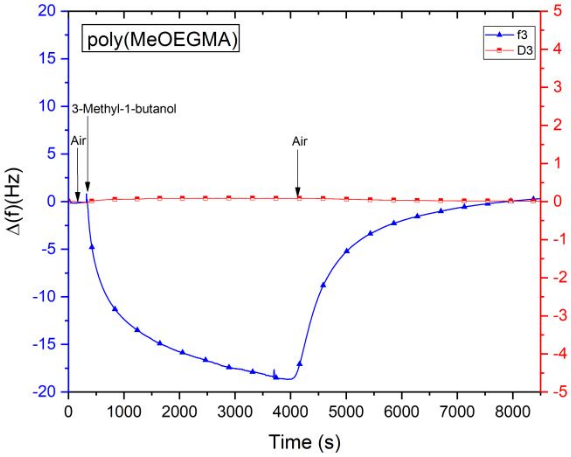<br>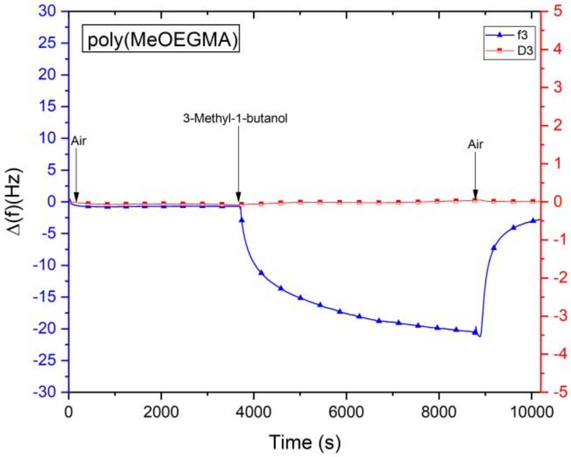

Poly(BMA)	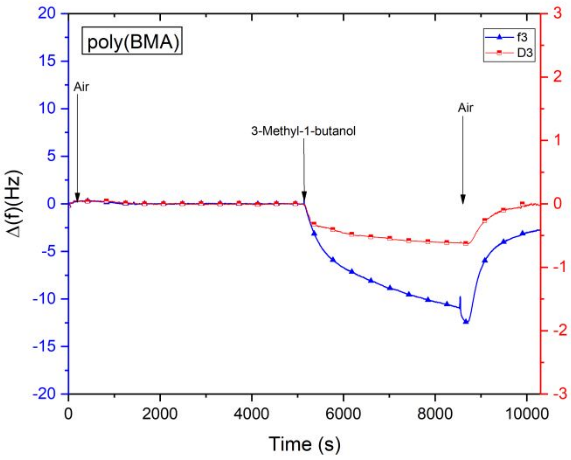 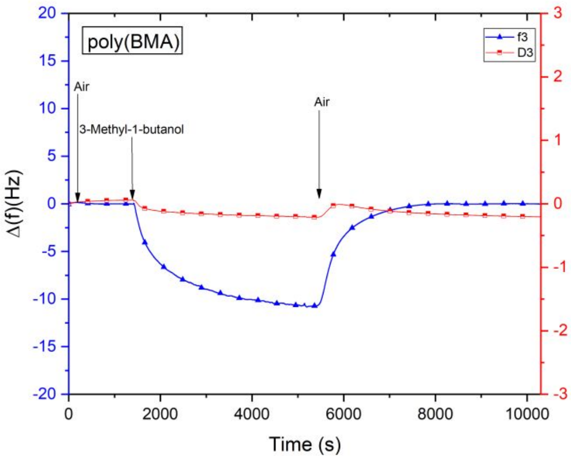
SiO_2	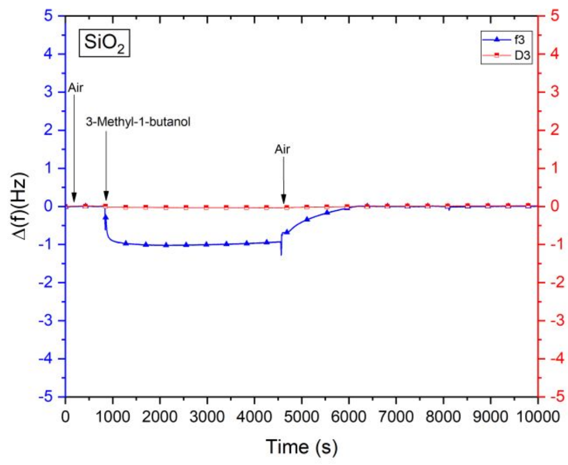

	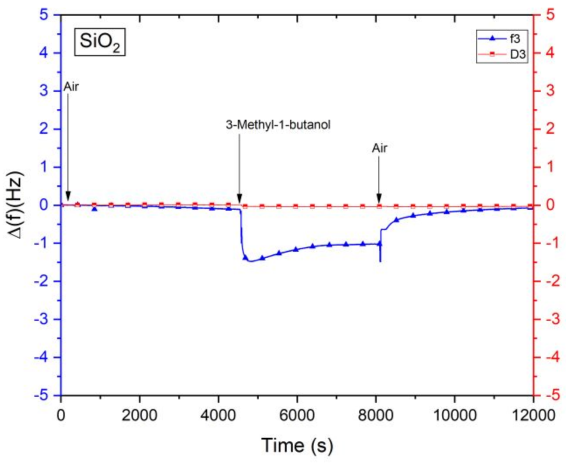SiO2
f3
D3
Air
3-Methyl-1-butanol
Air
Δ(f)(Hz)
Δ(D)(ppm)
Time (s)

Table S16. Representative QCM-D sensorgrams of exposure of different polymer brush and bare silicon oxide surface (SiO_2) coatings to acetone (2 ppt) vapor.

Surface	Representative QCM-D sensograms
Poly(HPMA)	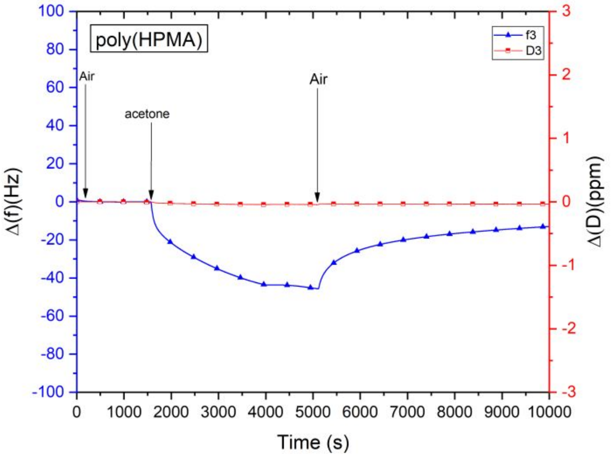<br>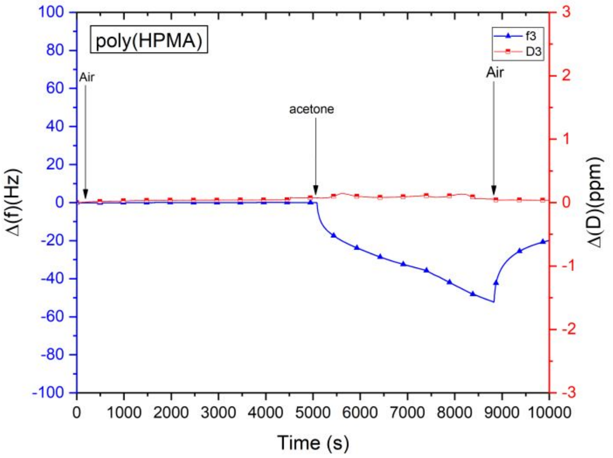

Poly(CBMA)	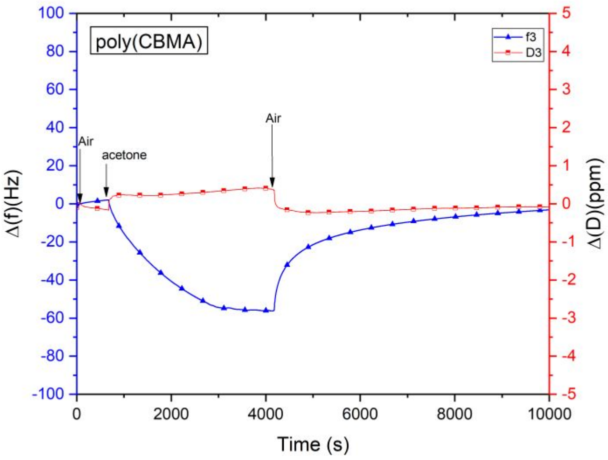 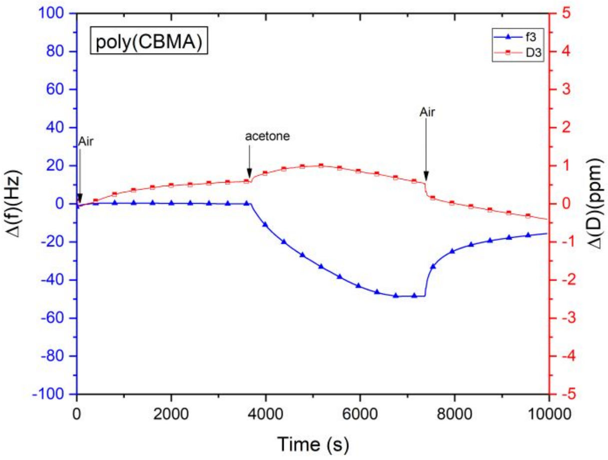 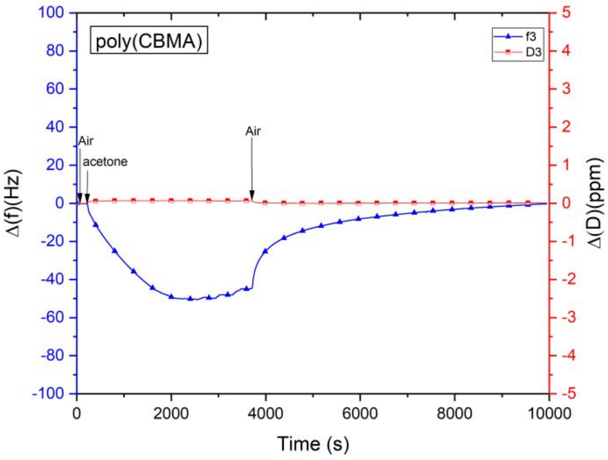

poly(CBMA)
f3
D3
Air
acetone
Air
Δ(f)(Hz)
Δ(D)(ppm)
Time (s)

poly(CBMA)
f3
D3
Air
acetone
Air
Δ(f)(Hz)
Δ(D)(ppm)
Time (s)

poly(CBMA)
f3
D3
Air
acetone
Air
Δ(f)(Hz)
Δ(D)(ppm)
Time (s)

Poly(MeOEGMA)	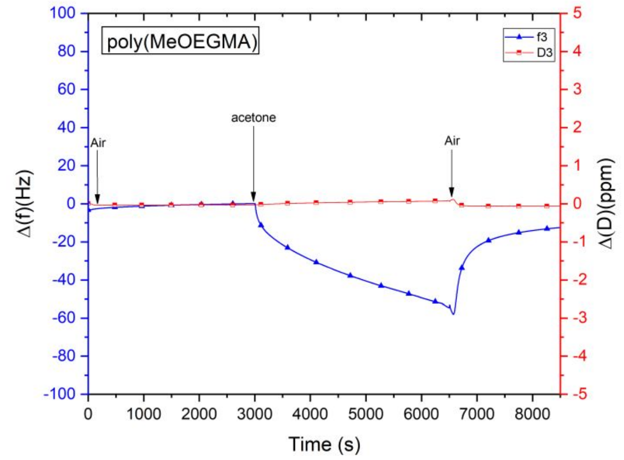 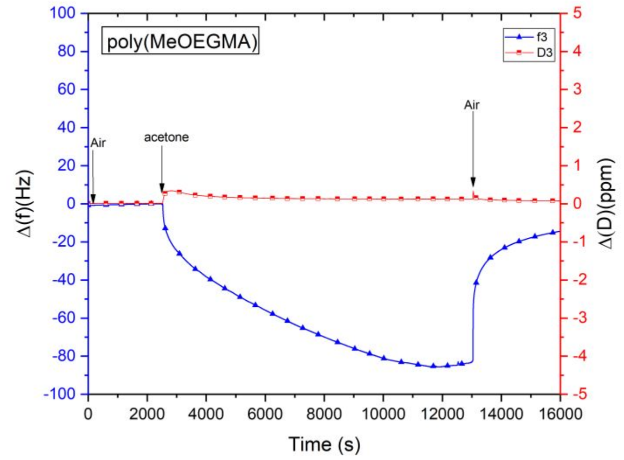 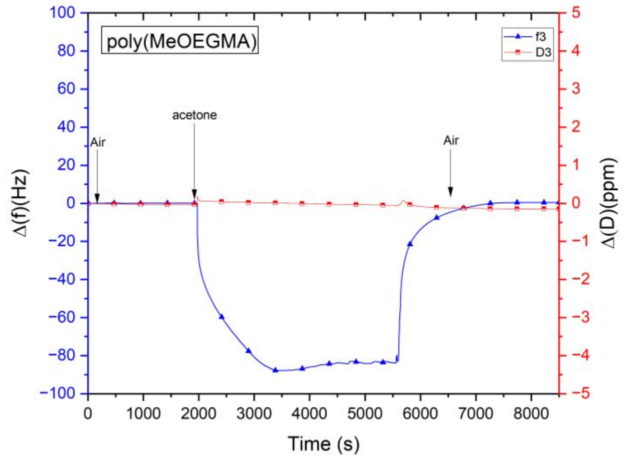

Poly(BMA)	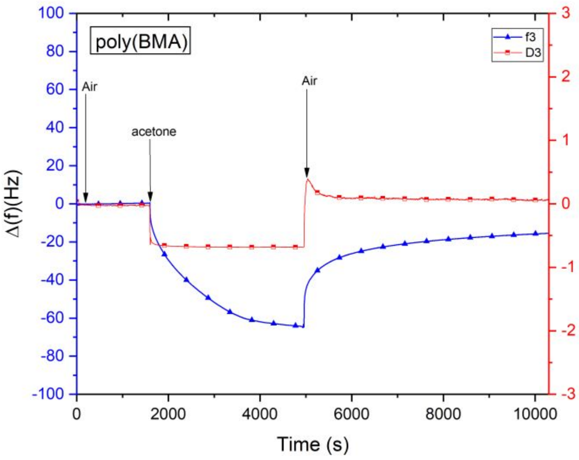<br>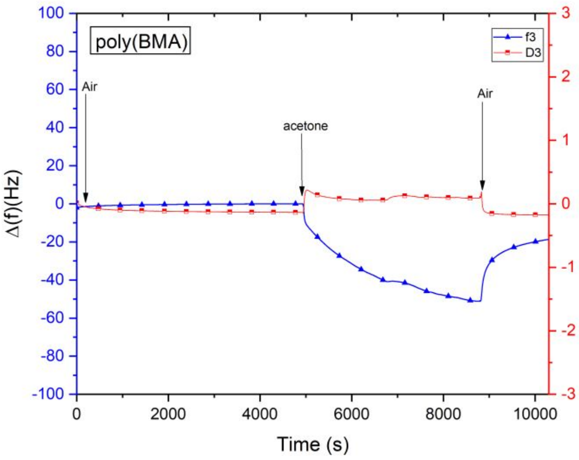
SiO_2	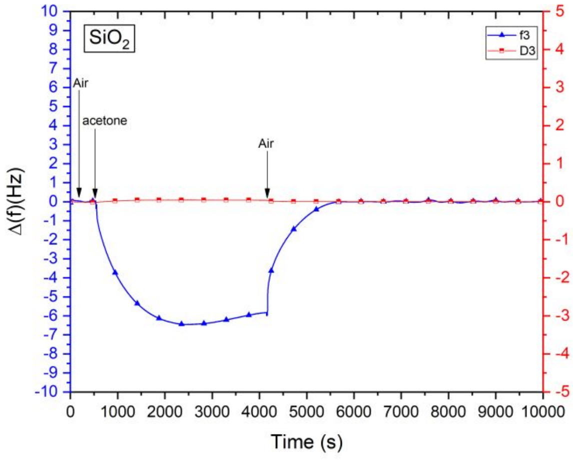

Table S17. Representative QCM-D sensorgrams of exposure of different polymer brush and bare silicon oxide surface (SiO_2) coatings to water-ethanol mixture vapor.

Surface	Representative QCM-D sensograms
Poly(HPMA)	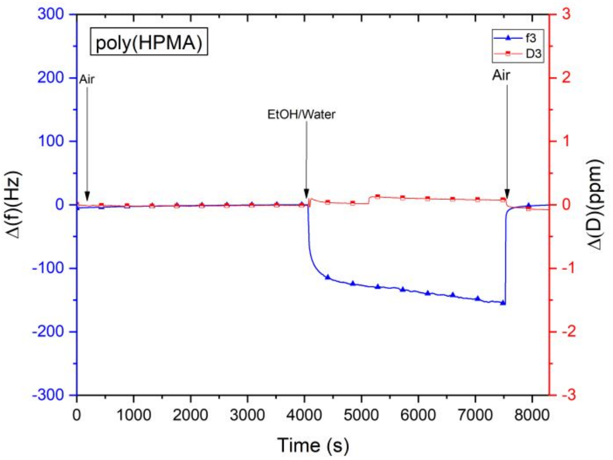 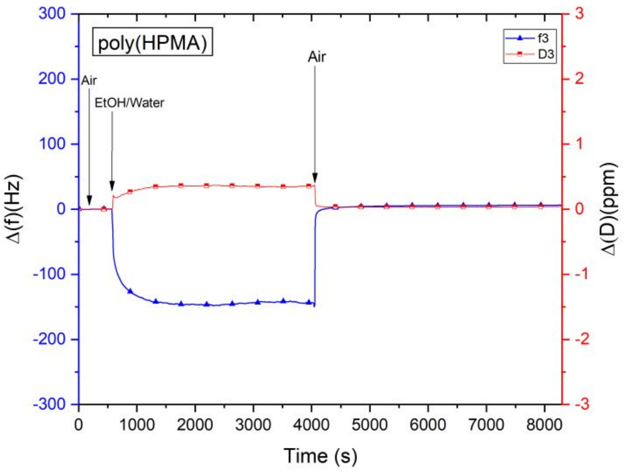

Poly(CBMA)

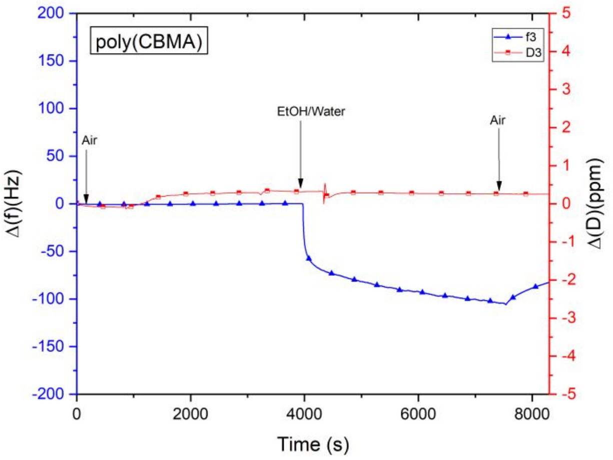
poly(CBMA)
f3
D3
Air
EtOH/Water
Air
Δ(f)(Hz)
Δ(D)(ppm)
Time (s)

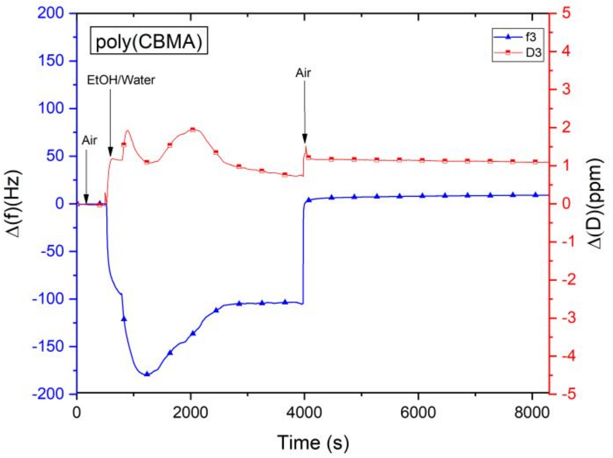
poly(CBMA)
f3
D3
EtOH/Water
Air
Air
Δ(f)(Hz)
Δ(D)(ppm)
Time (s)

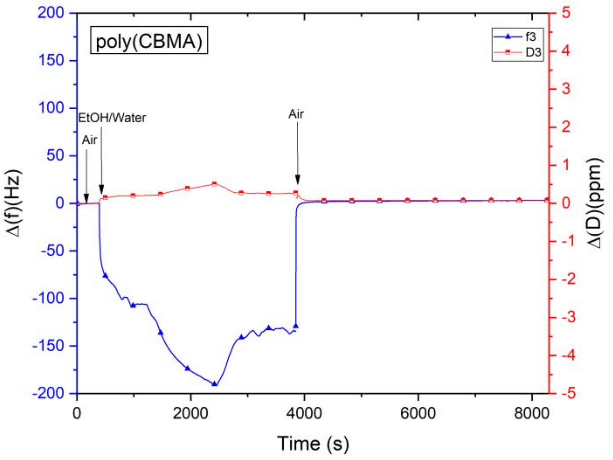
poly(CBMA)
f3
D3
EtOH/Water
Air
Air
Δ(f)(Hz)
Δ(D)(ppm)
Time (s)

Poly(MeOEGMA)	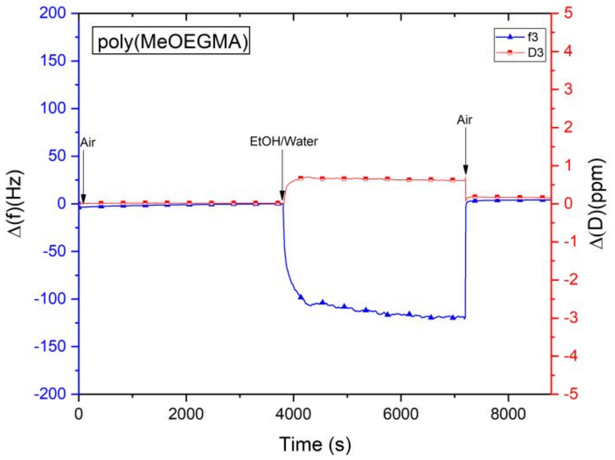<br>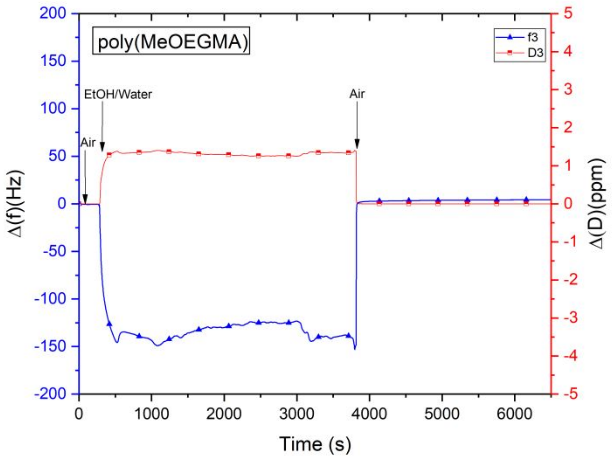

Poly(BMA)	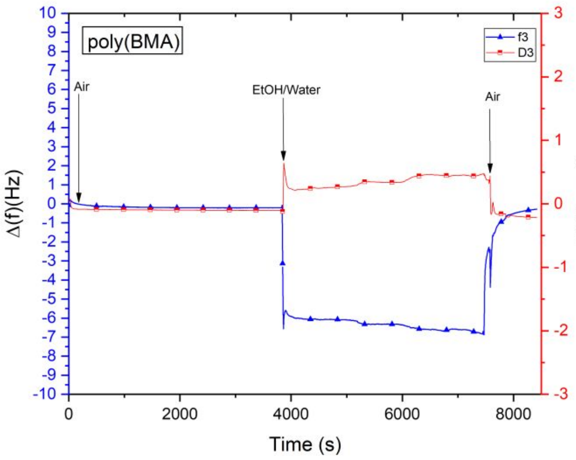 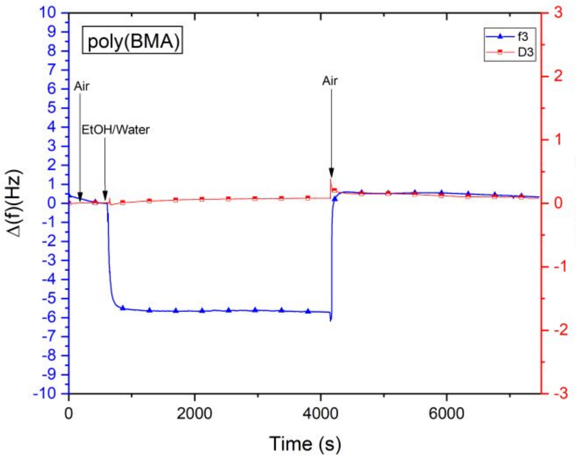 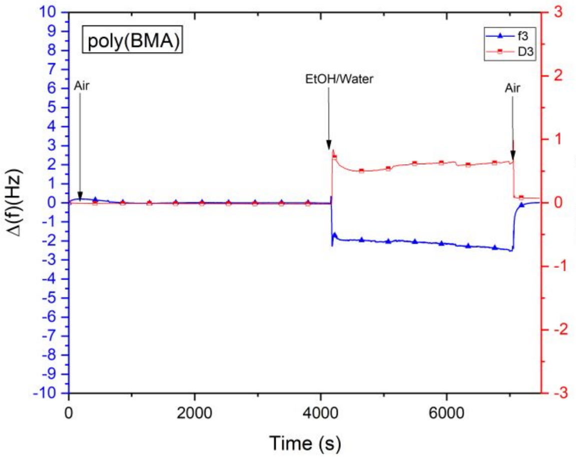

SiO_2	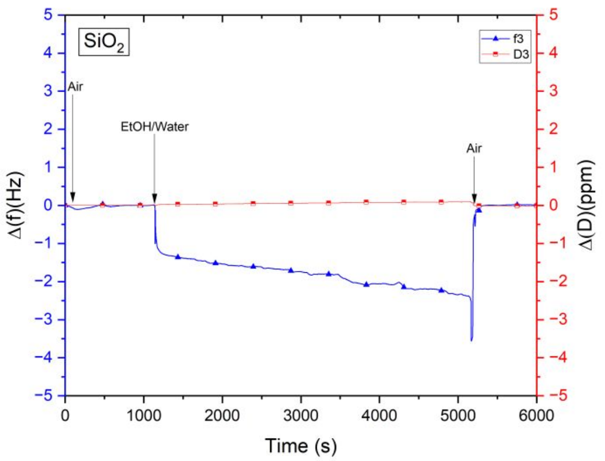

Table S17. Representative QCM-D sensorgrams of exposure of different polymer brush and bare silicon oxide surface (SiO_2) coatings to Jack Daniels whiskey vapor.

Surface	Representative QCM-D sensograms
Poly(HPMA)	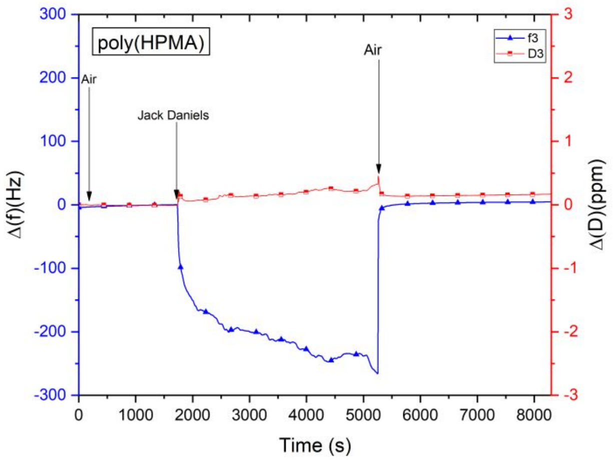 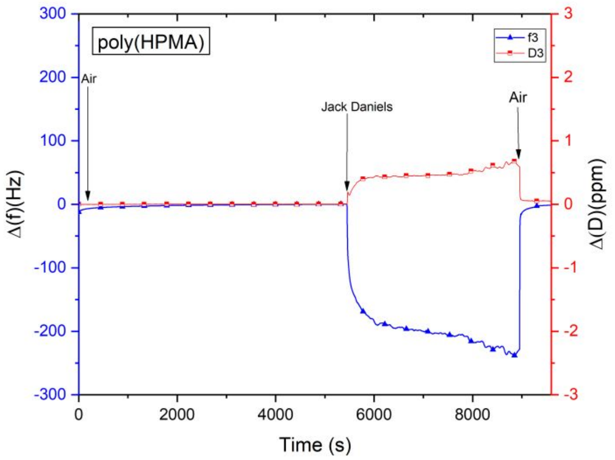

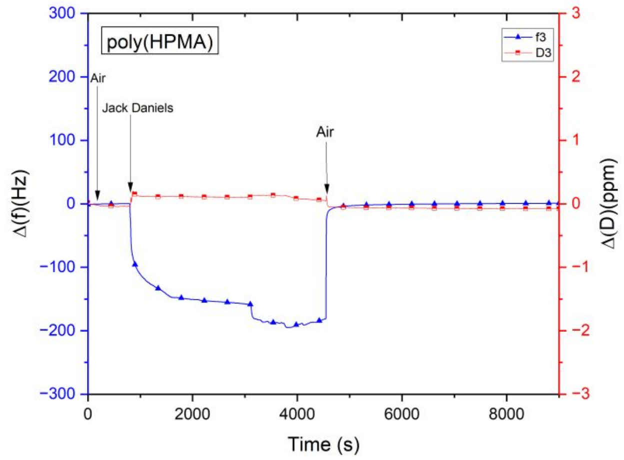
poly(HPMA)
f3
D3
Air
Jack Daniels
Air
Δ(f)(Hz)
Δ(D)(ppm)
Time (s)

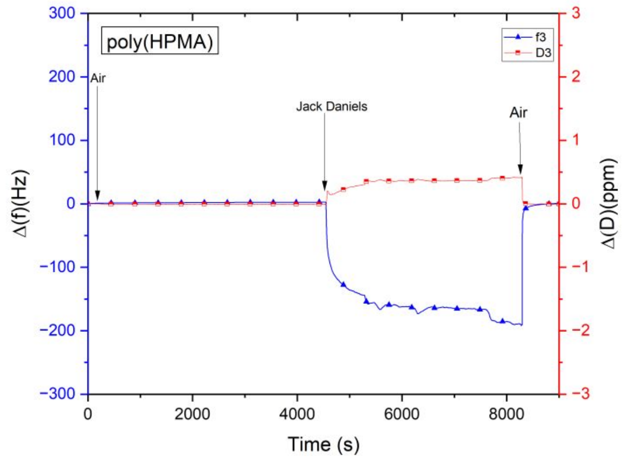
poly(HPMA)
f3
D3
Air
Jack Daniels
Air
Δ(f)(Hz)
Δ(D)(ppm)
Time (s)

	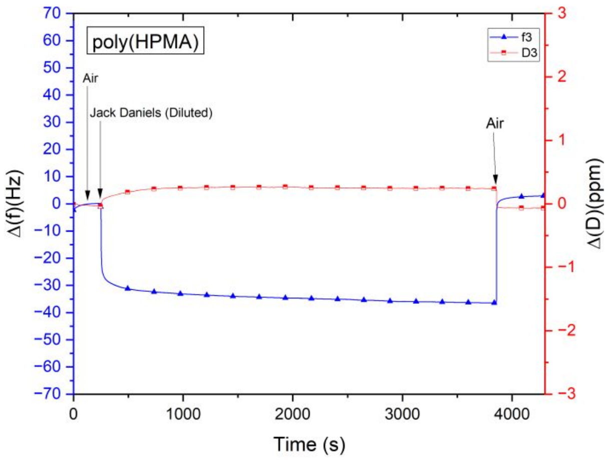poly(HPMA)<br>f3<br>D3<br>Air<br>Jack Daniels (Diluted)<br>Air<br>Δ(f)(Hz)<br>Δ(D)(ppm)<br>Time (s)<br>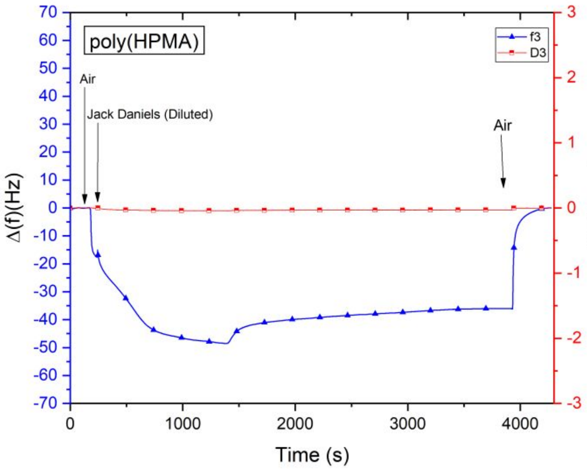poly(HPMA)<br>f3<br>D3<br>Air<br>Jack Daniels (Diluted)<br>Air<br>Δ(f)(Hz)<br>Δ(D)(ppm)<br>Time (s)
Poly(CBMA)	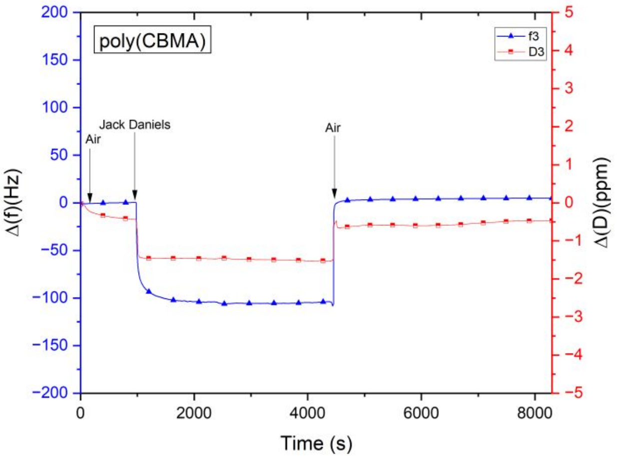poly(CBMA)<br>f3<br>D3<br>Air<br>Jack Daniels<br>Air<br>Δ(f)(Hz)<br>Δ(D)(ppm)<br>Time (s)

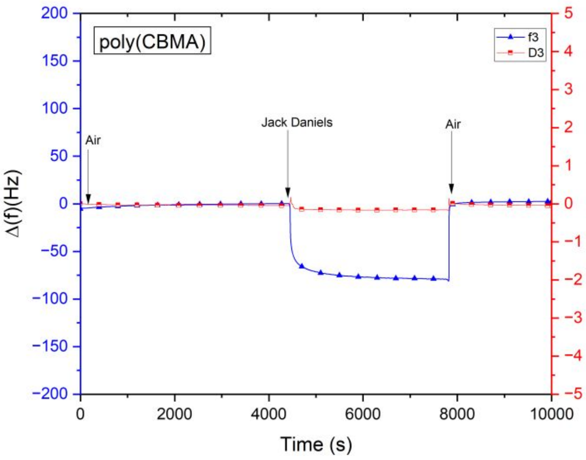
poly(CBMA)
f3
D3
Air
Jack Daniels
Air
Δ(f)(Hz)
Δ(D)(ppm)
Time (s)

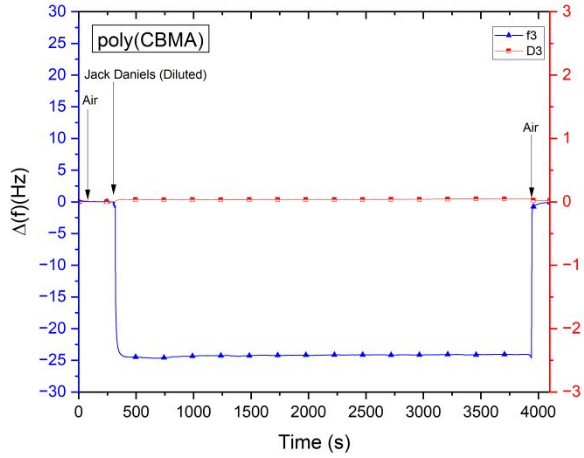
poly(CBMA)
f3
D3
Jack Daniels (Diluted)
Air
Air
Δ(f)(Hz)
Δ(D)(ppm)
Time (s)

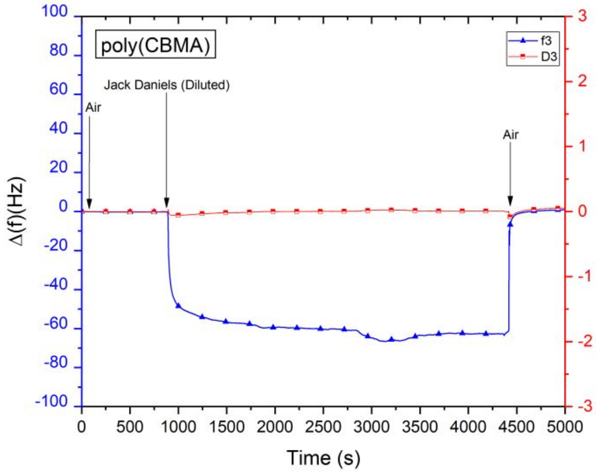
poly(CBMA)
f3
D3
Jack Daniels (Diluted)
Air
Air
Δ(f)(Hz)
Δ(D)(ppm)
Time (s)

	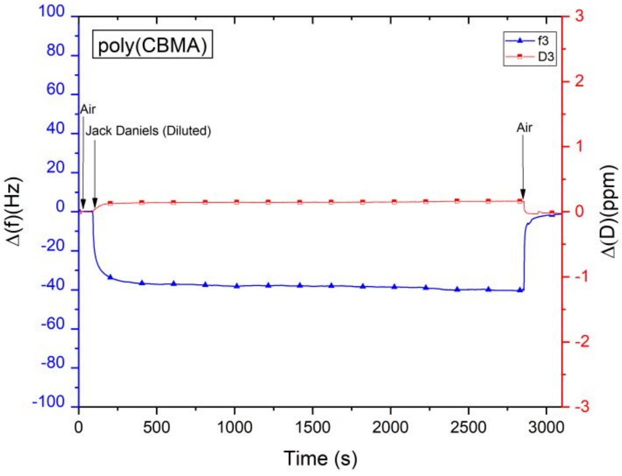
Poly(MeOEGMA)	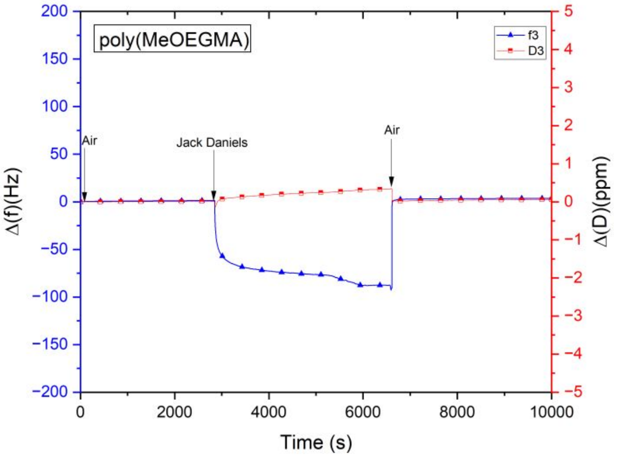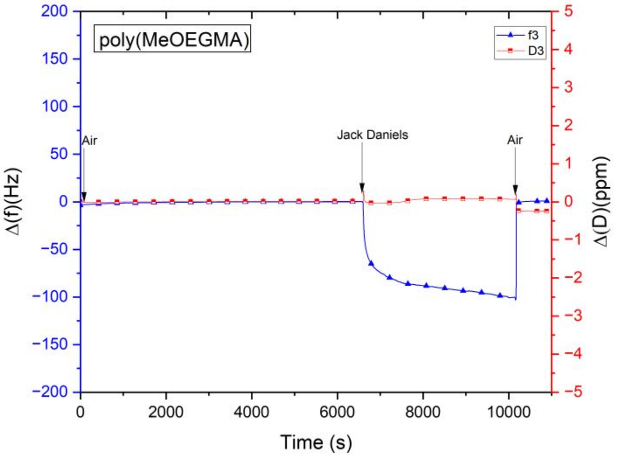

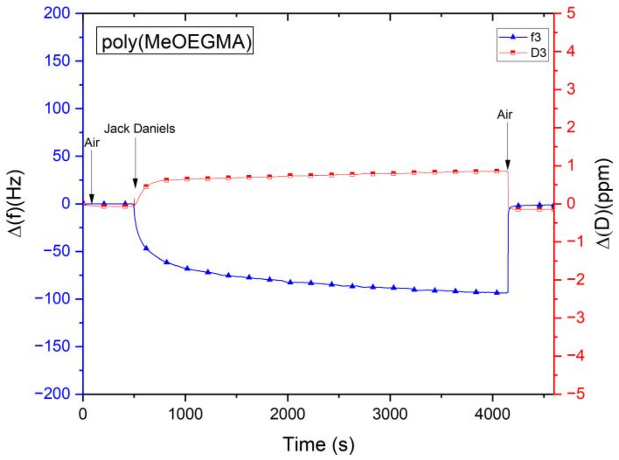

poly(MeOEGMA)
f3
D3
Air
Jack Daniels
Air
Δ(f)(Hz)
Δ(D)(ppm)
Time (s)

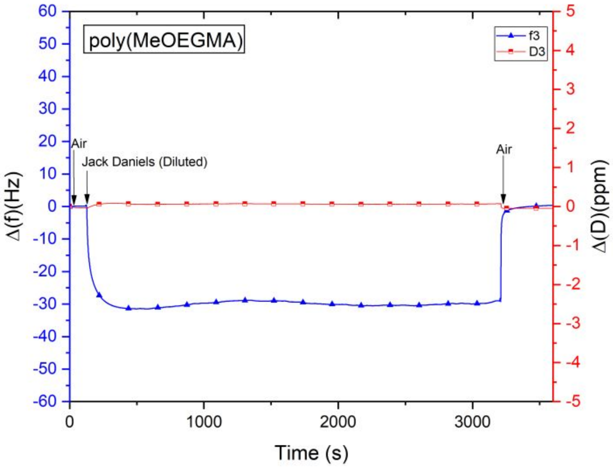

poly(MeOEGMA)
f3
D3
Air
Jack Daniels (Diluted)
Air
Δ(f)(Hz)
Δ(D)(ppm)
Time (s)

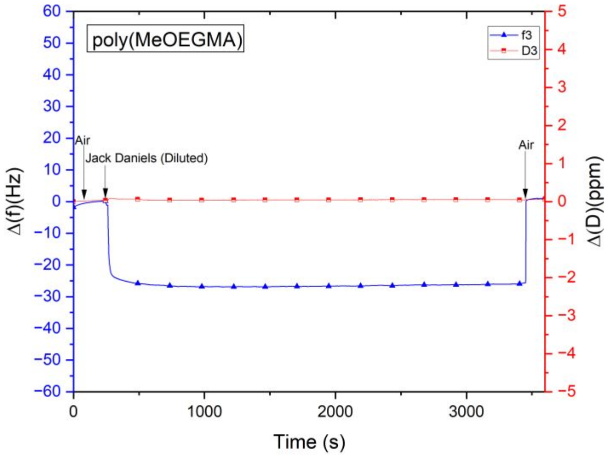

poly(MeOEGMA)
f3
D3
Air
Jack Daniels (Diluted)
Air
Δ(f)(Hz)
Δ(D)(ppm)
Time (s)

Poly(BMA)	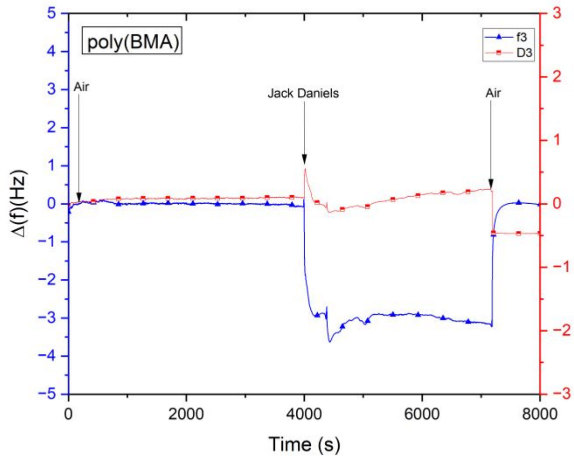 poly(BMA)<br>f3<br>D3<br>Air<br>Jack Daniels<br>Air<br>Δ(f)(Hz)<br>Δ(D)(ppm)<br>Time (s) 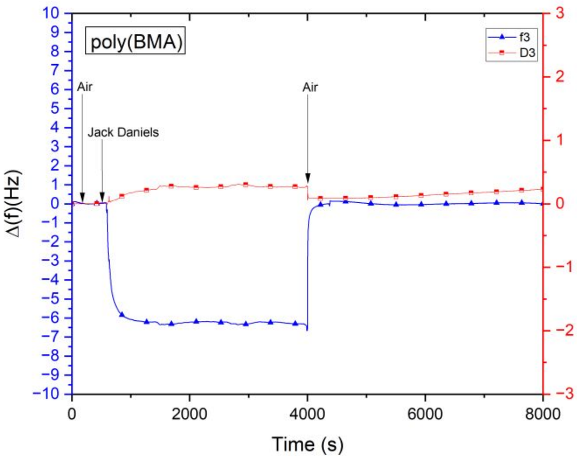 poly(BMA)<br>f3<br>D3<br>Air<br>Jack Daniels<br>Air<br>Δ(f)(Hz)<br>Δ(D)(ppm)<br>Time (s) 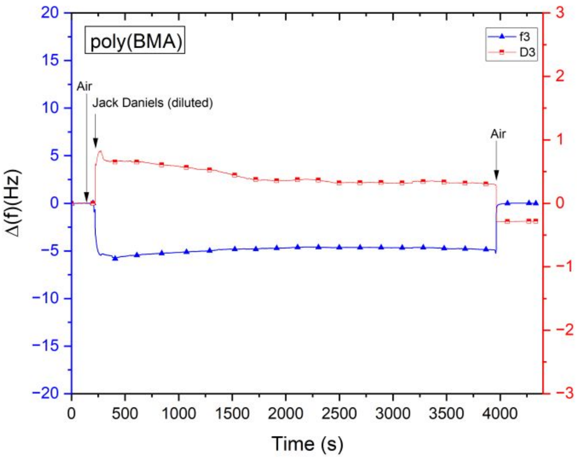 poly(BMA)<br>f3<br>D3<br>Air<br>Jack Daniels (diluted)<br>Air<br>Δ(f)(Hz)<br>Δ(D)(ppm)<br>Time (s)

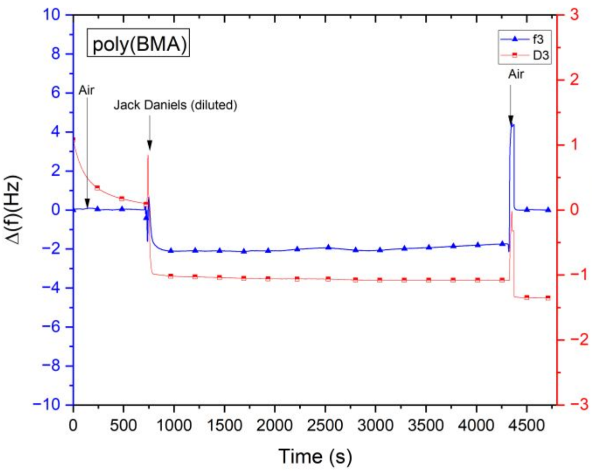
poly(BMA)
f3
D3
Air
Jack Daniels (diluted)
Air
Δ(f)(Hz)
Δ(D)(ppm)
Time (s)

SiO_2

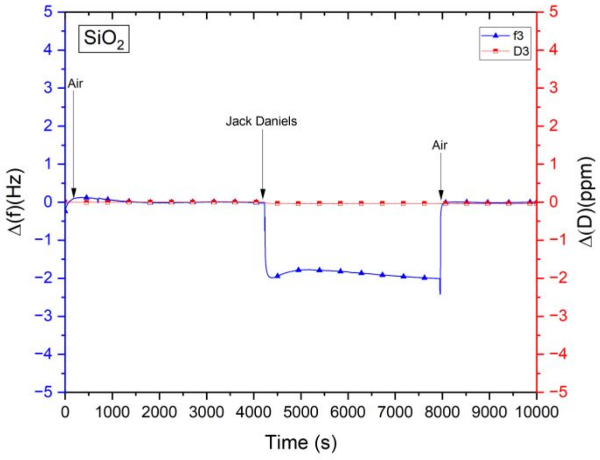
SiO2
f3
D3
Air
Jack Daniels
Air
Δ(f)(Hz)
Δ(D)(ppm)
Time (s)

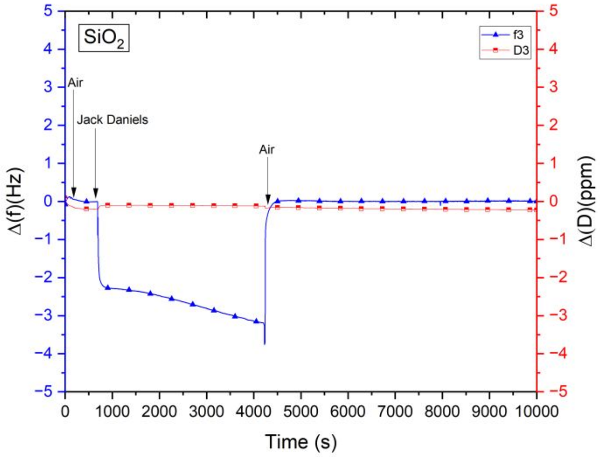
SiO2
f3
D3
Air
Jack Daniels
Air
Δ(f)(Hz)
Δ(D)(ppm)
Time (s)

Table S18. Representative QCM-D sensorgrams of exposure of different polymer brush coatings exposed to different vapors of ethanol (12 ppt) propanol (5 ppt), Jack Daniels whiskey, Jameson whiskey, and lavender oil. The error ranges when corresponding vapors exposed to the coatings separately demotrated by colored boxes ethanol (12 ppt)(blue) propanol (5 ppt)(green), Jack Daniels whiskey(brown), and lavender oil (purple).

<table>
<tr><th>Surface</th><th>Representative QCM-D sensograms</th></tr>
<tr><td>Poly(HPMA)</td><td>
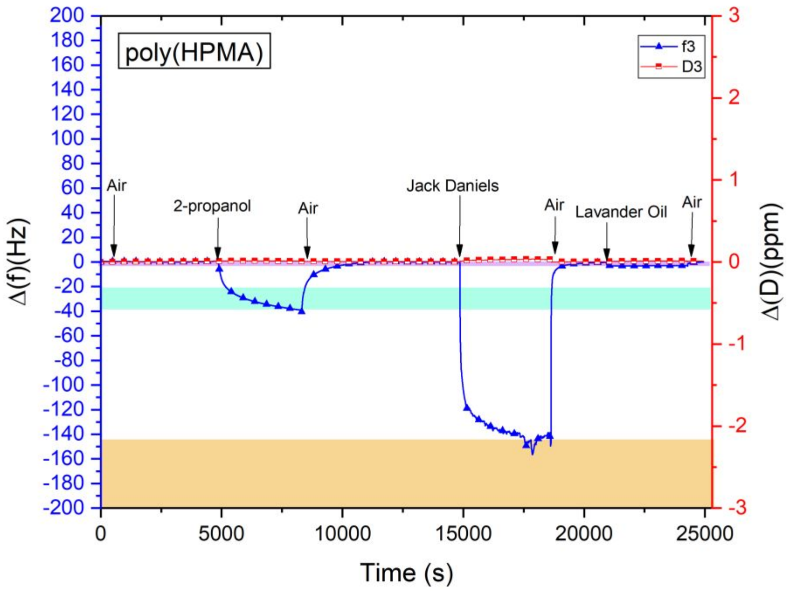

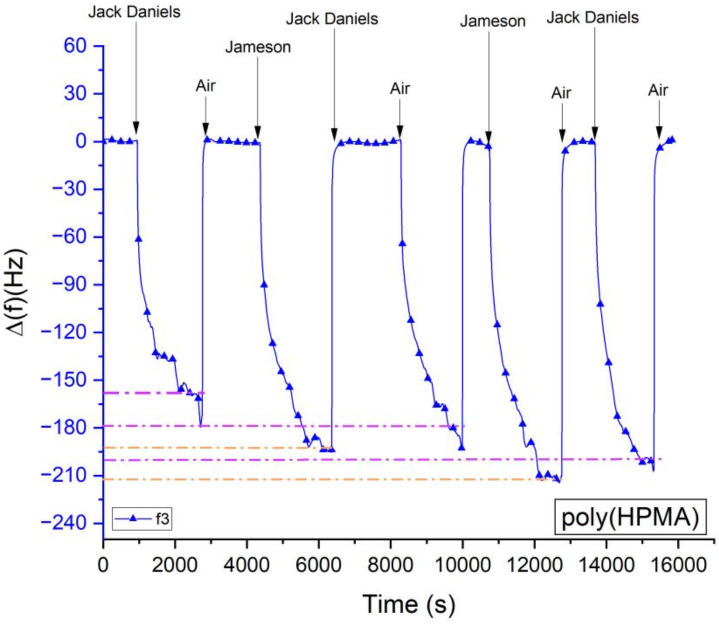

</td></tr>
</table>

Poly(CBMA)	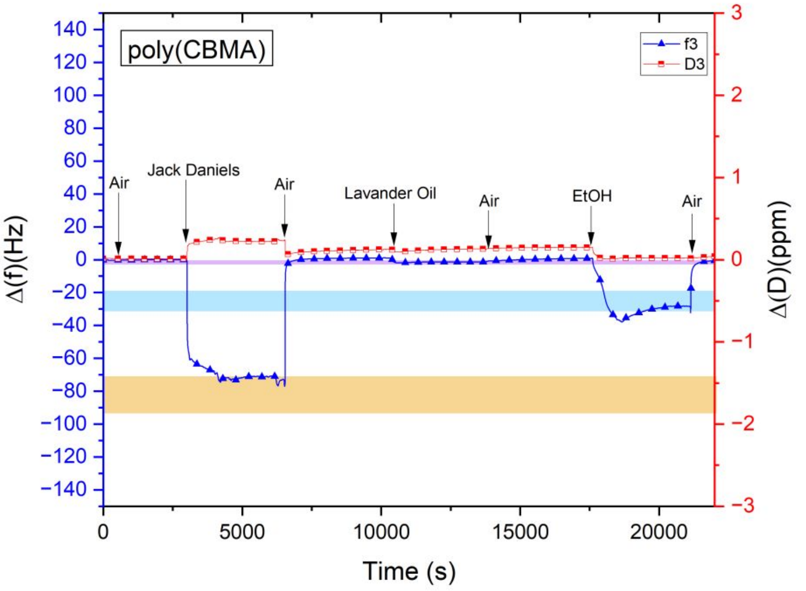
Poly(MeOEGMA)	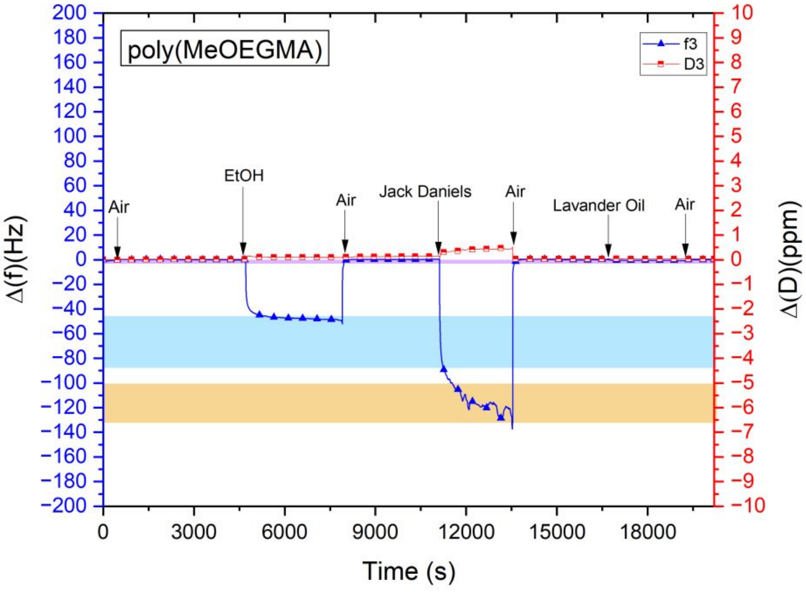

Poly(BMA)	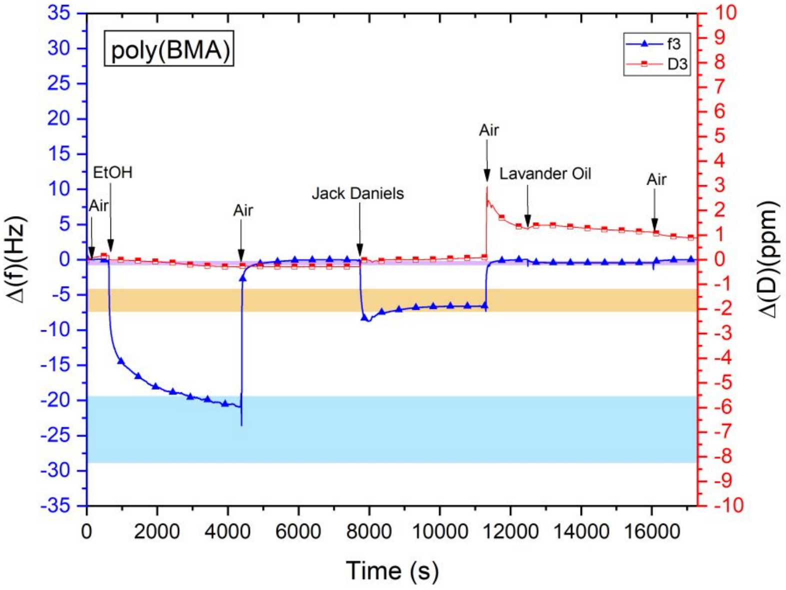 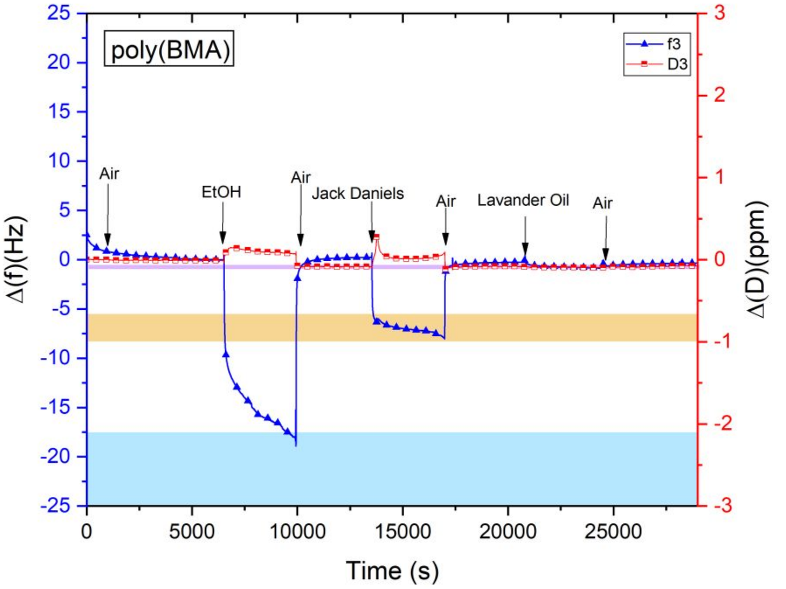

poly(BMA)
f3
D3
Air
EtOH
Air
Jack Daniels
Air
Lavander Oil
Air
Δ(f)(Hz)
Δ(D)(ppm)
Time (s)

poly(BMA)
f3
D3
Air
EtOH
Air
Jack Daniels
Air
Lavander Oil
Air
Δ(f)(Hz)
Δ(D)(ppm)
Time (s)

Table S19. Adsorption of different vapors on the bare silicon oxide surface of the QCM-D chip.

Vapor	Δ(f) (Hz)	Error (Hz)	m (ng·cm^2)	Error (Hz)
Ethanol	5,00	1,00	88,50	17,70
Rosmari Oil	0,00	0,00	0,00	0,00
(S)-(-)-Limonene	0,00	0,00	0,00	0,00
α-Pinene	0,15	0,05	2,66	0,89
Lavander Oil	0,00	0,00	0,00	0,00
(S)-(+)-Carvone	0,06	0,06	1,06	1,06
2-propanol	2,15	0,15	38,06	2,66
Jameson	3,78	1,01	66,86	17,95
3-Methyl-1-butanol	0,99	0,04	17,43	0,62
Acetone	4,98	0,88	88,06	15,49
Ethanol/Water 40/60	2,59	0,33	45,84	5,84
Jack Daniels	2,57	0,58	45,40	10,18

Table S20. Adsorption of different vapors on the surface coated with poly(MeOEGMA) brushes.

Vapor	Δ(f) (Hz)	Error (Hz)	m (ng·cm^2)	Error (Hz)
Ethanol	70,67	19,74	1250,80	349,45
Rosmari Oil	2,56	0,03	45,22	0,44
(S)-(-)-Limonene	0,35	0,05	6,20	0,89
α-Pinene	2,00	0,87	35,40	15,33
Lavander Oil	0,82	0,19	14,51	3,36
(S)-(+)-Carvone	0,78	0,15	13,72	2,57
2-propanol	50,50	7,50	893,85	132,75
Jameson	77,30	3,99	1368,24	70,56
3-Methyl-1-butanol	19,52	0,93	345,42	16,37
Acetone	73,63	10,49	1303,19	185,61
EtOH/Water 40/60	126,98	7,04	2247,46	124,52
Jack Daniels	107,83	12,38	1908,67	219,04

Table S21. Adsorption of different vapors on the surface coated with poly(HPMA) brushes.

Vapor	Δ(f) (Hz)	Error (Hz)	m (ng·cm^2)	Error (Hz)
Ethanol	108,40	12,20	1918,68	215,95
Rosmari Oil	0,05	0,05	0,89	0,89
(S)-(-)-Limonene	1,37	0,03	24,25	0,53
α-Pinene	1,29	0,43	22,83	7,69
Lavander Oil	1,13	0,02	19,91	0,44
(S)-(+)-Carvone	2,18	0,59	38,59	10,49
2-propanol	27,50	7,60	486,75	134,45
Jameson	210,70	8,47	3729,39	149,84
3-Methyl-1-butanol	2,81	1,13	49,74	20,00
Acetone	48,47	3,53	857,92	62,48
EtOH/Water 40/60	147,02	5,70	2602,25	100,89
Jack Daniels	184,60	10,92	3267,44	193,21

Table S22. Adsorption of different vapors on the surface coated with poly(CBMA) brushes.

Vapor	Δ(f) (Hz)	Error (Hz)	m (ng·cm^2)	Error (Hz)
Ethanol	23,25	3,68	411,53	65,18
Rosmari Oil	1,98	0,86	34,96	15,13
(S)-(-)-Limonene	2,62	0,98	46,29	17,43
α-Pinene	0,00	0,00	0,00	0,00
Lavander Oil	0,73	0,11	12,83	1,86
(S)-(+)-Carvone	1,01	0,37	17,79	6,46
2-propanol	0,00	0,00	0,00	0,00
Jameson	135,30	4,30	2394,81	76,11
3-Methyl-1-butanol	1,22	0,31	21,59	5,49
Acetone	48,81	2,56	863,98	45,32
EtOH/Water 40/60	114,01	10,73	2018,04	189,99
Jack Daniels	91,65	12,80	1622,21	226,56

Table S23. Adsorption of different vapors on the surface coated with poly(BMA) brushes.

Vapor	Δ(f) (Hz)	Error (Hz)	m (ng·cm^2)	Error (Hz)
Ethanol	23,19	2,99	410,49	52,90
Rosmari Oil	1,76	0,13	31,15	2,30
(S)-(-)-Limonene	0,00	0,00	0,00	0,00
α-Pinene	8,50	1,75	150,45	30,95
Lavander Oil	0,40	0,15	6,99	2,57
(S)-(+)-Carvone	0,85	0,25	15,05	4,43
2-propanol	25,10	0,20	444,27	3,54
Jameson	5,84	0,26	103,31	4,59
3-Methyl-1-butanol	10,70	0,05	189,39	0,88
Acetone	57,45	6,58	1016,78	116,55
Ethanol/Water 40/60	4,92	1,30	87,14	23,04
Jack Daniels	5,12	0,46	90,68	8,22

Table S24. The concentration of different single compound vapors as measured by Ion Science MiniPID2 Sensor.

	(ppt)	Error (ppt)	(mg·L^{-1})	Error (mg·L^{-1})
Ethanol	12,23	0,53	23	1
(S)-(-)-Limonene	0,60	0,10	3	0
(S)-(+)-Carvone	1,71	0,10	10	0
α-Pinene	1,20	0,13	7	1
2-propanol	4,92	1,01	12	2
acetone	1,90	0,10	5	0
3-Methyl-1-butanol	1,19	0,10	4	0

Table S25. The concentration of different single compound vapors and diluted vapors wit dry air as measured by Ion Science MiniPID2 Sensor.

	(ppt)	Error (ppt)
Ethanol	12,23	0,53
Ethanol (diluted)	9,70	0,09
α-Pinene	1,20	0,13
α-Pinene (diluted)	0,94	0,08

Table S26. Representative QCM-D sensorgrams of exposure of different polymer brush coatings to diluted with dry air ethanol vapor (10 ppt).

Surface	Representative QCM-D sensograms
Poly(HPMA)	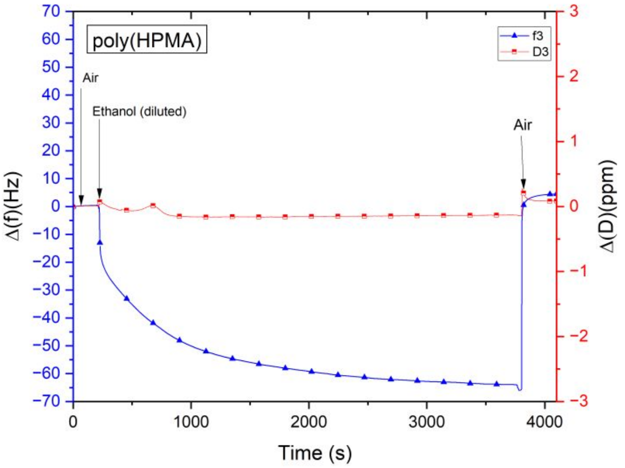 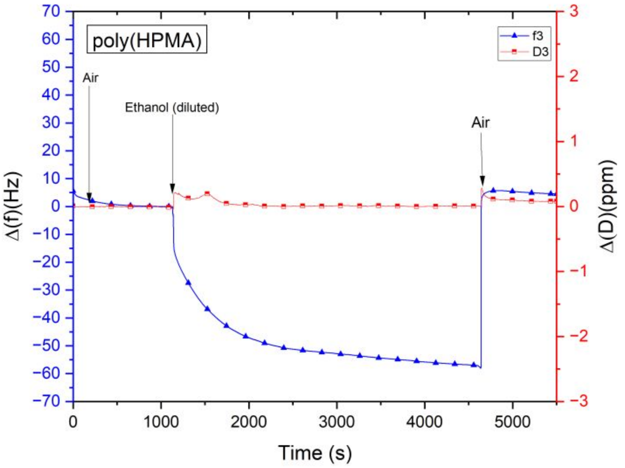

Poly(CBMA)	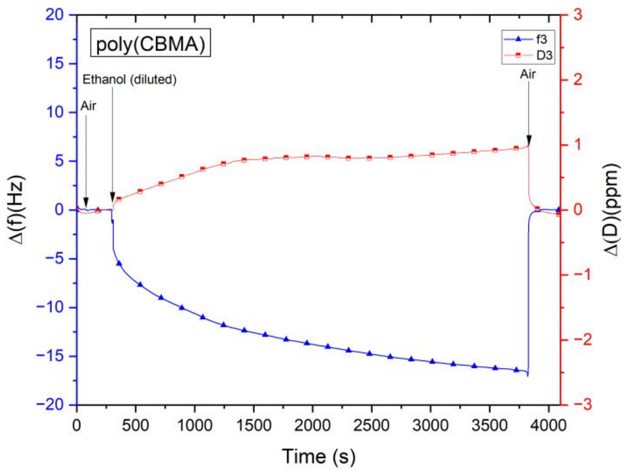<br>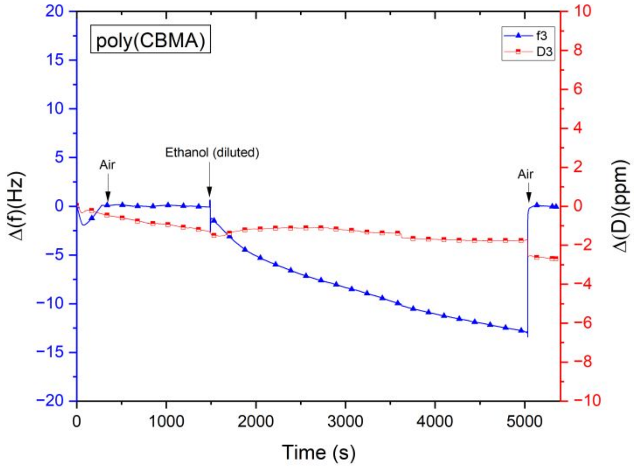
Poly(MeOEGMA)	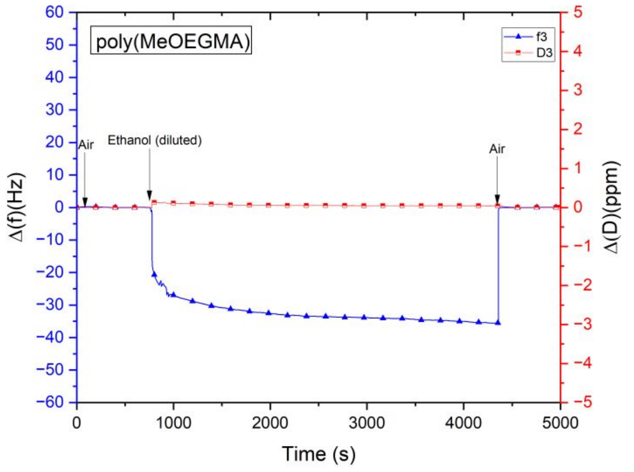

	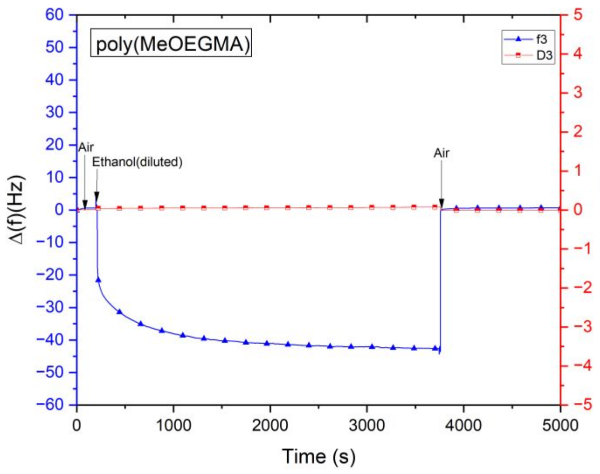
Poly(BMA)	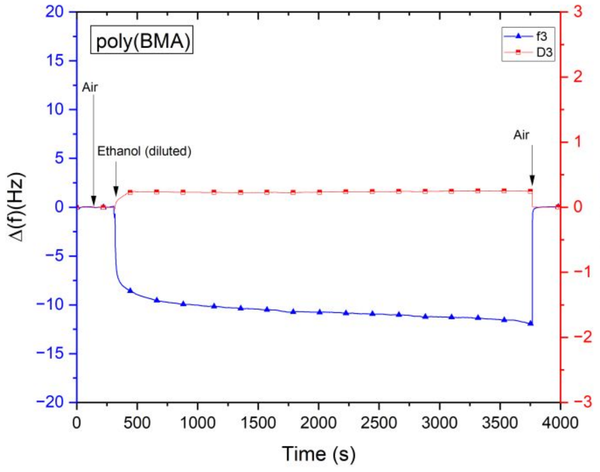

poly(MeOEGMA)
f3
D3
Air
Ethanol(diluted)
Air
Δ(f)(Hz)
Δ(D)(ppm)
Time (s)

poly(BMA)
f3
D3
Air
Ethanol (diluted)
Air
Δ(f)(Hz)
Δ(D)(ppm)
Time (s)

Table S27. Representative QCM-D sensorgrams of exposure of different polymer brush coatings to diluted with dry air α-pinene vapor (0.9 ppt).

<table>
<tr><th>Surface</th><th>Representative QCM-D sensograms</th></tr>
<tr><td>Poly(HPMA)</td><td>
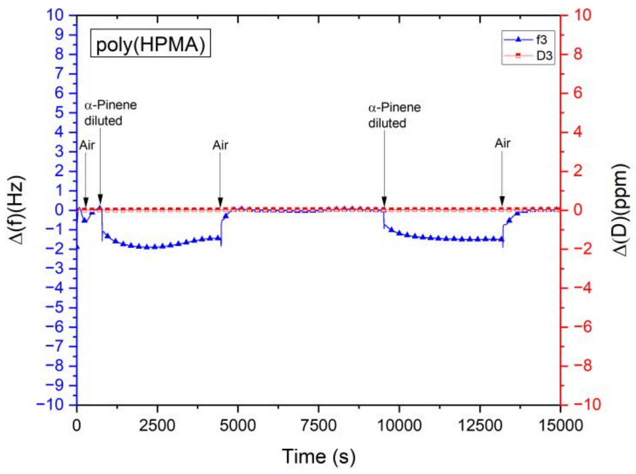

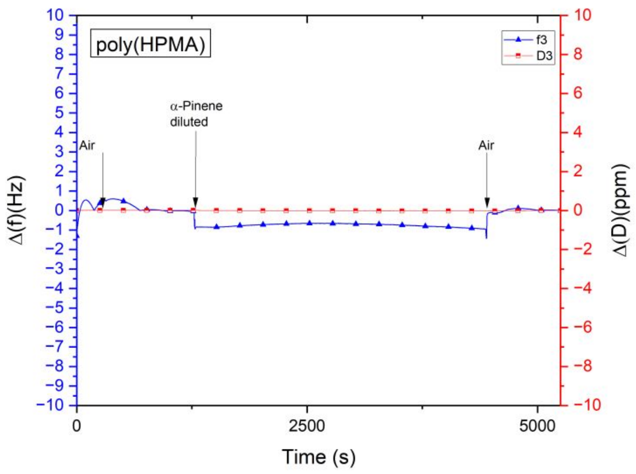

</td></tr>
</table>

Poly(CBMA)	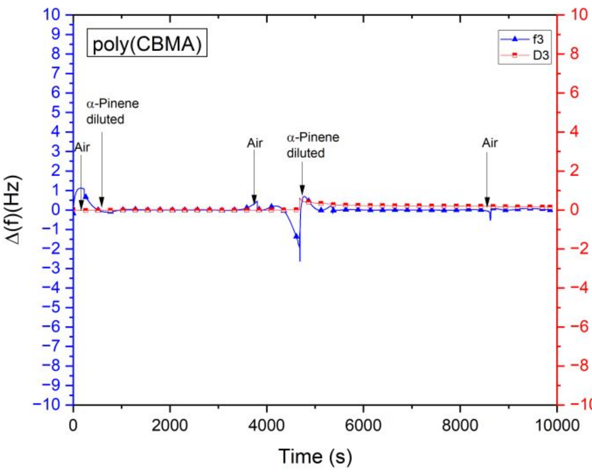 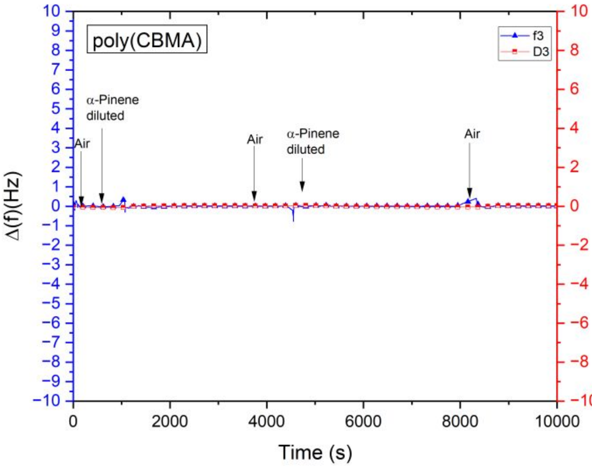
Poly(MeOEGMA)	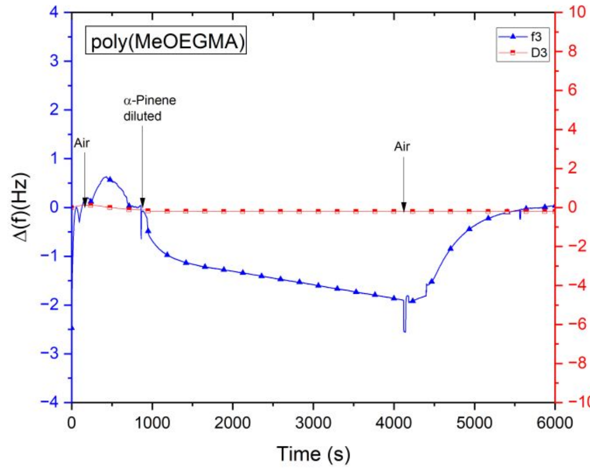

Poly(BMA)	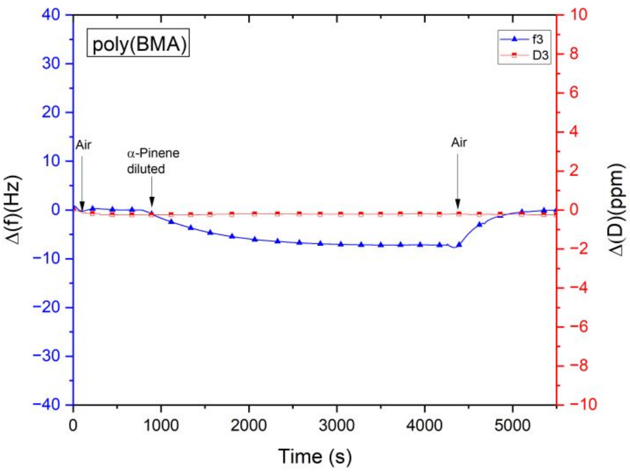poly(BMA)
f3
D3
Air
α-Pinene diluted
Air
Δ(f)(Hz)
Δ(D)(ppm)
Time (s)
40
30
20
10
0
−10
−20
−30
−40
10
8
6
4
2
0
−2
−4
−6
−8
−10
0
1000
2000
3000
4000
5000

Table S28. Representative QCM-D sensorgrams of exposure of different polymer brush coatings to diluted with dry air lavender oil vapor.

Surface	Representative QCM-D sensograms
Poly(HPMA)	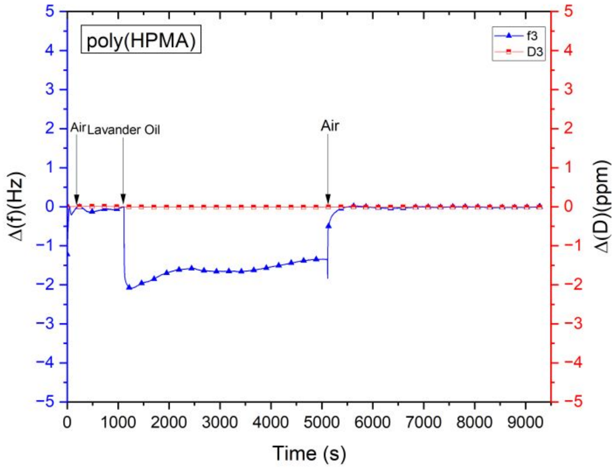 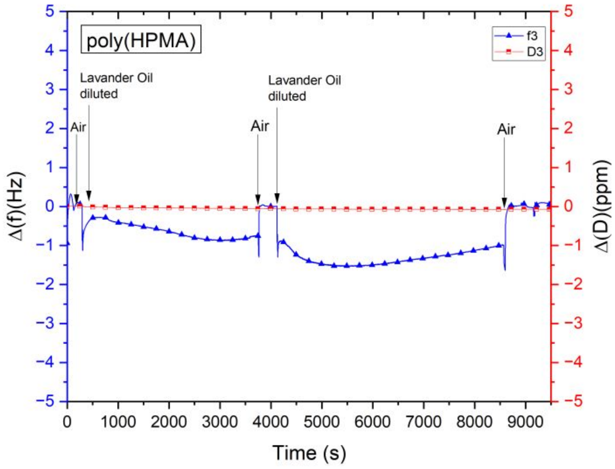

Poly(CBMA)	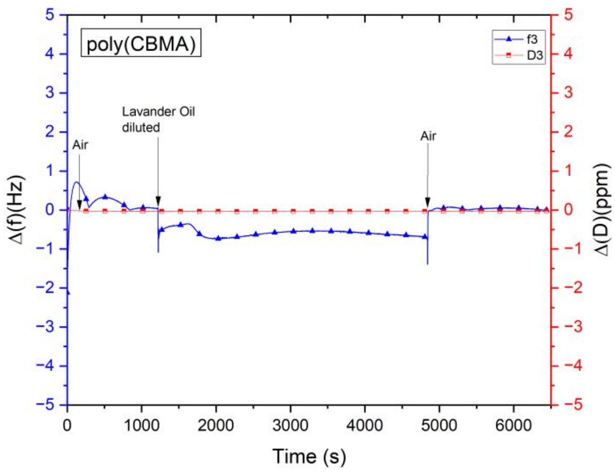poly(CBMA)
f3
D3
Lavander Oil diluted
Air
Air
Δ(f)(Hz)
Δ(D)(ppm)
Time (s) 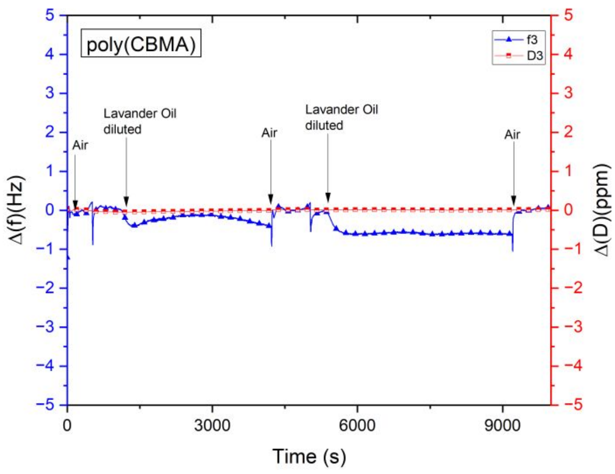poly(CBMA)
f3
D3
Lavander Oil diluted
Lavander Oil diluted
Air
Air
Air
Δ(f)(Hz)
Δ(D)(ppm)
Time (s)

Poly(MeOEGMA)	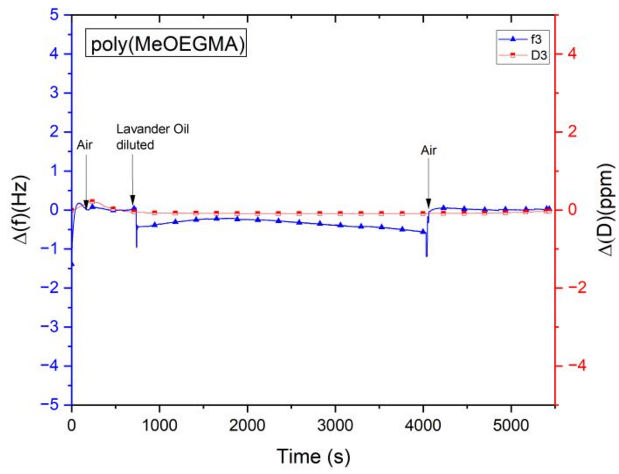 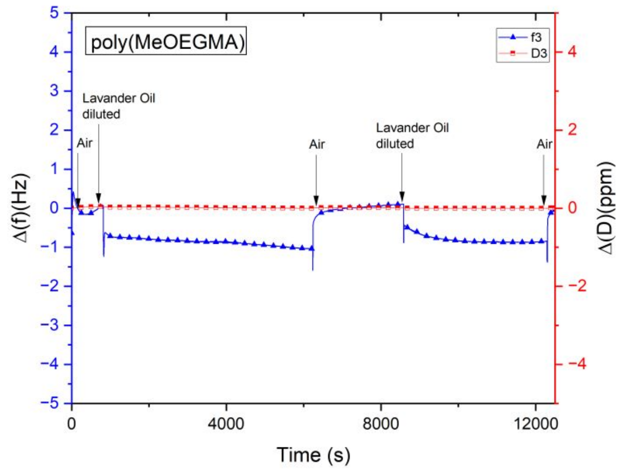
Poly(BMA)	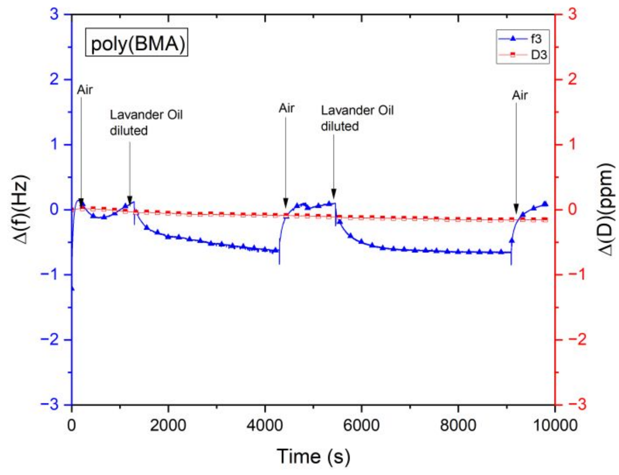

	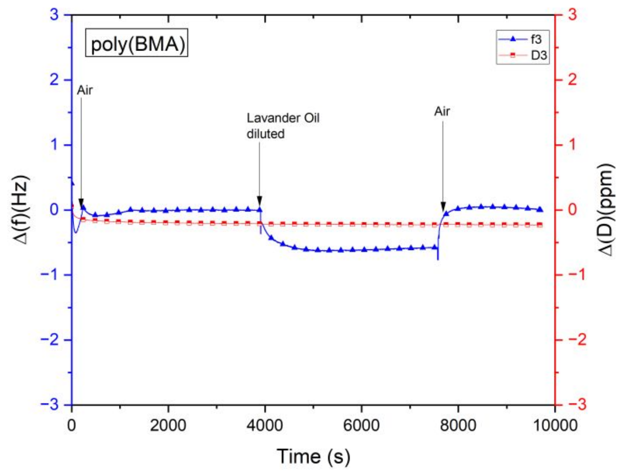poly(BMA)
f3
D3
Air
Lavander Oil
diluted
Air
Δ(f)(Hz)
Δ(D)(ppm)
Time (s)
3
2
1
0
−1
−2
−3
0
2000
4000
6000
8000
10000

Table S29. Representative QCM-D sensorgrams of exposure of different polymer brush coatings to diluted with dry air ethanol-water mixture vapor.

Surface	Representative QCM-D sensograms
Poly(HPMA)	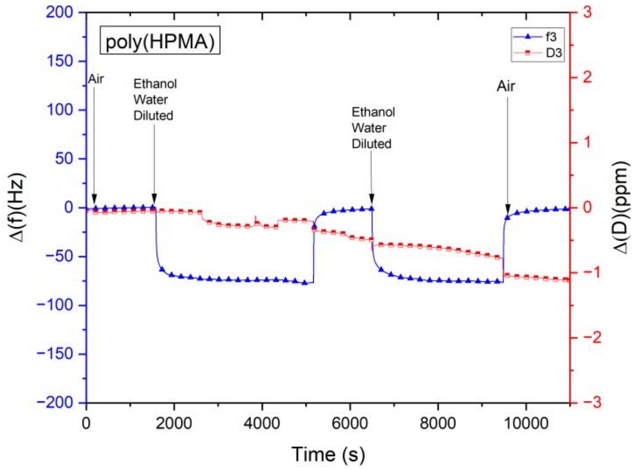<br>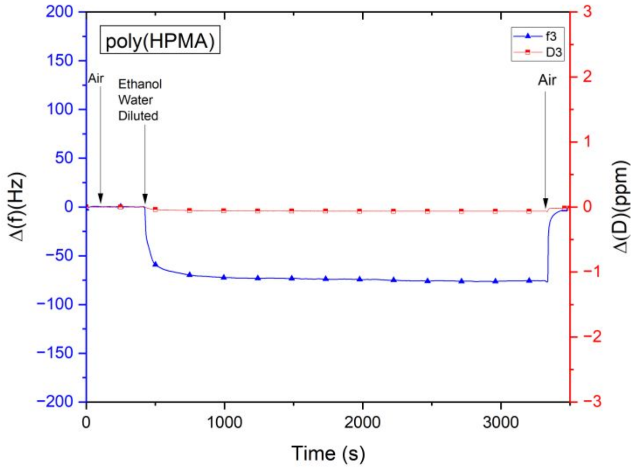
Poly(CBMA)	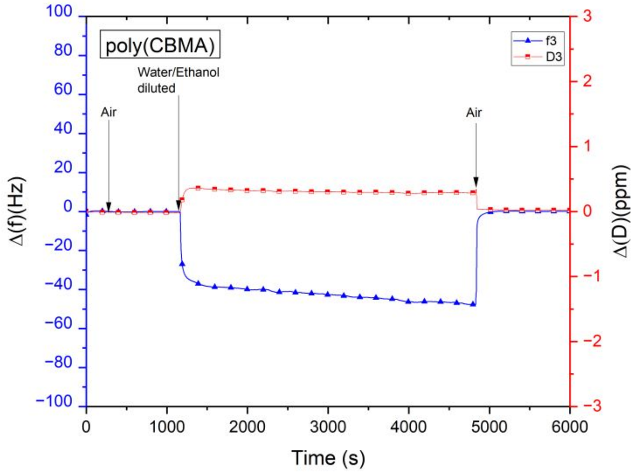

<table>
<tr><td></td><td>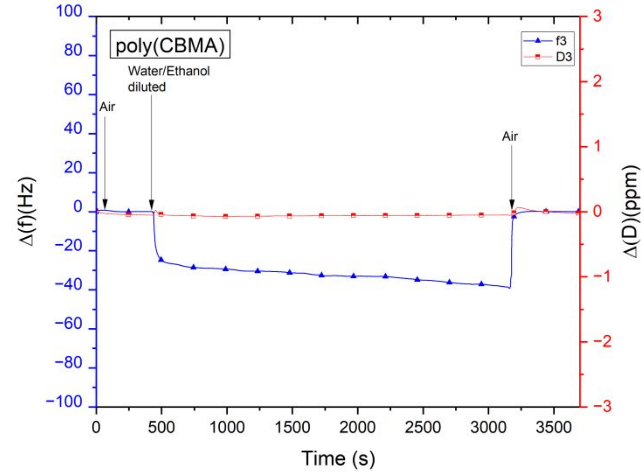
</td></tr>
<tr><td>Poly(MeOEGMA)</td><td>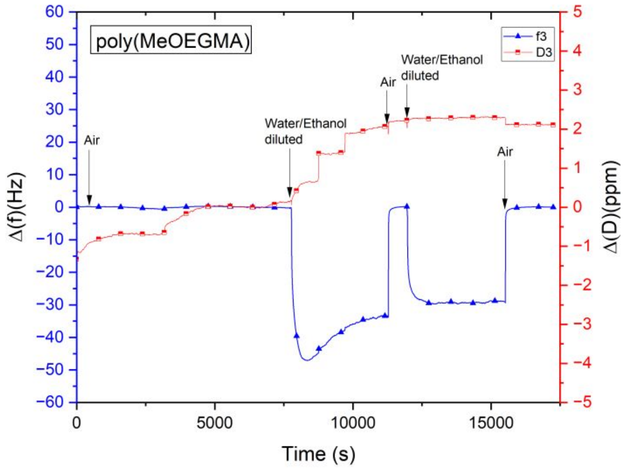

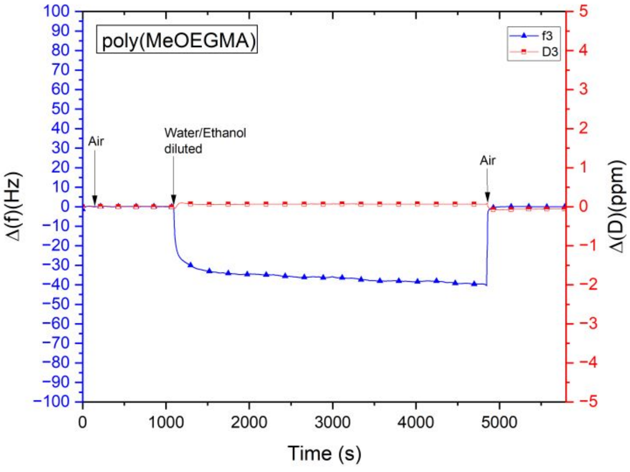
</td></tr>
</table>

Poly(BMA)

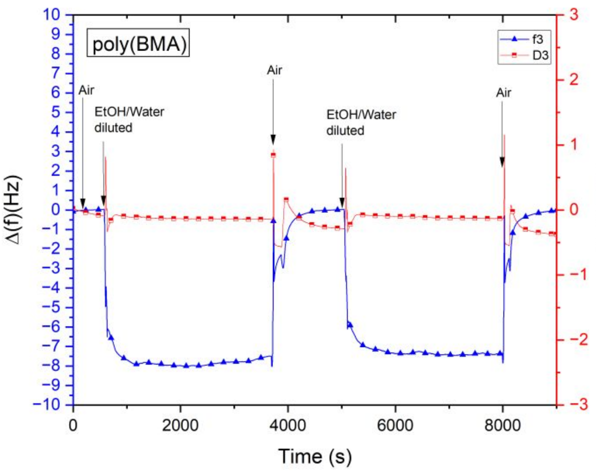
poly(BMA)
f3
D3
Air
EtOH/Water diluted
Air
EtOH/Water diluted
Air
Δ(f)(Hz)
Δ(D)(ppm)
Time (s)

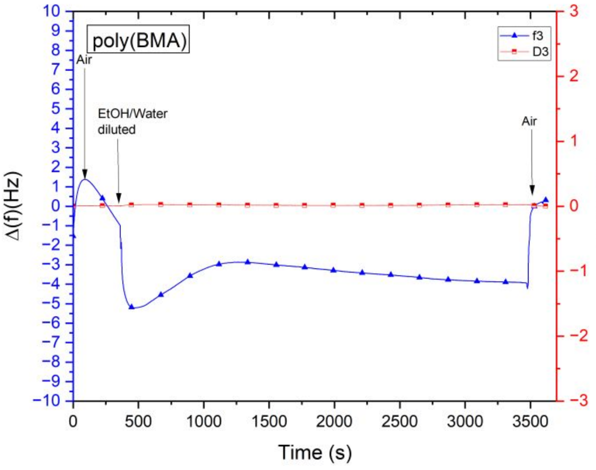
poly(BMA)
f3
D3
Air
EtOH/Water diluted
Air
Δ(f)(Hz)
Δ(D)(ppm)
Time (s)

Table S30. Representative QCM-D sensorgrams of exposure of different polymer brush coatings to diluted with dry air Jack Daniels whiskey vapor.

Surface	Representative QCM-D sensograms
Poly(HPMA)	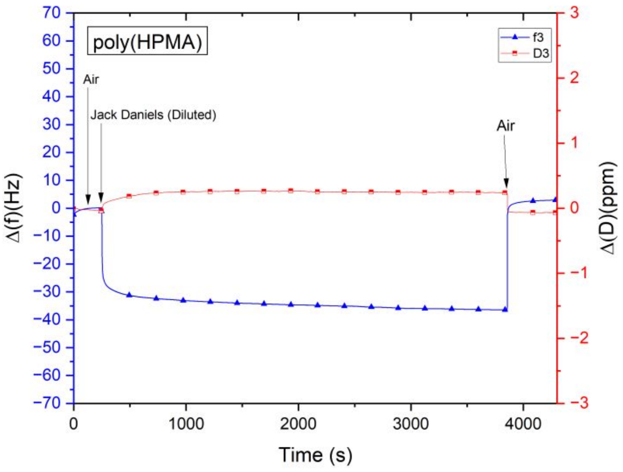<br>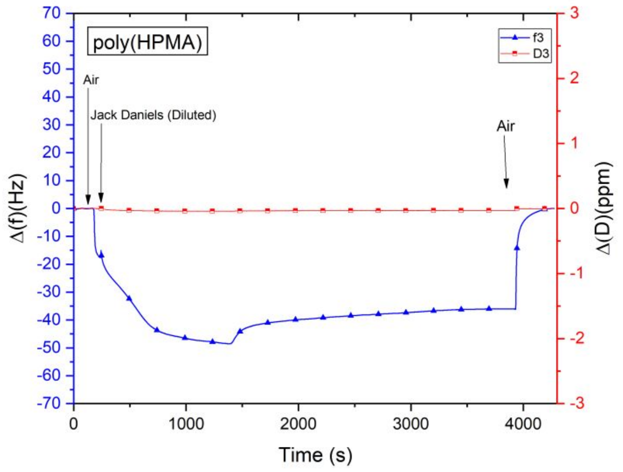

Poly(CBMA)

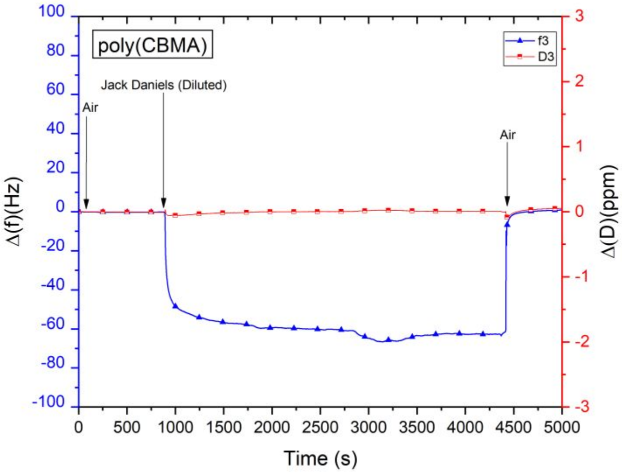

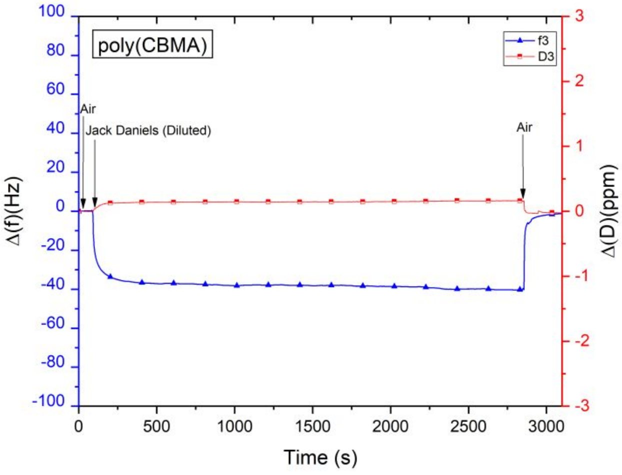

Poly(MeOEGMA)

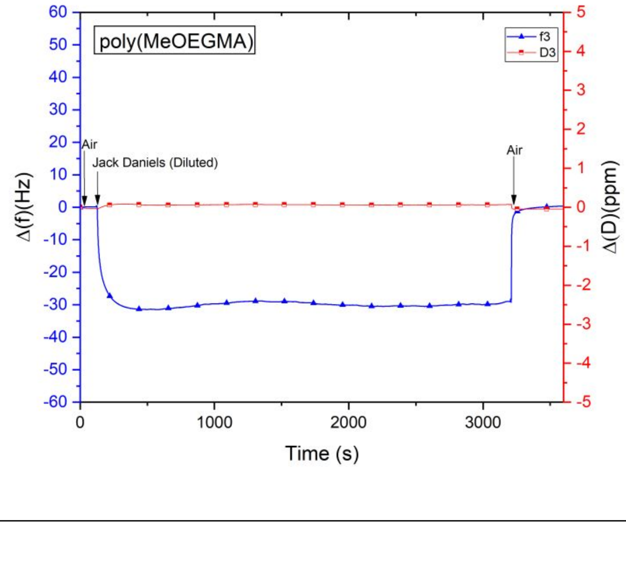

	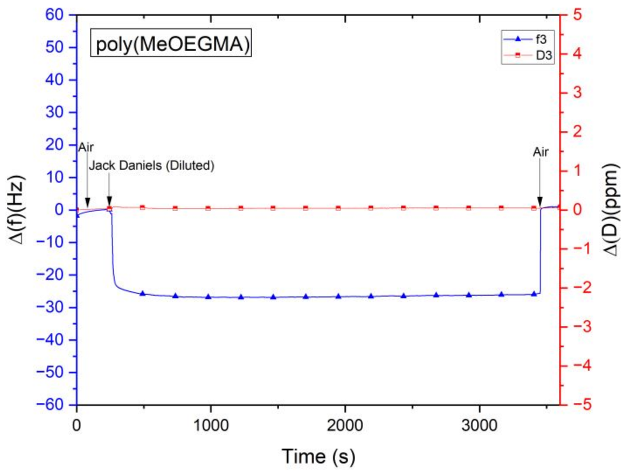poly(MeOEGMA)<br>f3<br>D3<br>Air<br>Jack Daniels (Diluted)<br>Air<br>Δ(f)(Hz)<br>Δ(D)(ppm)<br>Time (s)
Poly(BMA)	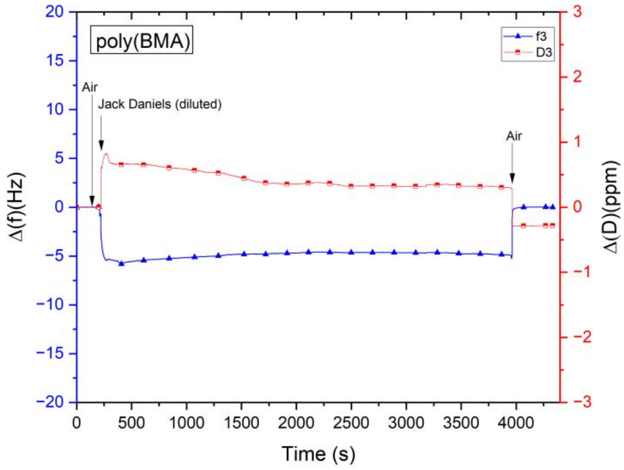poly(BMA)<br>f3<br>D3<br>Air<br>Jack Daniels (diluted)<br>Air<br>Δ(f)(Hz)<br>Δ(D)(ppm)<br>Time (s)<br>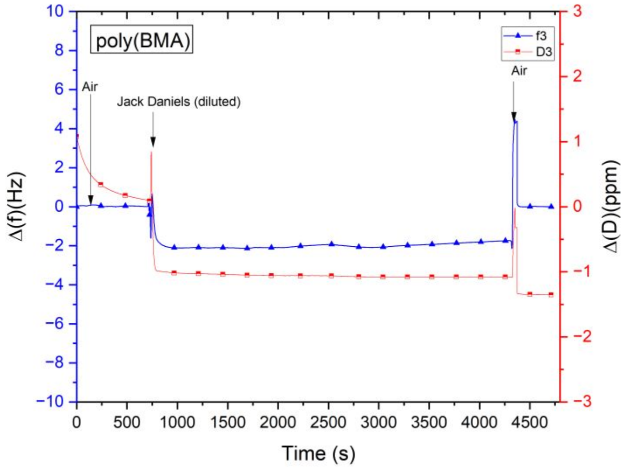poly(BMA)<br>f3<br>D3<br>Air<br>Jack Daniels (diluted)<br>Air<br>Δ(f)(Hz)<br>Δ(D)(ppm)<br>Time (s)

Table S31. Adsorption of different vapors on the surface coated with polymer brushes in QCM-D in Δ(f) (Hz).

Vapor	Poly(MeOEGMA)		Poly(HPMA)		Poly(CBMA)		Poly(BMA)	
	Δ(f) (Hz)	Error (Hz)	Δ(f) (Hz)	Error (Hz)	Δ(f) (Hz)	Error (Hz)	Δ(f) (Hz)	Error (Hz)
Ethanol	70,67	19,74	108,40	12,20	23,25	3,68	23,19	2,99
Ethanol (diluted)	38,99	3,63	60,40	3,49	14,51	1,84	10,56	1,01
α-Pinene	2,00	0,87	1,29	0,43	0,00	0,00	8,50	1,75
α-Pinene (diluted)	1,8	0,2	1,2	0,2	0,0	0,0	6,7	0,4
Jack Daniels	107,83	12,38	184,60	10,92	91,65	12,80	5,12	0,46
Jack Daniels (diluted)	28,75	1,34	36,21	0,20	29,79	5,25	2,36	0,84
Lavander Oil	0,82	0,19	1,13	0,02	0,73	0,11	0,40	0,15
Lavander Oil (diluted)	0,7	0,1	1,0	0,1	0,5	0,1	0,4	0,1

Table S31. Adsorption of different vapors on the surface coated with polymer brushes in QCM-D in Δ(f) (Hz).

Vapor	Poly(MeOEGMA)		Poly(HPMA)		Poly(CBMA)		Poly(BMA)	
	m (ng·cm2)	Error (ng·cm2)	m (ng·cm2)	Error (ng·cm2)	m (ng·cm2)	Error (ng·cm2)	m (ng·cm2)	Error (ng·cm2)
Ethanol	1250,8	349,4	1918,7	216,0	411,5	65,2	410,5	52,9
Ethanol (diluted)	690,1	64,3	1069,1	61,8	256,7	32,5	186,9	17,9
α-Pinene	35,4	15,3	22,8	7,7	0,0	0,0	150,5	30,9
α-Pinene (diluted)	31,9	3,2	20,5	2,8	0,0	0,0	118,9	7,7
Jack Daniels	1908,7	219,0	3267,4	193,2	1622,2	226,6	90,7	8,2
Jack Daniels (diluted)	508,9	23,8	640,9	3,5	527,2	92,9	41,7	14,9
Lavander Oil	14,5	3,4	19,9	0,4	12,8	1,9	7,0	2,6
Lavander Oil (diluted)	12,5	1,9	18,3	2,6	8,4	1,6	7,6	0,9

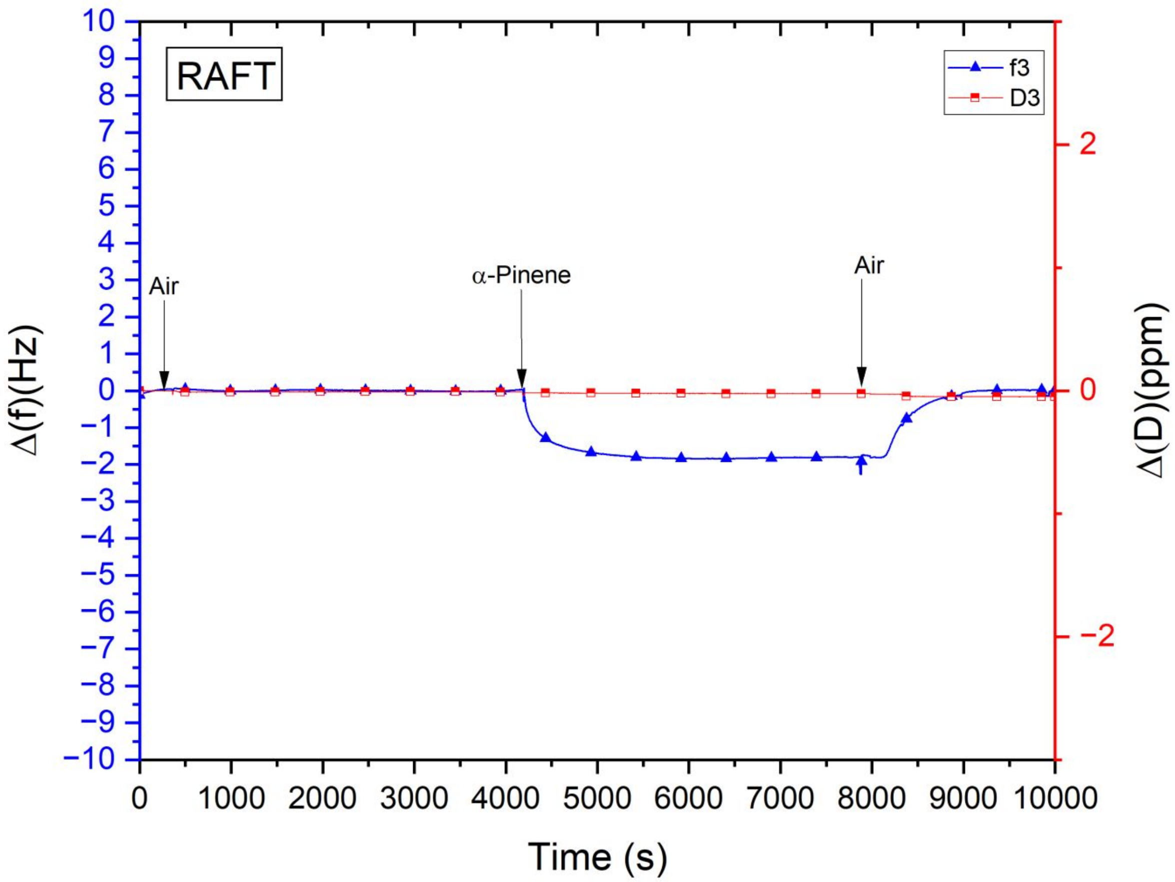

Figure S1. Representative QCM-D sensorgram of exposure of RAFT-monolayer coatings to α-Pinene (1 ppt) vapor.

References

(1) Kuzmyn, A. R.; Nguyen, A. T.; Teunissen, L. W.; Zuilhof, H.; Baggerman, J. Antifouling Polymer Brushes via Oxygen-Tolerant Surface-Initiated PET-RAFT. *Langmuir* **2020**, *36* (16), 4439-4446.

(2) Kuzmyn, A. R.; van Galen, M.; van Lagen, B.; Zuilhof, H. SI-PET-RAFT in flow: improved control over polymer brush growth. *Polymer Chemistry* **2023**, *14* (29), 3357-3363, 10.1039/D3PY00488K.

(3) Kuzmyn, A. R.; Teunissen, L. W.; Kroese, M. V.; Kant, J.; Venema, S.; Zuilhof, H. Antiviral Polymer Brushes by Visible-Light-Induced, Oxygen-Tolerant Covalent Surface Coating. *ACS Omega* **2022**, *7* (43), 38371-38379.

(4) Kuzmyn, A. R.; Ypma, T. G.; Zuilhof, H. Tunable Cell-Adhesive Surfaces by Surface-Initiated Photoinduced Electron-Transfer-Reversible Addition–Fragmentation Chain-Transfer Polymerization. *Langmuir* **2024**, *40* (7), 3354-3359.

(5) Kuzmyn, A. R.; Teunissen, L. W.; Fritz, P.; van Lagen, B.; Smulders, M. M. J.; Zuilhof, H. Diblock and Random Antifouling Bioactive Polymer Brushes on Gold Surfaces by Visible-Light-Induced Polymerization (SI-PET-RAFT) in Water. *Advanced Materials Interfaces* **2022**, *9* (3), 2101784.

(6) Fairley, N.; Fernandez, V.; Richard-Plouet, M.; Guillot-Deudon, C.; Walton, J.; Smith, E.; Flahaut, D.; Greiner, M.; Biesinger, M.; Tougaard, S.; et al. Systematic and collaborative approach to problem solving using X-ray photoelectron spectroscopy. *Applied Surface Science Advances* **2021**, *5*, 100112.

(7) Pueyo Bellafont, N.; Viñes, F.; Hieringer, W.; Illas, F. Predicting core level binding energies shifts: Suitability of the projector augmented wave approach as implemented in VASP. *Journal of Computational Chemistry* **2017**, *38* (8), 518-522.

(8) Perdew, J. P. Density-functional approximation for the correlation energy of the inhomogeneous electron gas. *Physical Review B* **1986**, *33* (12), 8822-8824.

(9) Becke, A. D. Density-functional exchange-energy approximation with correct asymptotic behavior. *Physical Review A* **1988**, *38* (6), 3098-3100.

(10) Snijders, J. G., Baerends, E. J.; Vernooijs, P. *At. Nucl. Data Tables* **1982**, *26*, 483.

(11) te Velde, G.; Bickelhaupt, F. M.; Baerends, E. J.; Fonseca Guerra, C.; van Gisbergen, S. J. A.; Snijders, J. G.; Ziegler, T. Chemistry with ADF. *Journal of Computational Chemistry* **2001**, *22* (9), 931-967.

(12) Nečas, D.; Klapetek, P. Gwyddion: an open-source software for SPM data analysis. *Central European Journal of Physics* **2012**, *10* (1), 181-188.

(13) Sauerbrey, G. Verwendung von Schwingquarzen zur Wägung dünner Schichten und zur Mikrowägung. *Zeitschrift für Physik* **1959**, *155* (2), 206-222.
